# Impact of *Pediococcus pentosaceus* YF01 on the exercise capacity of mice through the regulation of oxidative stress and alteration of gut microbiota

**DOI:** 10.3389/fmicb.2024.1421209

**Published:** 2024-06-26

**Authors:** Xiaoguang Yang, Yeni Wang, Yuhua Yang

**Affiliations:** ^1^School of Physical Education, Yan’an University, Yan’an, Shaanxi, China; ^2^Ministry of Sports, Xiamen Institute of Technology, Xiamen, Fujian, China; ^3^Department of Social Sports Management, College of Humanities and Law, Beijing University of Chemical Technology, Beijing, China

**Keywords:** *Pediococcus pentosaceus*, gut microbiota, oxidative stress, exercise capacity, lactic acid bacteria

## Abstract

Using treadmill training, this study replicated human exercise conditions and triggered exercise-induced fatigue in mice to examine the potential of *Pediococcus pentosaceus* YF01 in delaying this fatigue by regulating oxidative stress and its impact on the exercise capacity and gut microbiota of mice. The exercise capacity of mice was tested by conducting exhaustion tests, determining histopathological changes in mouse tissues, detecting the levels of serum biochemical markers, and evaluating the mRNA expression levels of relevant genes. YF01 prolonged the exhaustion time of mice, increased the serum levels of oxidative stress-related markers T-AOC, CAT, and GSH, as well as GLU and LA levels in the mice. YF01 decreased the levels of hepatic-related markers AST and ALT, as well as exercise-related markers LDH, BUN, UA, and CRE in the mice. YF01 upregulated the mRNA expression of MyHc I, SIRT1, and PGC in muscle tissues, as well as SOD1, SOD2, and CAT in both liver and muscle tissues. YF01 also downregulated the mRNA expression of MyHc IIa, MyHc IIb, and MyHc IIx in muscle tissues. Furthermore, YF01 increased the abundance of beneficial bacteria such as *Lactobacillus* and *Lachnospiraceae* in the gut microbiota of mice. In conclusion, *P. pentosaceus* YF01 may affect the exercise capacity of mice by modulating oxidative stress levels, thereby offering novel ideas for developing of sports science and human health.

## 1 Introduction

The benefits of exercise for physical and mental health are well-known (Begdache et al., 2020; Das and Gailey, 2022). Exercise has become increasingly crucial because of the advancement of society and the improvement of living standards. Research on exercise capacity has consistently attracted considerable attention. Physical exercise is extensively recognized as an effective means of maintaining physical health and helps in strengthening the body, improving immunity, enhancing cardiovascular and respiratory functions, etc. (Fiuza-Luces et al., 2018; Shao et al., 2021). Furthermore, doping is prohibited among professional athletes (Morente-Sanchez and Zabala, 2013). Therefore, long-term intake of certain foods, such as probiotics, to improve exercise capacity is a more feasible approach. The mouse treadmill model is commonly used in experiments to evaluate the exercise ability, endurance, and coordination of mice (Cai et al., 2018). We used a mouse treadmill model to simulate human exercise training and explore the impact of *Pediococcus pentosaceus* on exercise capacity.

Oxidative stress significantly influences exercise capacity during physical activity (McLeay et al., 2017). It damages proteins, lipids, and DNA by inducing an imbalance in the cellular redox state, characterized by increased levels of reactive oxygen species (ROS) (Lushchak, 2014). Excessive oxidative stress accelerates cellular aging and causes a decline in organ function, thereby affecting the normal metabolism and exercise capacity of the body (Liguori et al., 2018). While performing physical exercise or other forms of activity, an individual’s body is in a high-intensity metabolic state, which elevates cellular oxidative stress levels (Powers and Jackson, 2008). As the physical activity escalates, the level of cellular oxidative stress also increases, which may induce excess ROS production, thereby harming the cells and body (He et al., 2016). On the other hand, moderate oxidative stress promotes the body’s adaptation to continuous exercise, improves exercise performance, and augments physical endurance (Simioni et al., 2018). Therefore, a balance in oxidative stress must be maintained for good human health.

*Pediococcus pentosaceus* is a lactic acid bacteria (LAB) commonly used for producing fermented foods such as kimchi, sauerkraut, and certain types of sourdough bread (Qi et al., 2021). This LAB is known to produce lactic acid, which contributes to food preservation and fermentation (Nanasombat et al., 2017). It is used as a probiotic in various dairy products and supplements because of its potential health benefits, including promoting gut health and boosting the immune system (Erten et al., 2014; Pan et al., 2021). Different *P. pentosaceus* strains may exhibit diverse characteristics and applications in food fermentation and probiotic use. This LAB can modulate oxidative stress levels. *P. pentosaceus* decreased ROS production by affecting the cellular redox equilibrium, thus alleviating the adverse effects of oxidative stress on the body (Kim et al., 2019). Nevertheless, the impact of *P. pentosaceus* on physical performance has not been exhaustively examined. Therefore, this study investigates *P. pentosaceus* YF01 to determine if it affects exercise capacity in mice by modulating oxidative stress.

The intestine is an important organ for maintaining health balance, and there is a close connection between the gut microbiota of mice and their exercise performance. Certain intestinal microbiota can produce excitatory neurotransmitters that promote exercise performance, such as norepinephrine and dopamine, providing energy to intestinal epithelial cells (Bao et al., 2019). Other studies have indicated that there is a close and coordinated relationship between gut microbiota and host metabolism, energy utilization, and storage (Nicholson et al., 2012). Hence, maintaining a healthy gut microbiota may help improve exercise performance. Adjusting diet, supplementing with probiotics, or other methods to promote a balance in gut microbiota can assist in improving metabolic status, enhancing immune function, and consequently improving exercise performance.

Through experimental observation and data analysis on mice, we elucidated the specific mechanisms through which YF01 affects oxidative stress levels and exercise performance in mice. The study findings will contribute to the understanding of the role of oxidative stress during exercise, thereby offering a scientific basis for identifying new strategies for augmenting exercise performance and maintaining good health. By exploring the potential application of YF01 as an oxidative stress modulator in exercise, we intend to contribute to sports science development and provide novel insights and theoretical foundations for human health.

## 2 Materials and methods

### 2.1 Strain preparation

This study used LAB isolated and purified from fermented kimchi in Sangou Village, Dawan Town, Yubei, Chongqing, as the experimental strain. The 16S rDNA analysis revealed that this strain belonged to *P. pentosaceus*, with Gram staining unveiling its colony morphology. China General Microbiological Culture Collection Center (CGMCC, Beijing) has preserved it as *P. pentosaceus* YF01 with preservation number 29920. Before conducting the subsequent experiments, the preserved strain was reactivated by inoculating 2% inoculum in MRS liquid medium for 16–24 h at 37°C.

### 2.2 Tolerance of the experimental strain in artificial gastric fluid

To prepare the artificial gastric fluid, 0.2% NaCl and 0.35% pepsin were used (Beijing Solarbio Biotechnology Co. Ltd., Beijing, China), with the pH of the fluid adjusted to 3.0 with 1 mol/L HCl after preparation. The solution was filtered through a 0.22-μm filter. Subsequently, a 5 ml sample of the activated bacterial culture was centrifuged at 3,000 rpm at room temperature for 10 min. The bacterial cells were collected after discarding the supernatant. They were then washed twice with sterile physiological saline. The cells were resuspended in 5 ml of physiological saline and adjusted to achieve a concentration of 1 × 10^8^ colony-forming units (CFU)/ml. A 0-h time point sample was prepared by mixing the experimental bacterial suspension with the artificial gastric fluid at a 1:9 volumetric ratio. This mixture was shaken well, and 2 ml of the mixture was added to a 5 ml centrifuge tube. In the next step, the remaining 8 ml samples were shaken vertically at 37°C and at 50 rpm for 3 h in a constant-temperature air bath. Using 10-fold dilutions of the samples collected at 0 and 3 h, we determined the viable cell count. After the dilutions were plated on the MRS solid medium and incubated at 37°C for 48 h, CFUs were counted using the plate counting method (Pan et al., 2021). The survival rate was calculated using the following formula: Survival rate (%) = Number of viable cells at 3 h (CFU/ml) / Number of viable cells at 0 h (CFU/ml) × 100.

### 2.3 Evaluation of growth efficiency of an experimental strain in bile salts

Bacterial cultures were inoculated into an MRS-THIO medium containing varying levels of bovine bile salts at a 2% rate. A control medium with 0.00% bovine bile salts in MRS-THIO was also used. For 24 h, the cultures were incubated at 37°C in a constant-temperature incubator. At 600 nm, a spectrophotometer was used to measure the absorbance (OD) of the cultures in media with different concentrations (Pan et al., 2021). The growth efficiency at different bile salt concentrations was calculated as follows: Growth efficiency (%) = Absorbance of medium containing bile salts at 600 nm / Absorbance of blank medium at 600 nm × 100.

### 2.4 Detection of the hydroxyl radical scavenging rate of experimental bacterial strain

A 5 ml activated bacterial culture was centrifuged in a 10 ml centrifuge tube for 10 min at 3,000 rpm. After the supernatant was discarded, bacterial cells were collected, washed twice with sterile physiological saline, resuspended in 5 ml of physiological saline, and adjusted to attain a concentration of 1 × 10^8^ CFU/ml. The experiment was conducted according to the instructions provided in the hydroxyl radical scavenging rate detection kit (Leagene, Beijing, China).

### 2.5 Detection of the DPPH radical scavenging rate of the experimental bacterial strain

The activated bacterial culture was adjusted to 1 × 10^8^ CFU/ml, and the experiment was conducted following the DPPH radical scavenging rate detection kit (Leagene).

### 2.6 Animal model

**FIGURE 1:**
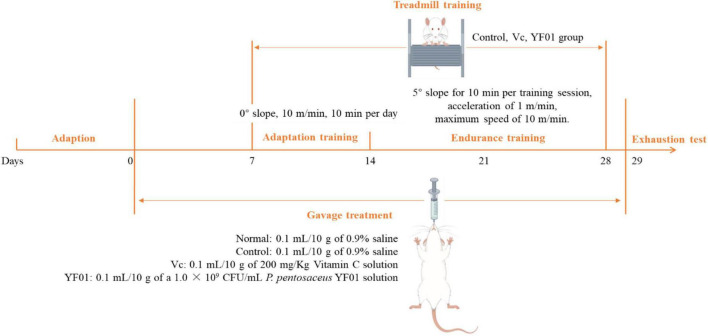
Timeline of the experiment.

Forty Kunming mice (gender: male, age: 5 weeks, weight: 20 ± 2 g) were purchased from Chongqing Enswell Biotechnology Co., Ltd. All mice were housed in a temperature-controlled room (temperature: 25°C ± 2°C, relative humidity: 50% ± 5%, 12-h light/dark cycles), and were provided a standard mouse diet (Jiangsu Xietong Pharmaceutical Bio-engineering Co., Ltd., Jiangsu, China) and water *ad libitum*. A 1-week adaptation period was followed by 2 days of bedding replacement. The mice were randomly divided into four groups, each consisting of 10 mice: normal, control, Vc, and YF01. The experiment lasted for 29 days (Figure 1). In the first week, all mice received daily gavage treatment. The normal and control groups were gavaged with 0.1 ml/10 g of 0.9% saline. The Vc group received 0.1 ml/10 g of 200 mg/Kg Vitamin C (Vc) solution, as a positive control. The YF01 group received 0.1 ml/10 g of a 1.0 × 10^9^ CFU/ml *P. pentosaceus* YF01 solution. In the second week, the mice underwent a 6-day adaptation training on a treadmill set at a 0° slope with a speed controlled at 10 m/min for 10 min per day. Gavage treatment was administered before each exercise/training session. In the third week, a 2-week endurance training was conducted on a 5° slope for 10 min per training session, with an acceleration of 1 m/min and a maximum speed of 10 m/min. Training was conducted for 6 days/week, with 1 rest day, followed by fasting for 16–24 h after the last gavage. In the fifth week (day 29), the exhaustion test was conducted, with its protocol including treadmill running at 0°, 10 m/min for 15 min; 5°, 10 m/min for 15 min; and 10°, 10 m/min until exhaustion, defined as the mouse being willing to endure electric shocks over 3 s for five times (1 mA current), or staying on the electrical grid for 5 s continuously. The exhaustion time was recorded when the mice reached exhaustion. After exhaustion, blood was immediately collected from the eye socket of the mice, and the liver and muscle tissues of the mice were isolated for further use. The Animal Ethics Committee of Beijing University of Chemical Technology approved all animal experiments.

### 2.7 H&E staining

Approximately half of the right liver lobe and half of the muscle tissues of the mice were excised, rinsed with saline solution, and fixed in a tissue fixative. After the tissues were rehydrated with ethanol, they were soaked in xylene and ethanol for approximately 30 min, embedded in paraffin, sectioned using a microtome, and mounted on glass slides. The cell morphology was analyzed using an optical microscope (BX43F, Olympus Co., Tokyo, Japan) after the cytoplasm was stained with hematoxylin and eosin (H&E) dyes (Pan et al., 2020).

### 2.8 Mice serum index detection

For later use, the mouse serum was centrifuged for 10 min at 4°C and 4,000 rpm, collected, and stored at −80°C. Following the manufacturer’s recommended protocol, appropriate biochemical assay kits were used to determine the serum levels of total antioxidant capacity (T-AOC), catalase (CAT), glutathione (GSH), aspartate aminotransferase (AST), alanine aminotransferase (ALT), total protein (TP), albumin (Alb), glucose (GLU), LA, lactate dehydrogenase (LDH), blood urea nitrogen (BUN), uric acid (UA), and creatinine (CRE).

### 2.9 Real-time quantitative PCR

A SYBR Green assay was performed to determine mRNA expression in the mouse liver and skeletal muscle tissues. Approximately 100 mg of tissues were homogenized. Total RNA was extracted from the tissues by using the TRIzol reagent (ABclonal Technology Co., Ltd., Wuhan, China). Using a spectrophotometer (Allsheng Co., Hangzhou, China), the RNA concentration was measured. RNA was reverse transcribed with Hifair™ II 1st Strand cDNA Synthesis SuperMix for qPCR to obtain cDNA templates. Subsequently, amplification was performed using the StepOnePlus™ Real-Time PCR System (Thermo Fisher Scientific Inc., Waltham, MA, USA) comprising 10 μl SYBR Green PCR Master Mix, 1 μl of each primer, 1 μl of cDNA template, and 7 μl of DEPC. The cycling conditions were set as follows: 95°C for 3 min; 40 cycles of 95°C for 5 s, and 60°C for 30 s. The gene expression levels were determined by calculating the relative expression levels of each gene by using the 2^–ΔΔCT^ method. This calculation included ββ-actin as the internal reference gene and CT representing the cycle threshold. Table 1 lists primer sequences used in this study.

**TABLE 1:** Primer names and sequences.

Gene name	Primer sequence
SOD1	F: 5′-AACCAGTTGTGTTGTCAGGAC-3′ R: 5′-CCACCATGTTTCTTAGAGTGAGG-3′
SOD2	F: 5′-CAGACCTGCCTTACGACTATGG-3′ R: 5′-CTCGGTGGCGTTGAGATTGTT-3′
CAT	F: 5′-GGAGGCGGGAACCCAATAG-3′ R: 5′-GTGTGCCATCTCGTCAGTGAA-3′
MycH I	F: 5′-ACTGTCAACACTAAGAGGGTCA-3′ R: 5′-TTGGATGATTTGATCTTCCAGGG-3′
MycH II a	F: 5′-TAAACGCAAGTGCCATTCCTG-3′ R: 5′-GGGTCCGGGTAATAAGCTGG-3′
MycH II b	F: 5′-CTTTGCTTACGTCAGTCAAGGT-3′ R: 5′-AGCGCCTGTGAGCTTGTAAA-3′
MycH II x	F: 5′-GCGAATCGAGGCTCAGAACAA-3′ R: 5′-GTAGTTCCGCCTTCGGTCTTG-3′
SIRT I	F: 5′-ATGACGCTGTGGCAGATTGTT-3′ R: 5′-CCGCAAGGCGAGCATAGAT-3′
PGC	F: 5′-TATGGAGTGACATAGAGTGTGCT-3′ R: 5′-CCACTTCAATCCACCCAGAAAG-3′
β-actin	F: 5′-ATGGAGCCGGACAGAAAAGC-3′ R: 5′-TGGGAGGTGTCAACATCTTCTT-3′

### 2.10 Gut microbiota analysis

The mouse fecal samples were extracted for genomic DNA using the magnetic bead method and the Soil and Fecal Genomic DNA Extraction Kit (TIANGEN BIOTECH CO., LTD., Beijing, China). Subsequently, 1% agarose gel electrophoresis was performed to assess the purity and concentration of the DNA. An appropriate amount of sample DNA was taken in a centrifuge tube and diluted with sterile water to a concentration of 1 ng/μl. All PCR mixtures were prepared by adding 15 μl of Phusion™ High-Fidelity PCR Master Mix (New England Biolabs), 0.2 μM primers (16S V4 region primers 515F and 806R), and 10 ng of genomic DNA template. The PCR amplification included an initial denaturation at 98°C for 1 min, followed by 30 cycles of denaturation at 98°C for 10 s, annealing at 50°C for 30 s, and extension at 72°C for 30 s, with a final extension at 72°C for 5 min. The PCR products were analyzed by electrophoresis on a 2% agarose gel. Qualified PCR products underwent magnetic bead purification, quantified using enzymatic methods, and pooled in equimolar concentrations based on the PCR product concentration. After thorough mixing, the PCR products were subjected to another round of electrophoresis on a 2% agarose gel. Target bands were purified using a universal DNA purification kit (TIANGEN). Library construction was carried out using the NEBNext^®^ Ultra™ II FS DNA PCR-free Library Prep Kit (New England Biolabs). The constructed libraries were quantified with Qubit and Q-PCR, and after passing quality control, PE 250 sequencing was performed on the NovaSeq 6000 platform.

Split each sample data from the raw data based on the Barcode sequence and PCR amplification primer sequence. After trimming the Barcode and primer sequences, the reads of each sample were assembled using FLASH (Version 1.2.11^1^) (Magoc and Salzberg, 2011), resulting in the assembled sequences as the Raw Tags. The assembled Raw Tags were subjected to rigorous filtering using fastp software (Version 0.23.1) to obtain high-quality Clean Tags (Bokulich et al., 2013). After the aforementioned processing, the Tags obtained need to undergo the removal of chimeric sequences. The Tags sequences are aligned with species annotation databases (Silva database^2^ for 16S/18S, Unite database^3^ for ITS) to detect chimeric sequences and ultimately remove them to obtain the Effective Tags (Edgar et al., 2011).

### 2.11 Data analysis

The averages of serum and tissue indices of each mouse were calculated from three or more parallel experiments. Data were analyzed using IBM SPSS 22 statistical software. Results are expressed as mean ± standard deviation. One-way ANOVA, followed by Duncan’s multiple range test, was used to determine differences between the mean values of each group. Differences with a *p*-value of <0.05 were considered statistically significant.

## 3 Results

### 3.1 Biological morphology of the strain

**FIGURE 2:**
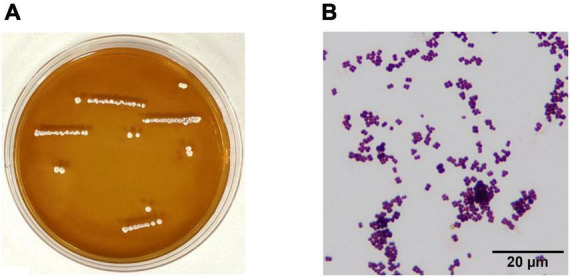
Morphological characteristics of *Pediococcus pentosaceus* YF01. **(A)** intuitive observation, **(B)** gram staining observation.

YF01 exhibited opaque milky-white circular colonies on MRS solid culture medium, with smooth and raised surfaces and neat edges (Figure 2A). Gram staining revealed that YF01 was gram-positive (Figure 2B), and no spore production was noted.

### 3.2 *In vitro* bioactivity testing of the strain

The experimental results (Table 2) demonstrated that the viability of the experimental strain *P. pentosaceus* YF01 in the artificial gastric juice was 90.11% ± 2.44%, and the viabilities in 0.30%, 0.50%, and 1.00% bile salts were 27.22% ± 0.42%, 23.91% ± 0.21%, and 16.16% ± 0.18%, respectively. Additionally, the hydroxyl radical scavenging rate of YF01 was 19.24% ± 0.33%, and 14.73% ± 0.98% for the DPPH radical scavenging rate. Based on the high viability of YF01 in gastric acid and bile salts, the experimental strain was considered to have the potential for good survival in the gastrointestinal tract. The free radical scavenging rates also indicated the potential developability of the functional properties of the experimental strain. Subsequently, animal experiments were conducted for further verification.

**TABLE 2:** *In vitro* activity of *Pediococcus pentosaceus* YF01.

*In vitro* activity	%
Survival rate at pH 3.0	90.11 ± 2.44
Growth efficiency in 0.3% bile salt	27.22 ± 0.42
Growth efficiency in 0.5% bile salt	23.91 ± 0.21
Growth efficiency in 1.0% bile salt	16.16 ± 0.18
Hydroxyl free radical scavenging capacity	19.24 ± 0.33
DPPH scavenging activity	14.73 ± 0.98

### 3.3 Mouse weight and organ index

**FIGURE 3:**
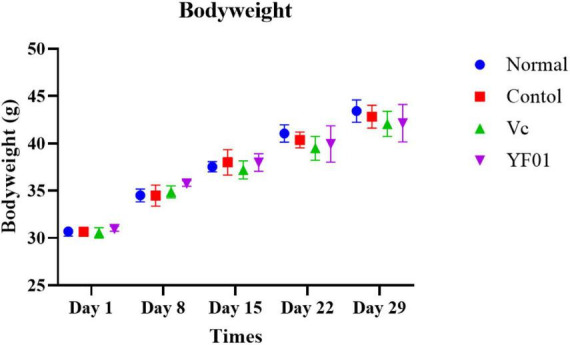
Bodyweight of mice. Normal, gavaged with 0.1 ml/10 g of 0.9% saline; Control, gavaged with 0.1 ml/10 g of 0.9% saline; Vc, gavaged with 0.1 ml/10 g of a 200 mg/Kg Vc solution; YF01, gavaged with 0.1 ml/10 g of a 1.0 × 10^9^ CFU/ml *Pediococcus pentosaceus* YF01 solution.

During the experimental period, the mouse weight changed (Figure 3). From the third week, all exercised mice lost weight at a slower rate than the normal mice. However, no significant difference in weight gain was noted between the groups. The heart, liver, kidneys, spleen, and testis of mice exhibited no significant difference in weight (Table 3). However, the muscle tissue of mice from the Vc and YF01 groups exhibited significant increases. This indicated that YF01 has no toxic effects, and long-term exercise may inhibit abnormal weight gain and enhance muscle development.

**TABLE 3:** Tissue index of mice.^1^

Group	Heart	Liver	Kidney	Spleen	Muscle	Testis
Normal	0.45 ± 0.01^a^	3.94 ± 0.10^ab^	1.27 ± 0.06^b^	0.32 ± 0.01^a^	2.42 ± 0.09^b^	0.60 ± 0.01^a^
Control	0.45 ± 0.01^a^	3.79 ± 0.04^b^	1.27 ± 0.07^b^	0.33 ± 0.02^a^	2.49 ± 0.15^b^	0.54 ± 0.02^b^
Vc	0.44 ± 0.02^a^	3.79 ± 0.09^a^	1.29 ± 0.07^b^	0.33 ± 0.03^a^	2.74 ± 0.43^ab^	0.55 ± 0.02^b^
YF01	0.46 ± 0.03^a^	4.01 ± 0.20^b^	1.41 ± 0.01^a^	0.32 ± 0.04^a^	2.85 ± 0.17^a^	0.54 ± 0.02^b^

^1^Normal, gavaged with 0.1 ml/10 g of 0.9% saline; Control, gavaged with 0.1 ml/10 g of 0.9% saline; Vc, gavaged with 0.1 ml/10 g of a 200 mg/Kg Vc solution; YF01, gavaged with 0.1 ml/10 g of a 1.0 × 10^9^ CFU/ml *Pediococcus pentosaceus* YF01 solution. Means with the different letters (a–b) are significantly different (*p* < 0.05) using Duncan’s multiple range test.

### 3.4 Mouse exhaustion time

The exhaustion time of mice typically reflects their anti-fatigue ability and endurance. The shortest exhaustion time of 2,998.32 ± 139.11 s was noted in the control group, whereas the exhaustion times in the Vc and YF01 groups were 4,139.64 ± 220.44 s and 3,562.92 ± 239.02 s, respectively (Table 4). These results of exhaustion time suggest that the intake of Vc and YF01 can augment the endurance of mice.

**TABLE 4:** Exercise exhaustion time of mice.^1^

Group	Times (s)
Control	2,998.32 ± 139.11^c^
Vc	4,139.64 ± 220.44^a^
YF01	3,562.92 ± 239.02^b^

^1^Normal, gavaged with 0.1 ml/10 g of 0.9% saline; Control, gavaged with 0.1 ml/10 g of 0.9% saline; Vc, gavaged with 0.1 ml/10 g of a 200 mg/Kg Vc solution; YF01, gavaged with 0.1 ml/10 g of a 1.0 × 10^9^ CFU/ml *Pediococcus pentosaceus* YF01 solution. Means with the different letters (a–c) are significantly different (*p* < 0.05) using Duncan’s multiple range test.

### 3.5 Histological analysis of mouse tissues

**FIGURE 4:**
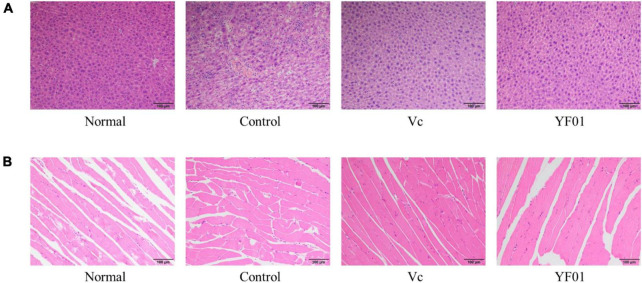
Liver **(A)** and muscle **(B)** morphology of mice. Normal, gavaged with 0.1 ml/10 g of 0.9% saline; Control, gavaged with 0.1 ml/10 g of 0.9% saline; Vc, gavaged with 0.1 ml/10 g of a 200 mg/Kg Vc solution; YF01, gavaged with 0.1 ml/10 g of a 1.0 × 10^9^ CFU/ml *Pediococcus pentosaceus* YF01 solution.

Figure 4 presents the histological sections of mouse liver and muscle tissues. In normal liver tissues, the hepatocyte structure was intact, with clear, large, and round nuclear structures. The hepatic lobule structure was largely intact in the control group, but the hepatocyte arrangement was irregular, with scattered cells exhibiting reduced volume, increased cytoplasm density, concentrated nuclei, and fragmented nuclear membranes. Occasional cell shrinkage and nuclear condensation were observed in the liver tissues of the Vc group. Compared with the normal group, the YF01 group exhibited the most similar liver tissue structure. On visualizing the H&E-stained sections of muscle tissues, we noted that exercise fatigue in the control group mice resulted in muscle inflammation and damage, characterized by changes in muscle fibers, including blurred muscle bundle borders, irregular arrangement of muscle fibers, interstitial diffusion, increased gaps, connective tissue proliferation, and infiltration of inflammatory cells. When supplemented through food, Vc or YF01 helps in improving these conditions by reducing inflammation infiltration and narrowing the gaps between muscle fibers. In conclusion, YF01 exhibited a beneficial reparative effect on the livers and muscles of mice treated with YF01.

### 3.6 Mouse serum levels of oxidative stress markers T-AOC, CAT, and GSH

**FIGURE 5:**
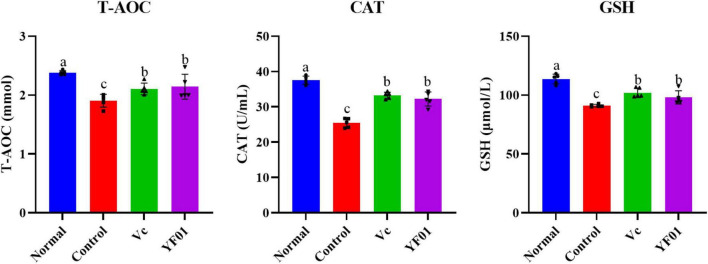
Serum levels of oxidant-related indices of mice. Normal, gavaged with 0.1 ml/10 g of 0.9% saline; Control, gavaged with 0.1 ml/10 g of 0.9% saline; Vc, gavaged with 0.1 ml/10 g of a 200 mg/Kg Vc solution; YF01, gavaged with 0.1 ml/10 g of a 1.0 × 10^9^ CFU/ml *Pediococcus pentosaceus* YF01 solution. Means with the different letters (a–c) above the bars are significantly different (*p* < 0.05) using Duncan’s multiple range test.

According to Figure 5, mouse serum levels of oxidation-related indicators. The control group had the lowest levels of the oxidation indicators T-SOD, CAT, and GSH. Serum levels of T-SOD, CAT, and GSH significantly increased in the Vc and YF01 groups (*p* < 0.05) compared with the control group. Moreover, neither the Vc group nor the YF01 group exhibited significant differences in oxidation-related indicators (*p* > 0.05). The above results indicate that YF01 has a good inhibitory effect on oxidative stress caused by exercise fatigue.

### 3.7 Mouse serum levels of liver-related indicators

**FIGURE 6:**
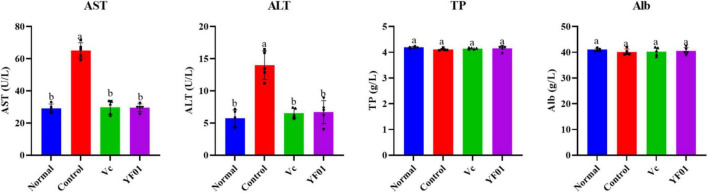
Serum levels of liver function related indices of mice. Normal, gavaged with 0.1 ml/10 g of 0.9% saline; Control, gavaged with 0.1 ml/10 g of 0.9% saline; Vc, gavaged with 0.1 ml/10 g of a 200 mg/Kg Vc solution; YF01, gavaged with 0.1 ml/10 g of a 1.0 × 10^9^ CFU/ml *Pediococcus pentosaceus* YF01 solution. Means with the different letters (a–b) above the bars are significantly different (*p* < 0.05) using Duncan’s multiple range test.

Serum AST and ALT levels were the highest in the control group (Figure 6). Both Vc and YF01 groups exhibited significant reductions in AST and ALT levels compared to the control group (*p* < 0.05), whereas the normal group (*p* > 0.05) exhibited no significant differences. Regarding TP and Alb, no significant differences were noted between the groups (*p* > 0.05). This indicates that exercise fatigue may cause liver damage without affecting protein synthesis or secretion, and YF01 improves exercise fatigue-induced liver damage.

### 3.8 Mouse serum levels of exercise performance-related indicators

**FIGURE 7:**
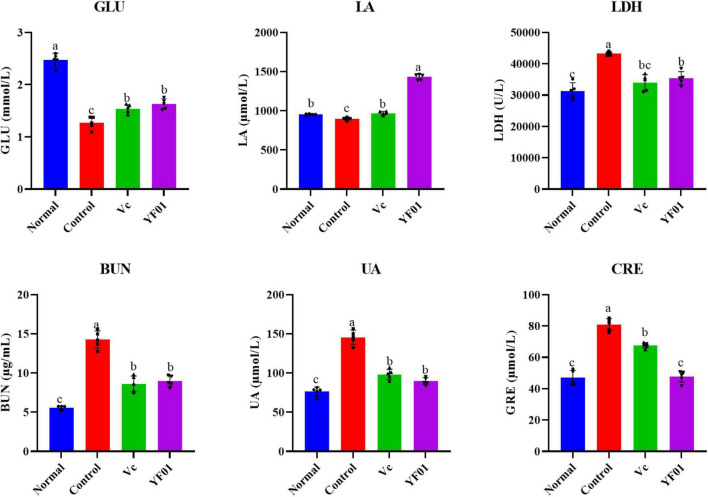
Indices related to physical performance in mice serum. Normal, gavaged with 0.1 ml/10 g of 0.9% saline; Control, gavaged with 0.1 ml/10 g of 0.9% saline; Vc, gavaged with 0.1 ml/10 g of a 200 mg/Kg Vc solution; YF01, gavaged with 0.1 ml/10 g of a 1.0 × 10^9^ CFU/ml *Pediococcus pentosaceus* YF01 solution. Means with the different letters (a–c) above the bars are significantly different (*p* < 0.05) using Duncan’s multiple range test.

As shown in Figure 7, the control group had the lowest GLU and LA levels, whereas it had the highest LDH, BUN, UA, and CRE levels (*p* < 0.05). The intake of Vc and YF01 effectively alleviated the decrease in GLU levels and the increase in LDH, BUN, UA, and CRE levels. The YF01 group exhibited serum GLU, UA, and CRE levels closest to those of the normal group, with no significant differences in LDH and BUN levels compared with the Vc group (*p* > 0.05). A higher LA level was noted in the YF01 group than in the Vc group (*p* < 0.05). Thus, YF01 can effectively inhibit exercise fatigue-induced changes in mouse serum-related performance indicators as well as augment endurance in mice.

### 3.9 Liver tissue oxidation-related gene expression levels in mice

**FIGURE 8:**
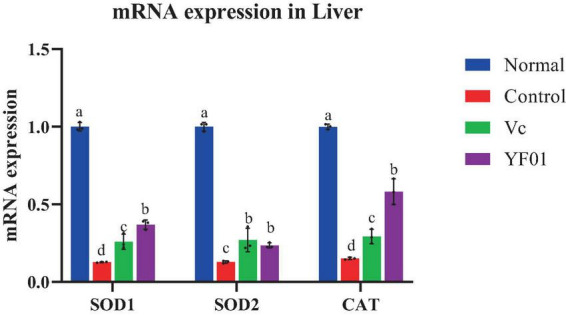
Expression levels of oxidation-related genes mRNA in mice liver tissues. Normal, gavaged with 0.1 ml/10 g of 0.9% saline; Control, gavaged with 0.1 ml/10 g of 0.9% saline; Vc, gavaged with 0.1 ml/10 g of a 200 mg/Kg Vc solution; YF01, gavaged with 0.1 ml/10 g of a 1.0 × 10^9^ CFU/ml *Pediococcus pentosaceus* YF01 solution. Means with the different letters (a–d) above the bars are significantly different (*p* < 0.05) using Duncan’s multiple range test.

The normal group expressed the highest SOD1, SOD2, and CAT levels, whereas the control group expressed the lowest levels (*p* < 0.05, Figure 8). The YF01 mice had similar expression levels of SOD1 and CAT as the normal mice, with no significant differences from the Vc mice (*p* < 0.05). Vc and YF01 groups had similar SOD2 expression levels (*p* > 0.05). The inhibitory effect of YF01 on exercise-induced oxidative damage in the mouse liver tissue was significant.

### 3.10 Muscle mRNA expression levels of genes related to oxidation and exercise performance

**FIGURE 9:**
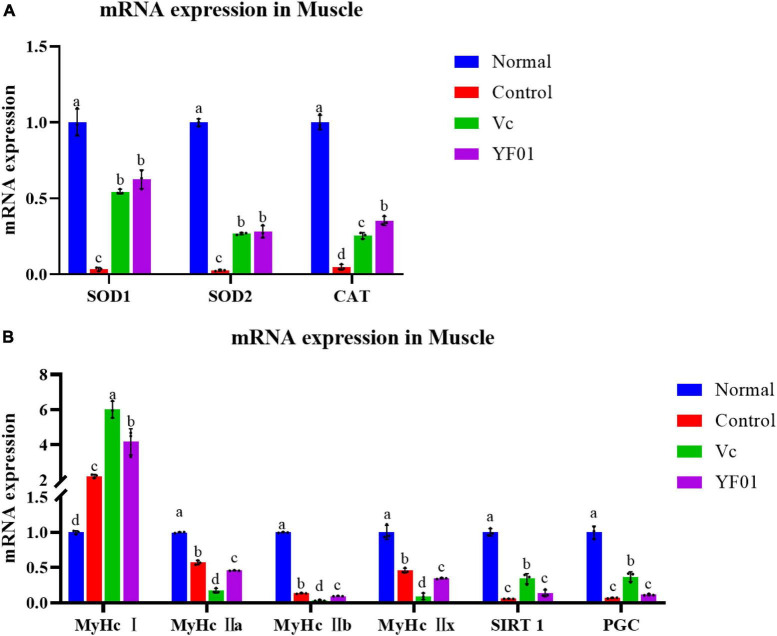
Expression levels of genes related to oxidation **(A)** and physical performance **(B)** in mice muscle tissues. Normal, gavaged with 0.1 ml/10 g of 0.9% saline; Control, gavaged with 0.1 ml/10 g of 0.9% saline; Vc, gavaged with 0.1 ml/10 g of a 200 mg/Kg Vc solution; YF01, gavaged with 0.1 ml/10 g of a 1.0 × 10^9^ CFU/ml *Pediococcus pentosaceus* YF01 solution. Means with the different letters (a–d) above the bars are significantly different (*p* < 0.05) using Duncan’s multiple range test.

Figure 9A presents the mRNA expression levels of oxidation-related genes in the mouse muscle tissue. Similar to the mouse liver tissue, SOD1, SOD2, and CAT expression was the highest in the normal group, whereas it was the lowest in the control group (*p* < 0.05). In the YF01 group, SOD1, SOD2, and CAT expression levels were 18, 10, and 7 times higher than those in the control group. Figure 9B shows the mRNA expression levels for genes related to exercise performance. The normal group exhibited the highest levels of MyHc IIa, MyHc IIb, MyHc IIx, SIRT1, and PGC, with the YF01 group ranking below the control group and the Vc group showing the lowest levels. In contrast, the mRNA expression level of MyHc I is opposite. These results indicate that dietary supplementation with Vc and YF01 can effectively prevent oxidative damage and contribute to improving exercise performance in mice.

### 3.11 Effects of exercise and *P. pentosaceus* YF01 on intestinal microbial communities

**FIGURE 10:**
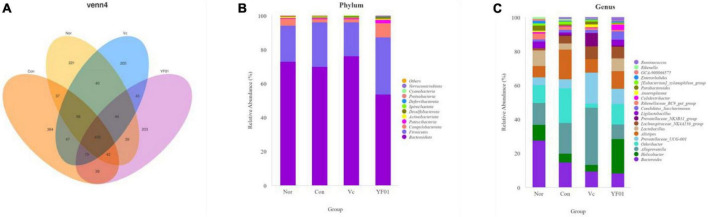
Venn analysis among each group and comparison of dominant phylum and genus in different groups. **(A)** Venn diagrams of bacterial OTUs. **(B)** At the phylum level, the horizontal coordinate represents the group and the vertical coordinate represents the percent of community abundance. **(C)** At the genus level.

By sequencing, we evaluated the effects of exercise and *P. pentosaceus* YF01 on the intestinal microbial community of mice. The results (Figures 10A–C) showed that there were differences in the gut microbiota between the four groups, with all four groups having shared and unique OTU, with 900 OTUs in the Normal group, 1085 OTUs in the Control group, and 935 OTUs in the Vc group, the YF01 group has 898 OTUs, and different samples have 409 common OTUs. Subsequently, comparisons were made at the phylum and genus levels, and it was found that the relative abundance of *Firmicutes* in the YF01 group was higher at the phylum level; at the genus level, the relative abundance of *Bacteroides* was reduced in all exercise groups (Con, Vc, and YF01 group), and the relative abundance of *Helicobacter*, *Saccharimonas*, and *Colidextribacter* was higher in the YF01 group, while the relative abundance of *Alistipes* was higher in the Con group and YF01 group.

**FIGURE 11:**
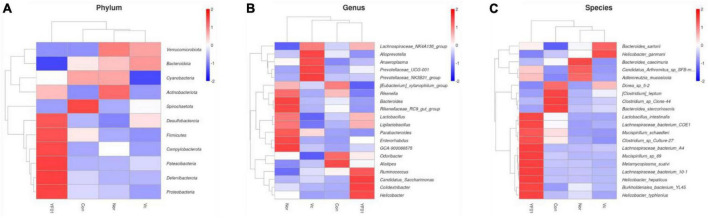
A relative abundance heat map showing significant inter-group differences at different levels. **(A)** At the phylum level. **(B)** At the genus level. **(C)** At the species level.

In addition, the relative abundance heat map results (Figures 11A–C) show that at the phylum level (Figure 11A), Desulfobacterota, Firmicutes, Patescibacteria, and Deferribacterota are more abundant in the YF01 group than in other groups. At the genus level (Figure 11B), the contents of *Bacteroides*, *Rikenellaceae*, *Parabacteroides*, and *Enterorhabdus* as well as GCA-900066575 from the Lachnospiraceae family were more in the Normal group than in the exercise group; *Alloprevotella*, *Anaeroplasma*, and *Prevotellaceae* were most abundant in the VC group; *Ruminococcus*, *Candidatus_Saccharimonas*, *Colidextribacter*, and *Helicobacter* were most abundant in the YF01 group; and *Odoribacter* and *Alistipes* were most abundant in the Con group. At the species level (Figure 11C), *Lactobacillus*, *Lachnospiraceae*, and *Burkholderiales bacterium* were most abundant in the YF01 group.

### 3.12 Principal component analysis and alpha diversity analysis

**FIGURE 12:**
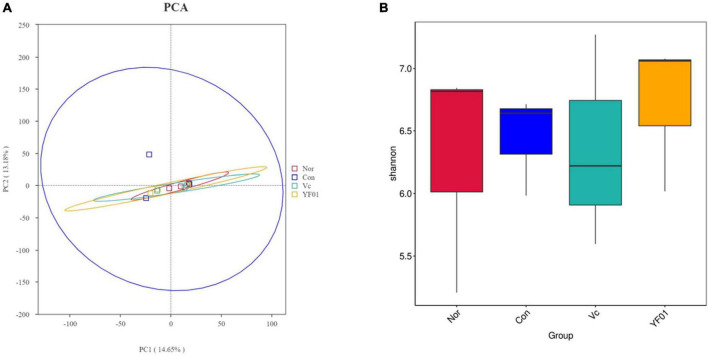
Principal component analysis and alpha diversity analysis. **(A)** Principal component analysis. **(B)** Shannon diversity index.

Principal component analysis (PCA) analysis results (Figure 12A) showed that no significant differences were found between the groups, but it was found that the intestinal microbial diversity increased in the exercise group, among which the microbial community composition of the Vc group and the YF01 group was the most similar. In addition, the shannon index (Figure 12B) reflects that the YF01 group has the highest community diversity and a relatively even distribution of species.

### 3.13 Differential microbial screening

**FIGURE 13:**
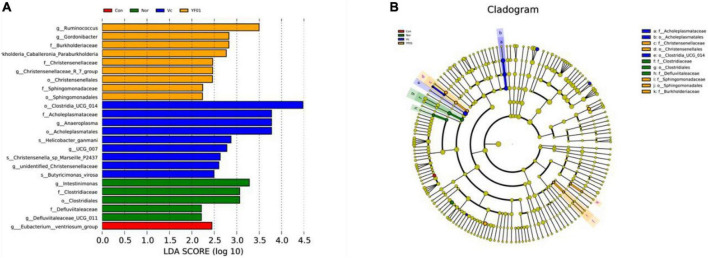
Linear discriminant analysis Effect Size (LEfSe) of different groups, LDA = 2. **(A)** Bar plot of LDA scores distribution. **(B)** Phylogenetic tree.

Linear discriminant analysis Effect Size (LEfSe) analysis (linear discriminant analysis effect size method) was used to determine the characteristic microorganisms of each group, and the relationship between different microbial groups from the phylum to the species level (LDA = 2) was displayed in a cladogram (Figures 13A, B). The results showed that in the control group, the relative abundance of *Eubacterium* was higher; in the normal group, the relative abundance of *Intestinimonas*, *Clostridiaceae*, *Clostridiales*, and *Defluviitaleaceae* was higher. In the VC group, relative abundance of *Clostridia*, *Acholeplasmataceae*, *Anaeroplasma*, *Acholeplasmatales*, *Helicobacter*, UCG 007, *Christensenella* sp. *Marseille*, unidentified *Christensenellaceae*, and *Butyricimonas virosa* was higher; in the YF01 group, the relative abundance of *Ruminococcus*, *Gordonibacter*, Burkholderiaceae, *kholderia Caballeronia Paraburkholderia*, *Christensenellaceae*, *Christensenellales*, *Sphingomonadaceae*, and *Sphingomonadales* is higher. These bacteria with higher relative abundance can be used as biomarkers for further research. Among the four groups, there were 11 OTUs with significant differences at different levels from phylum to species.

## 4 Discussion

Exercise-induced fatigue is a symptom commonly associated with sports and physical activities. It can lead to adverse effects such as muscle pain and physical exhaustion (Wan et al., 2017). Oxidative stress, a physiological response characterized by excessive ROS generation within cells, plays a crucial role in exercise-induced fatigue (Moir et al., 2023). Oxidative stress leads to cellular oxidative damage. High-intensity exercises increase the body’s oxidative stress levels, resulting in muscle fatigue and damage (Debold, 2015). Probiotics are beneficial bacteria believed to protect against oxidative stress (Lee and Kang, 2022). Moreover, probiotics can help the body to recover from oxidative stress and promote muscle repair. Therefore, supplementing probiotics may help reduce exercise-induced fatigue and improve exercise performance (Jager et al., 2019). In this study, *P. pentosaceus* YF01 exhibited good *in vitro* resistance and effectively scavenged oxygen free radicals, thereby reducing exercise-induced damage due to free radicals and enhancing exercise performance.

A probiotic’s survival rate in the artificial gastric fluid and the presence of different bile salt concentrations is a crucial indicator of its ability to colonize the gastrointestinal tract (Bezkorovainy, 2001). Additionally, the *in vitro* antioxidant capacity of probiotics represents their *in vivo* antioxidant capacity. Probiotics with strong *in vitro* antioxidant capacity also exhibit good abilities to scavenge oxygen free radicals *in vivo* (Noureen et al., 2019). In the present study, the YF01 strain demonstrated excellent *in vitro* resistance and a good capacity to scavenge oxygen free radicals, which lays the foundation for its antioxidant mechanisms *in vivo*.

Exercise, particularly high-intensity and prolonged exercise, increases metabolism, which increases the demand for oxygen and oxygen-free radical-induced oxidative stress reactions (Kawamura and Muraoka, 2018). Oxygen free radicals damage nucleic acids, proteins, and lipids, thereby triggering a series of physiological responses including liver and muscle damage (Muriel, 2009; Kıran et al., 2023). The liver, a crucial metabolic organ, is susceptible to oxidative stress (Cichoz-Lach and Michalak, 2014). During exercise, the liver performs the task of clearing metabolic waste and toxins, thereby making the liver cells vulnerable to oxygen-free radical-induced oxidative stress and causing liver damage (Muriel, 2009). An impaired liver function can affect metabolite clearance and synthesis, thereby influencing the body’s physiological functions (Yan et al., 2014). Muscles are executors of exercise, and muscle tissue is also susceptible to the effects of oxidative stress that occurs during exercise (Kozakowska et al., 2015). Oxidative stress can cause damage, such as lipid peroxidation of muscle cell membranes and protein oxidation, impairing the muscle structure and function, which ultimately results in muscle fatigue and damage (Steinbacher and Eckl, 2015). Additionally, oxidative stress may trigger inflammatory reactions in the muscle, exacerbating muscle damage and repair processes (Kozakowska et al., 2015). In an exercise-induced fatigue model, mouse livers suffered oxidative damage, whereas probiotics alleviated this phenomenon (Liu et al., 2021). This study also yielded similar results, the liver and muscle tissues of mice suffered inflammatory damage after exercise, and YF01 effectively alleviated these phenomena, which indicated that YF01 suppressed the liver and muscle damage caused by exercise-induced oxidative stress.

The body has several antioxidant defense systems that help cope with oxidative stress. These include T-AOC, SOD1, SOD2, GSH, and CAT, among others (Surai et al., 2019). Using T-AOC, a key indicator of the antioxidant system, the overall body’s ability to respond to oxidative stress can be assessed (Huang et al., 2022). Cells use GSH as an antioxidant molecule that neutralizes ROS and participates in redox reactions (Lushchak, 2012). SOD1, SOD2, and CAT are vital antioxidant enzymes. SOD1 and SOD2 can eliminate intracellular superoxide radicals, while CAT can degrade harmful oxidants such as hydrogen peroxide, thereby reducing the oxygen-free radical-induced damage (Shields et al., 2021). These antioxidant enzymes play crucial roles in exercise, protecting the cells from oxidative damage and maintaining the oxidative balance within the cells. In this study, exercise-induced fatigue in mice increased the generation of free radicals in the serum, and liver and muscle tissues, and a series of oxidative stress reactions were triggered. *P. pentosaceus* YF01 boosts antioxidant enzyme activity in mice, thereby improving their ability to eliminate free radicals and defend against lipid peroxidation.

Time to exhaustion is a notable indicator for assessing exercise-induced fatigue. It reflects the endurance and persistence of muscles during fatigue (Pageaux and Lepers, 2016). Various factors, including muscle fiber types, expression of exercise-related proteins, and regulation, influence this indicator. In general, a longer time to exhaustion indicates greater endurance. The impact of exercise on metabolic products in the body has always interested researchers, including blood biochemical indicators such as GLU, LA, LDH, BUN, UA, and CRE. These indicators may change during exercise, thereby indicating the body’s regulation of energy and metabolic products during physical activity. GLU is the main energy source in the human body. During exercise, the liver releases glycogen and transports GLU to muscles for energy supply. At the same time, the demand for GLU by muscle cells increases, and the GLU concentration possibly increases to meet the muscle’s energy metabolism needs (Richter and Hargreaves, 2013). On the other hand, LA, a product of glycolysis, accumulates rapidly during high-intensity exercise, thereby increasing the LA concentration. An increase in the lactate threshold indicates augmented endurance (Ghosh, 2004). LDH is involved in the release of lactate metabolism enzymes, which is crucial for lactate production and elimination (Gupta, 2022). BUN indicates the formation of the metabolic product urea nitrogen, which increases during exercise. It is chiefly influenced by amino acid metabolism in the body (Sokal et al., 2013). UA and CRE are metabolic waste products, and various factors affect their concentrations, including muscle metabolism, water balance, and kidney function. During exercise, UA and CRE concentrations may change because of increased muscle metabolism and water loss, reflecting the effect of muscle metabolism and exercise load on the kidneys (Golino et al., 2021; Gherghina et al., 2022). The study findings indicate that *P. pentosaceus* YF01 administration significantly increases the time to exhaustion in running mice and effectively alleviates the effects of exercise-induced fatigue on various biochemical markers, including GLU, LA, LDH, BUN, UA, and CRE. This reflects the positive role of YF01 in regulating the balance of energy and metabolic products during physical activity in mice.

The bulk of muscles is composed of fast-twitch fibers (Type II) and slow-twitch fibers (Type I). As slow-twitch fibers contain heavy myosin chains (MyHc I), they exhibit endurance qualities, which provides them the ability to offer a prolonged energy supply. Fast-twitch fibers are further divided into MyHc IIa, MyHc IIb, and MyHc IIx, and each of them has a different contraction speed and endurance performance (Talbot and Maves, 2016). Sirtuin 1 (SIRT1) and peroxisome proliferator-activated receptor gamma coactivator (PGC), as crucial regulatory proteins in cells, are associated with the regulation of cellular energy metabolism and time to exhaustion (Canto and Auwerx, 2009). SIRT1 is involved in regulating the cellular metabolic balance and oxidative stress response. It thus is crucial for the cell’s adaptive capacity to exercise (Radak et al., 2020). PGC is involved in regulating muscle mitochondrial biogenesis and exercise performance, and thus, it influences time to exhaustion and muscle endurance (Lira et al., 2010). During exercise-induced fatigue, muscle fibers undergo continuous intense exercise loading, which results in the depletion of energy reserves, LA accumulation, and oxidative stress. This negative impact possibly interferes with the expression and activity of MyHc I, MyHc IIa, MyHc IIb, and MyHc IIx in the muscle and disrupts the regulatory functions of SIRT1 and PGC, thereby influencing muscle endurance and exercise performance. The study findings indicate that *P. pentosaceus* YF01 by regulating muscle fibers, this probiotic enhances the body’s overall exercise capacity, particularly in terms of endurance and recovery rate.

Intestinal microbiota plays a crucial role in human health, including influencing exercise capacity through various pathways. Intestinal flora can directly affect nerve transmission and regulate muscle movement and coordination by synthesizing and decomposing neurotransmitters such as dopamine and glutamate (Wu et al., 2020). Research has also found that the composition and interactions of intestinal flora may affect muscle quality, function, and energy metabolism by altering the gut microbiota, known as the gut-muscle axis (Ticinesi et al., 2019). Additionally, intestinal microbiota responds to endurance exercise by adaptively regulating various biological functions, including energy metabolism, inflammatory response, stress resistance, and oxidative stress, thereby playing a role in exercise regulation (Li et al., 2022). Our study revealed that the intake of *P. pentosaceus* YF01 increased the abundance of beneficial bacterial groups such as *Firmicutes*, *Lactobacillus*, *Lachnospiraceae*, and *B. bacterium* Y45 in the mouse intestines, which may be a potential reason for improving the mice’s exercise performance.

## 5 Conclusion

In the present study, a mouse exercise-induced fatigue model was established. Using this model, we demonstrated that *P. pentosaceus* YF01 regulates oxidative stress in mice so as to enhance their antioxidant capacity, thus preventing damage due to exercise-induced fatigue and augmenting the exercising capacity. According to the experimental results, YF01 exhibited good *in vitro* resistance; reduced liver and muscle damage; prolonged the time to exhaustion in running mice; and increased serum T-AOC, CAT, GSH, GLU, and LA levels in mice and liver/muscle mRNA expression of SOD1, SOD2, and CAT. In addition, YF01 upregulated MyHc I, SIRT1, and PGC mRNA expression levels in the muscle tissue, whereas decreased serum AST, ALT, LDH, BUN, UA, and CRE levels and downregulated MyHc IIa, MyHc IIb, and MyHc IIx mRNA expression levels. And YF01 promoted the abundance of beneficial bacteria such as *Lactobacillus* and *Lachnospiraceae* in the gut microbiota of mice. In summary, this study demonstrated that *P. pentosaceus* YF01 can effectively mitigate exercise-induced fatigue through the by regulating oxidative stress response and muscle fiber expression. We also elucidated the action mechanism of YF01, thereby providing a basis for the mitigation of exercise-induced fatigue and enhancement of exercise performance by using food-derived antioxidants.

## Data availability statement

The original contributions presented in this study are included in the article/Supplementary material, further inquiries can be directed to the corresponding author.

## Ethics statement

The animal study was approved by the Beijing University of Chemical Technology. The study was conducted in accordance with the local legislation and institutional requirements.

## Author contributions

XY: Writing – original draft. YW: Writing – original draft. YY: Writing – review & editing.

## Footnotes

1
http://ccb.jhu.edu/software/FLASH/

2
https://www.arb-silva.de/

3
https://unite.ut.ee/
