# Supplementary figures and images for: Impact of Pediococcus pentosaceus YF01 on the exercise capacity of mice through the regulation of oxidative stress and alteration of gut microbiota
Source: Front Microbiol. 2024 Jun 26;15:1421209. doi: 10.3389/fmicb.2024.1421209 (PMC11233450; doi:10.3389/fmicb.2024.1421209)

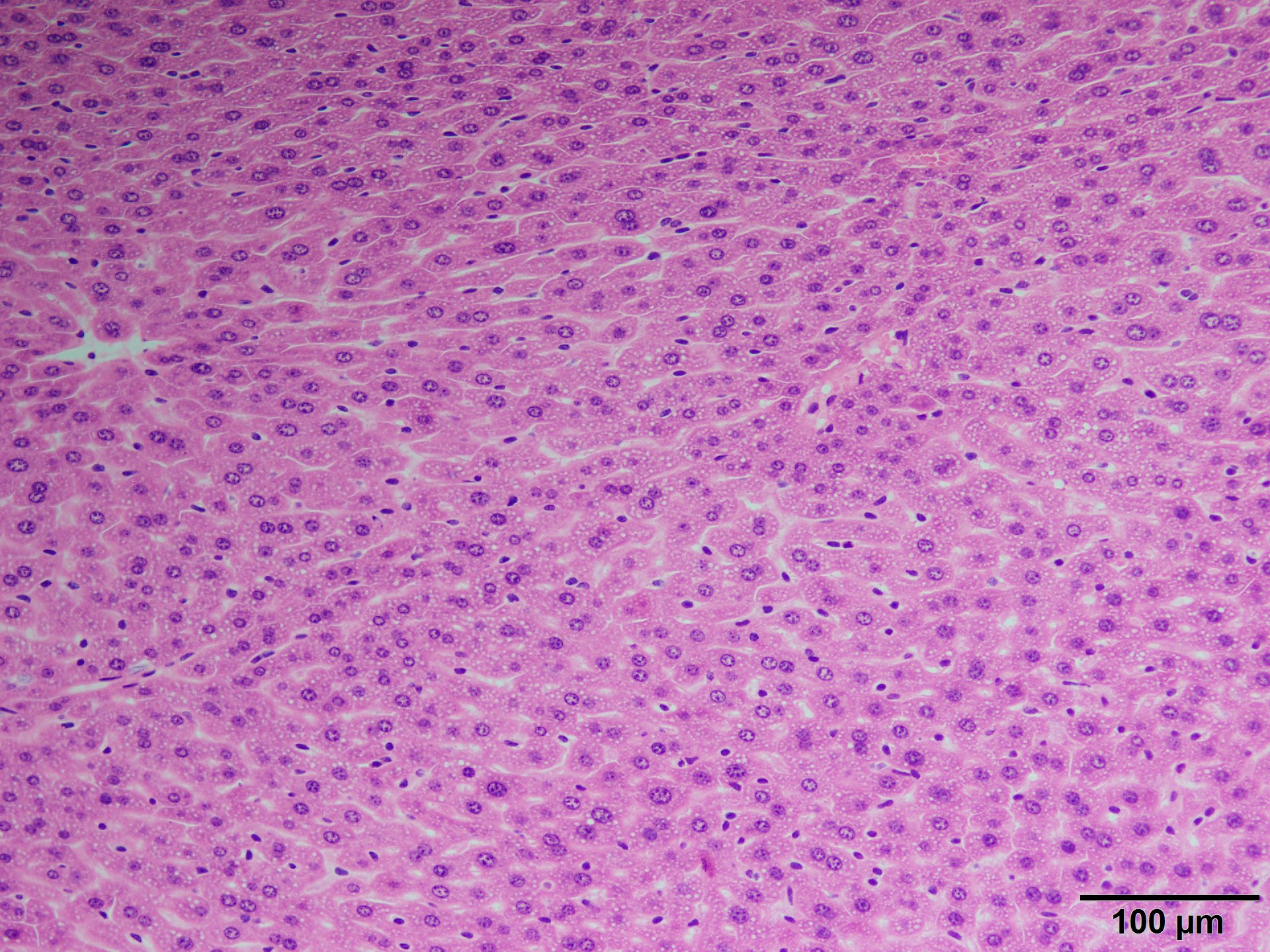

Supplement: Supplementary file 1 [file Data_Sheet_1.ZIP › Raw Data/H&E of Liver/Control/2-1-3.jpg]

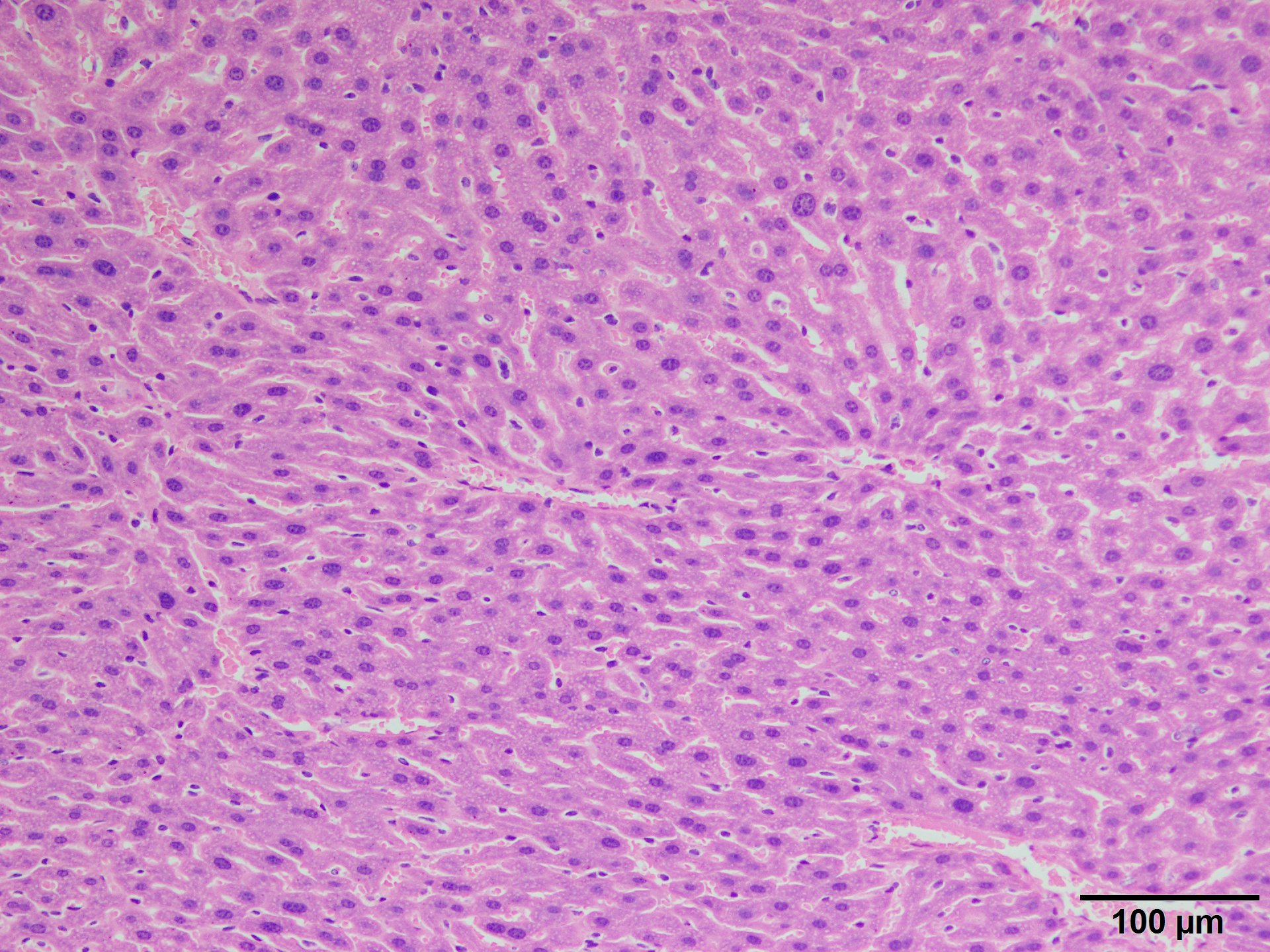

Supplement: Supplementary file 1 [file Data_Sheet_1.ZIP › Raw Data/H&E of Liver/Control/2-18-5.jpg]

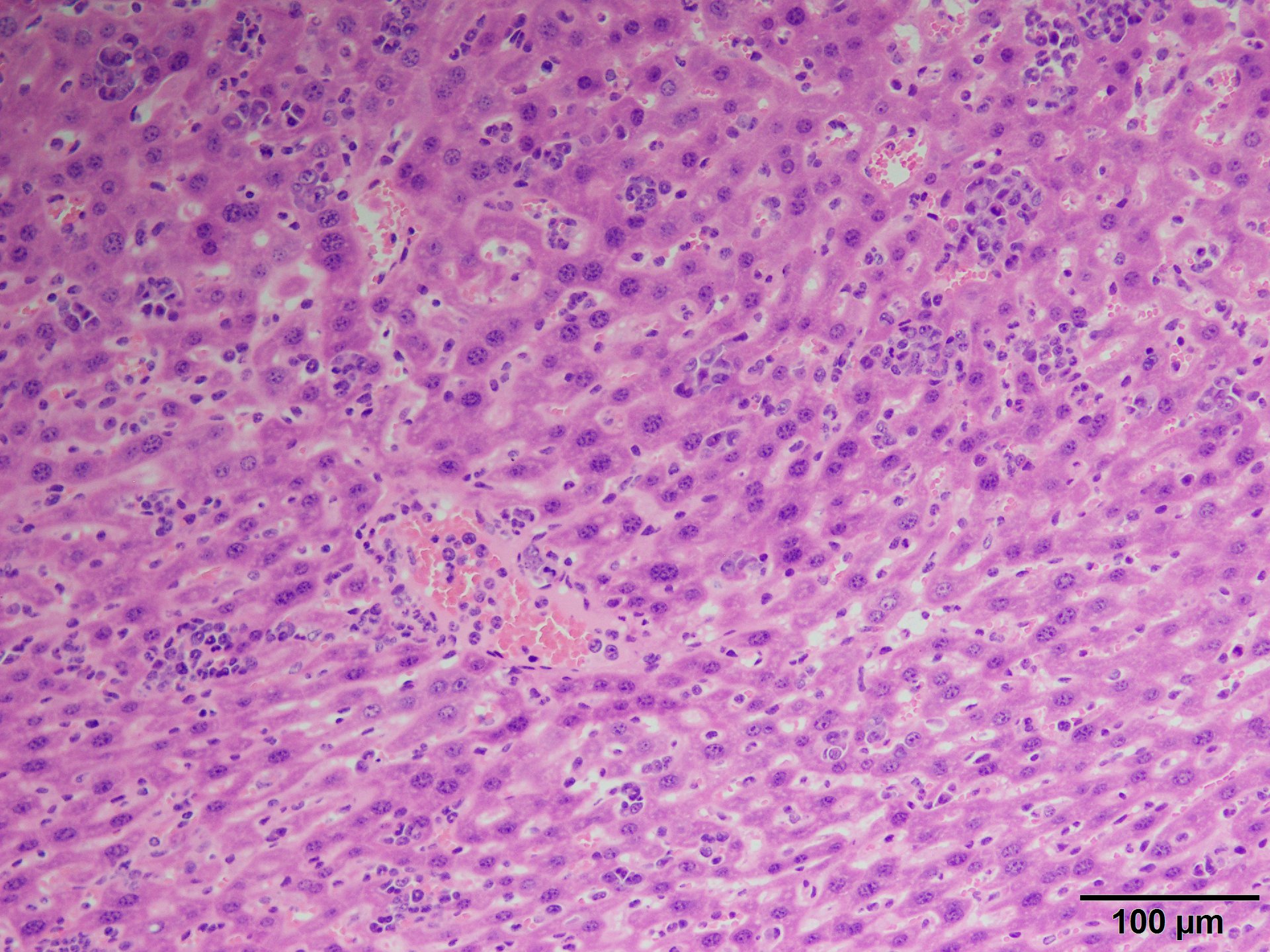

Supplement: Supplementary file 1 [file Data_Sheet_1.ZIP › Raw Data/H&E of Liver/Control/select-2-22-2.jpg]

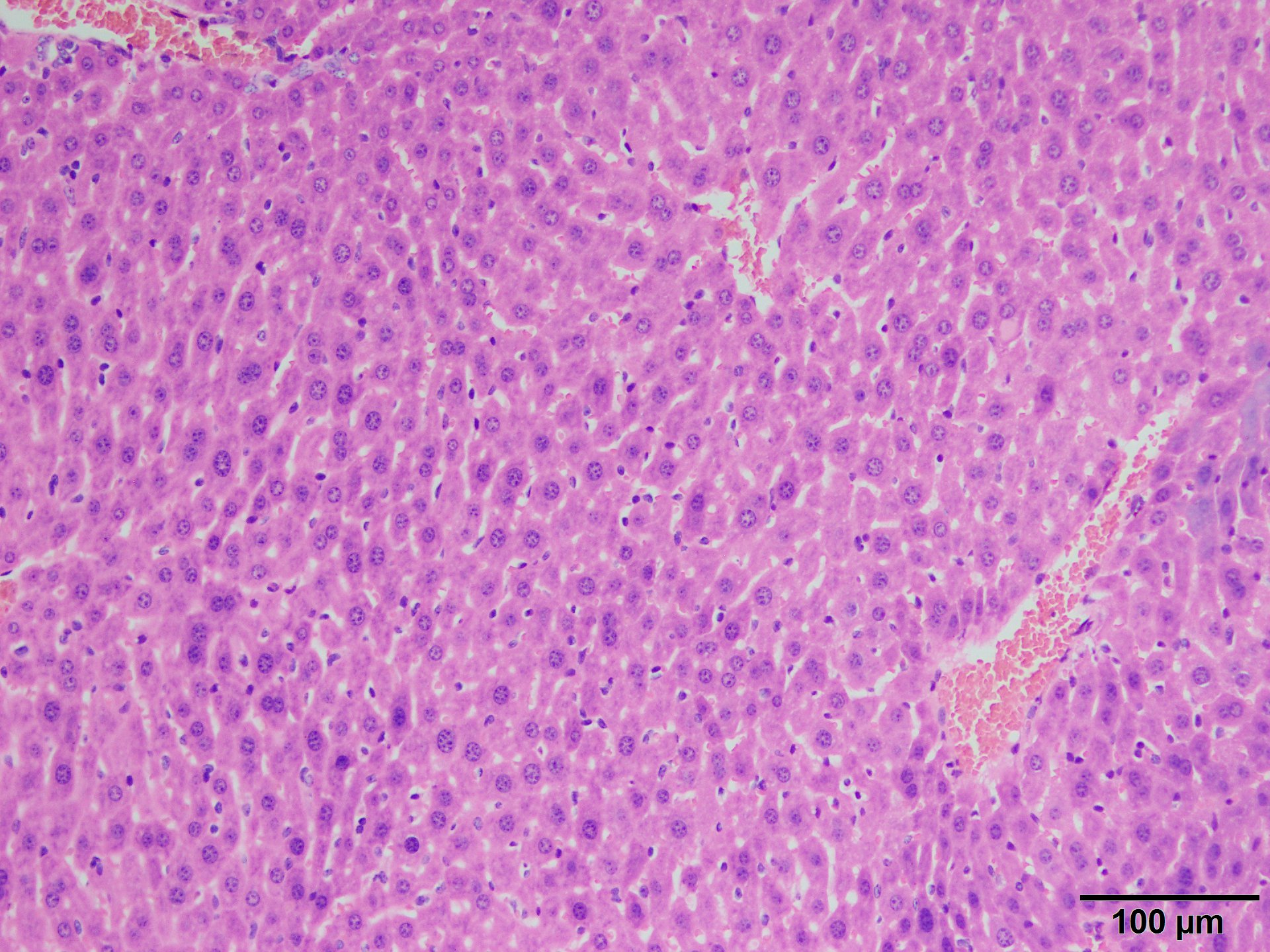

Supplement: Supplementary file 1 [file Data_Sheet_1.ZIP › Raw Data/H&E of Liver/Normal/1-13-1.jpg]

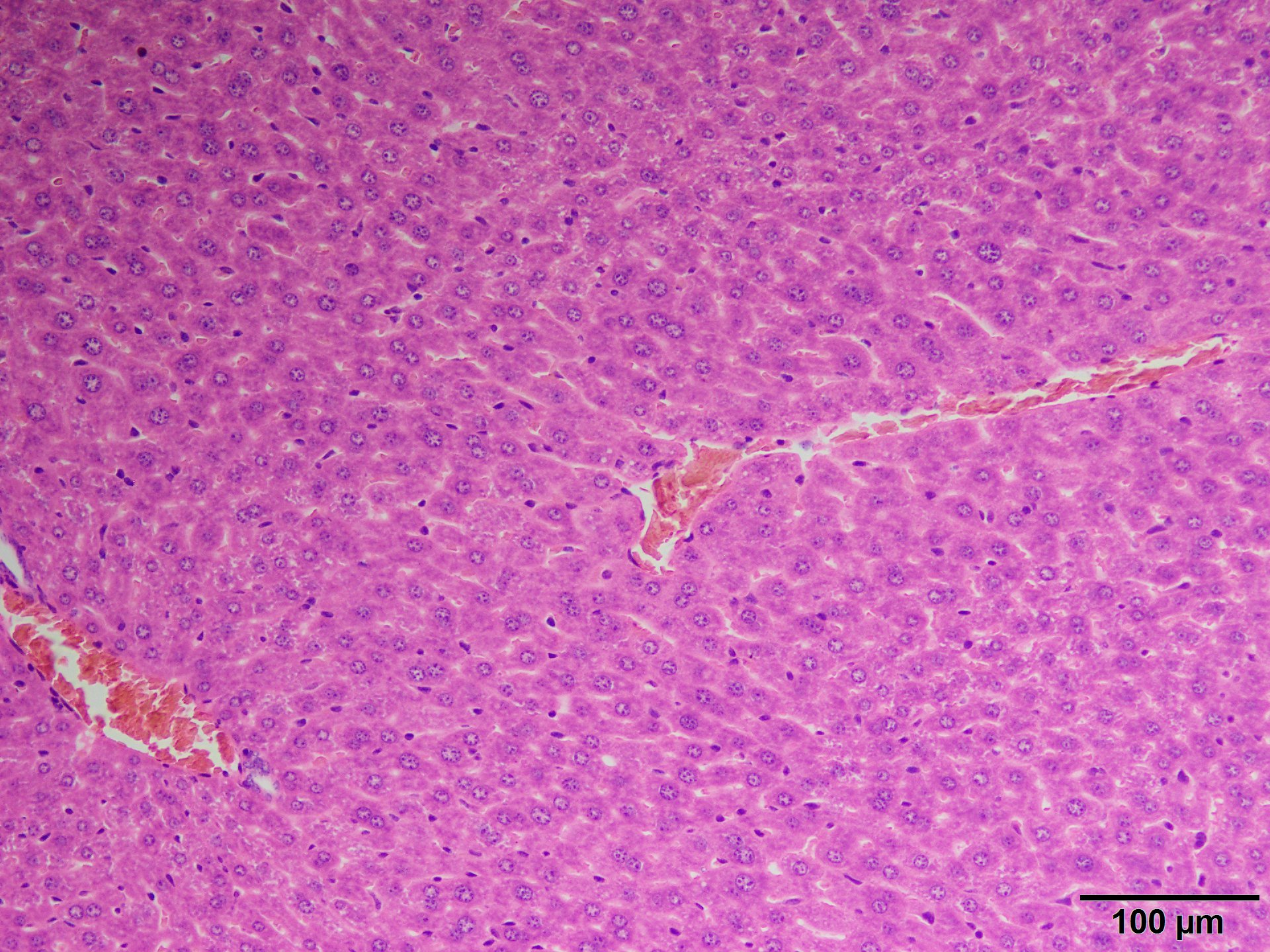

Supplement: Supplementary file 1 [file Data_Sheet_1.ZIP › Raw Data/H&E of Liver/Normal/1-29-4.jpg]

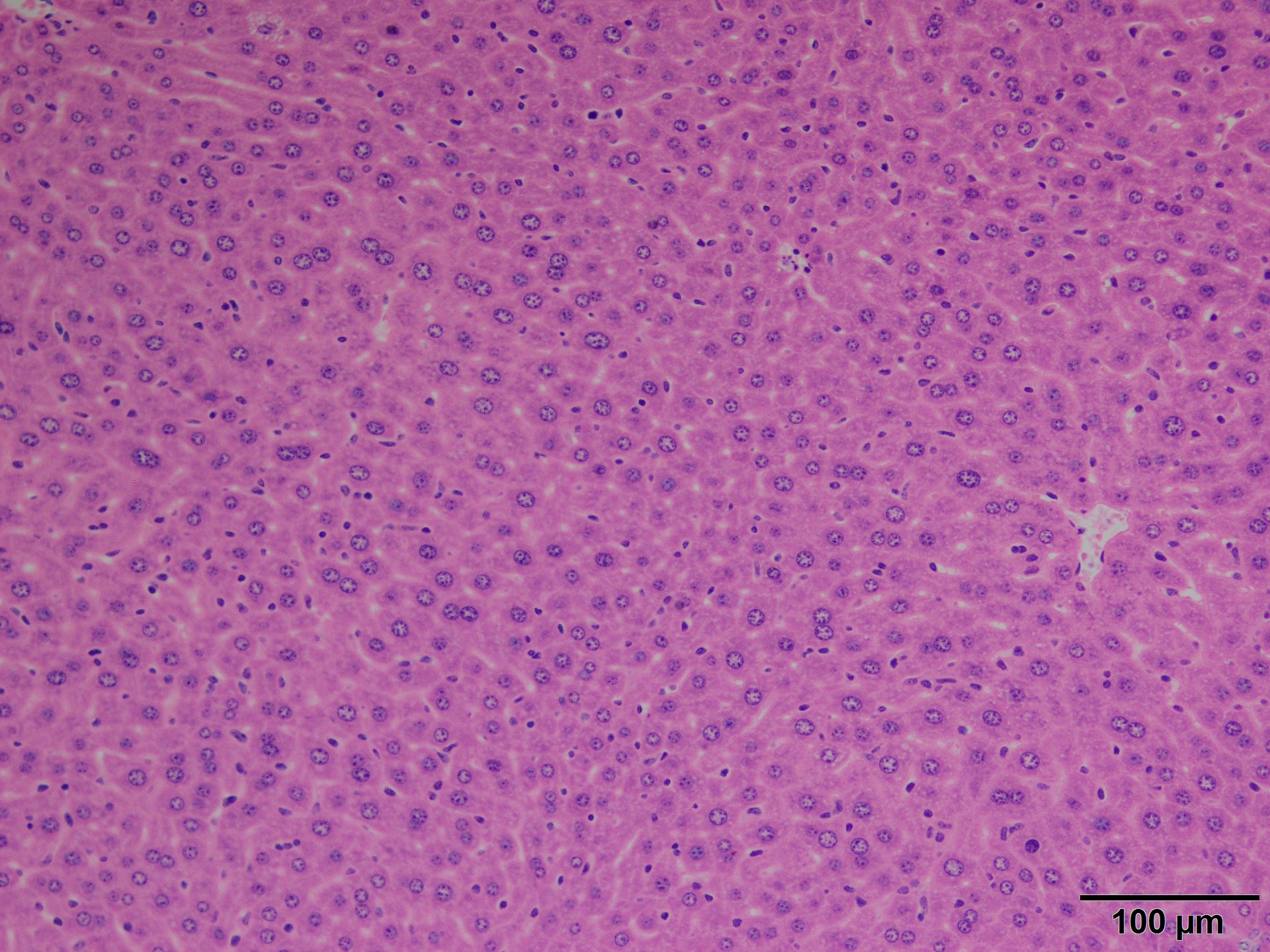

Supplement: Supplementary file 1 [file Data_Sheet_1.ZIP › Raw Data/H&E of Liver/Normal/select-1-3-2.jpg]

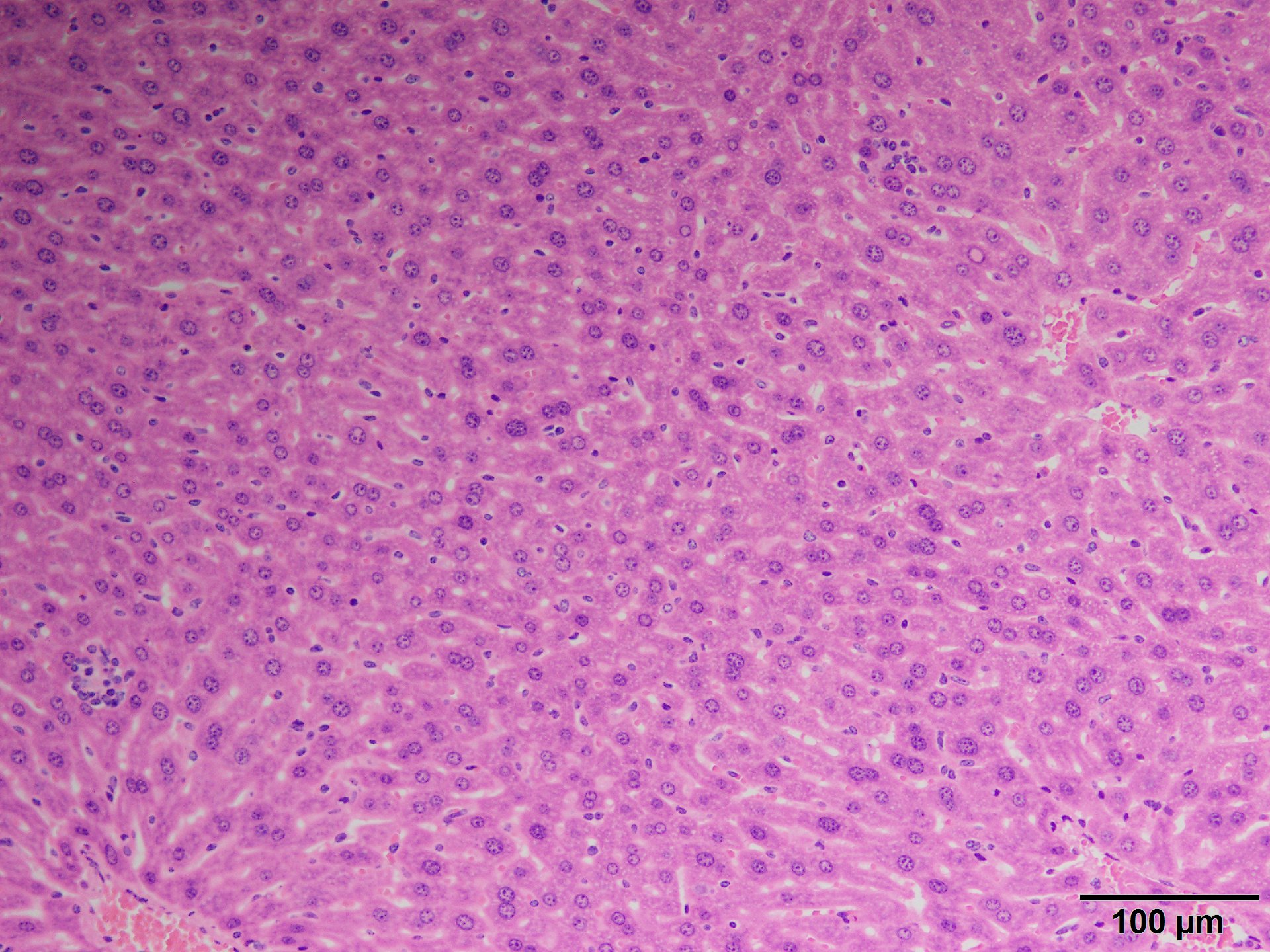

Supplement: Supplementary file 1 [file Data_Sheet_1.ZIP › Raw Data/H&E of Liver/Vc/3-1-2.jpg]

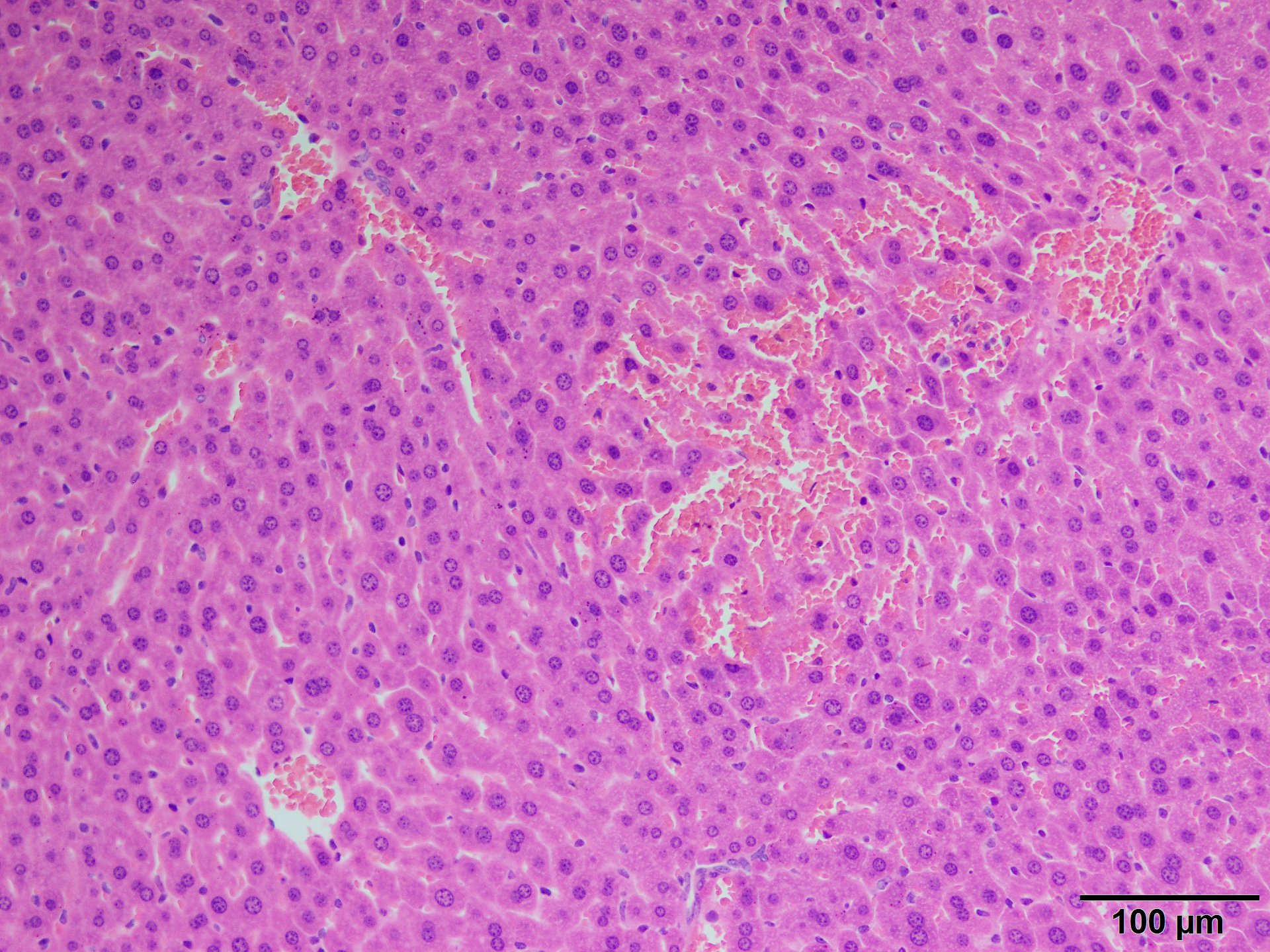

Supplement: Supplementary file 1 [file Data_Sheet_1.ZIP › Raw Data/H&E of Liver/Vc/3-27-5.jpg]

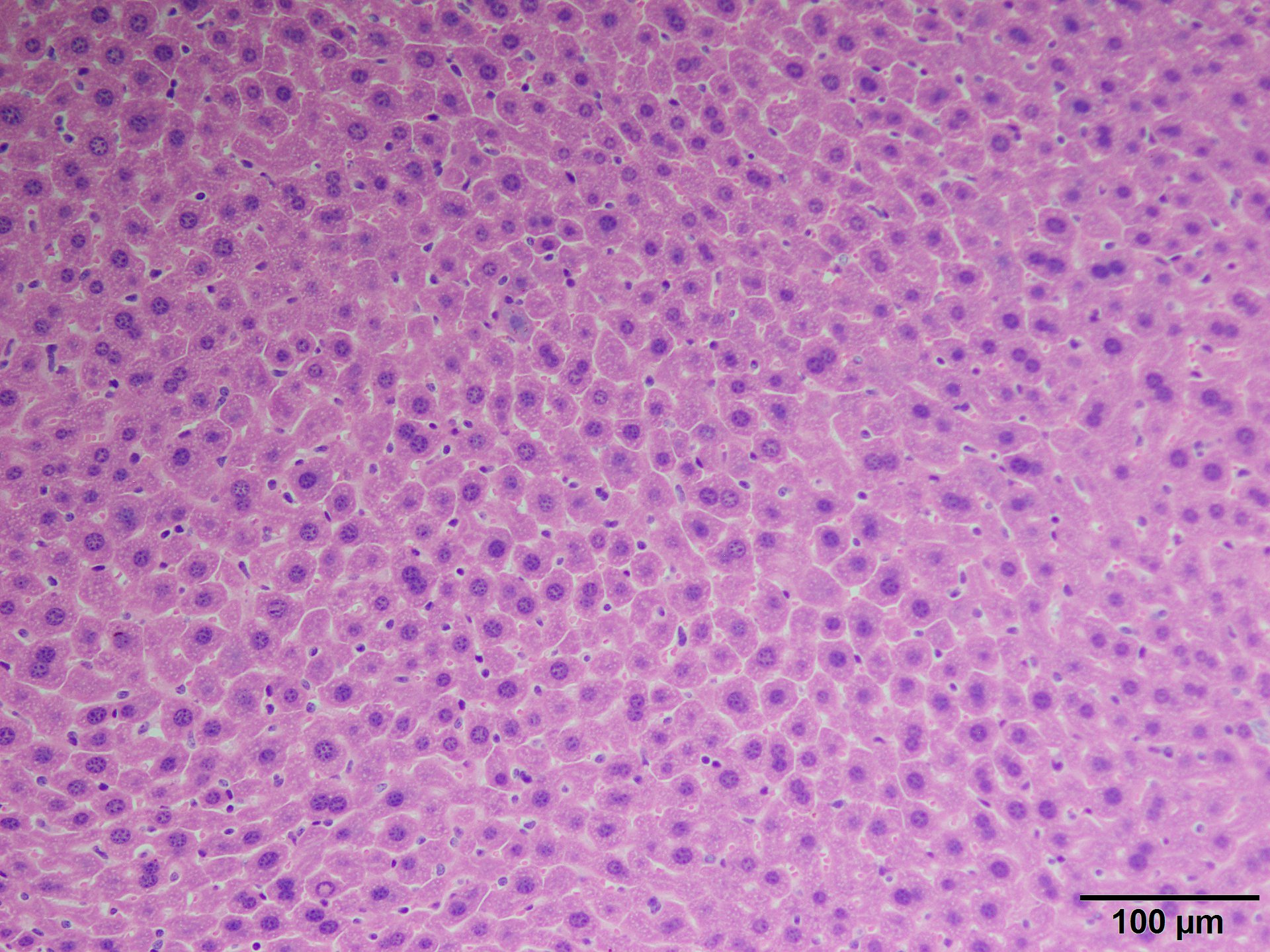

Supplement: Supplementary file 1 [file Data_Sheet_1.ZIP › Raw Data/H&E of Liver/Vc/select-3-19-4.jpg]

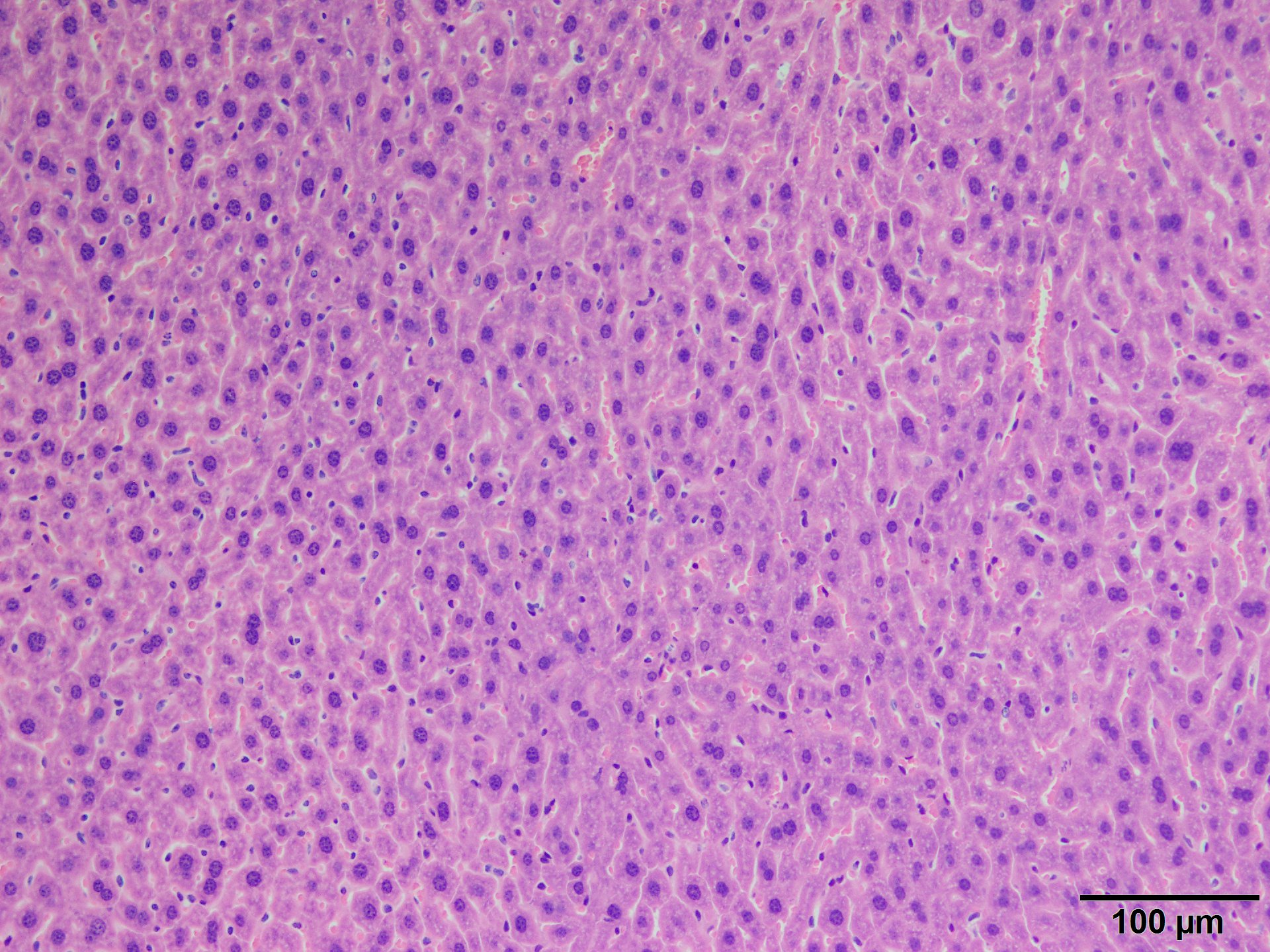

Supplement: Supplementary file 1 [file Data_Sheet_1.ZIP › Raw Data/H&E of Liver/YF01/6-10-2.jpg]

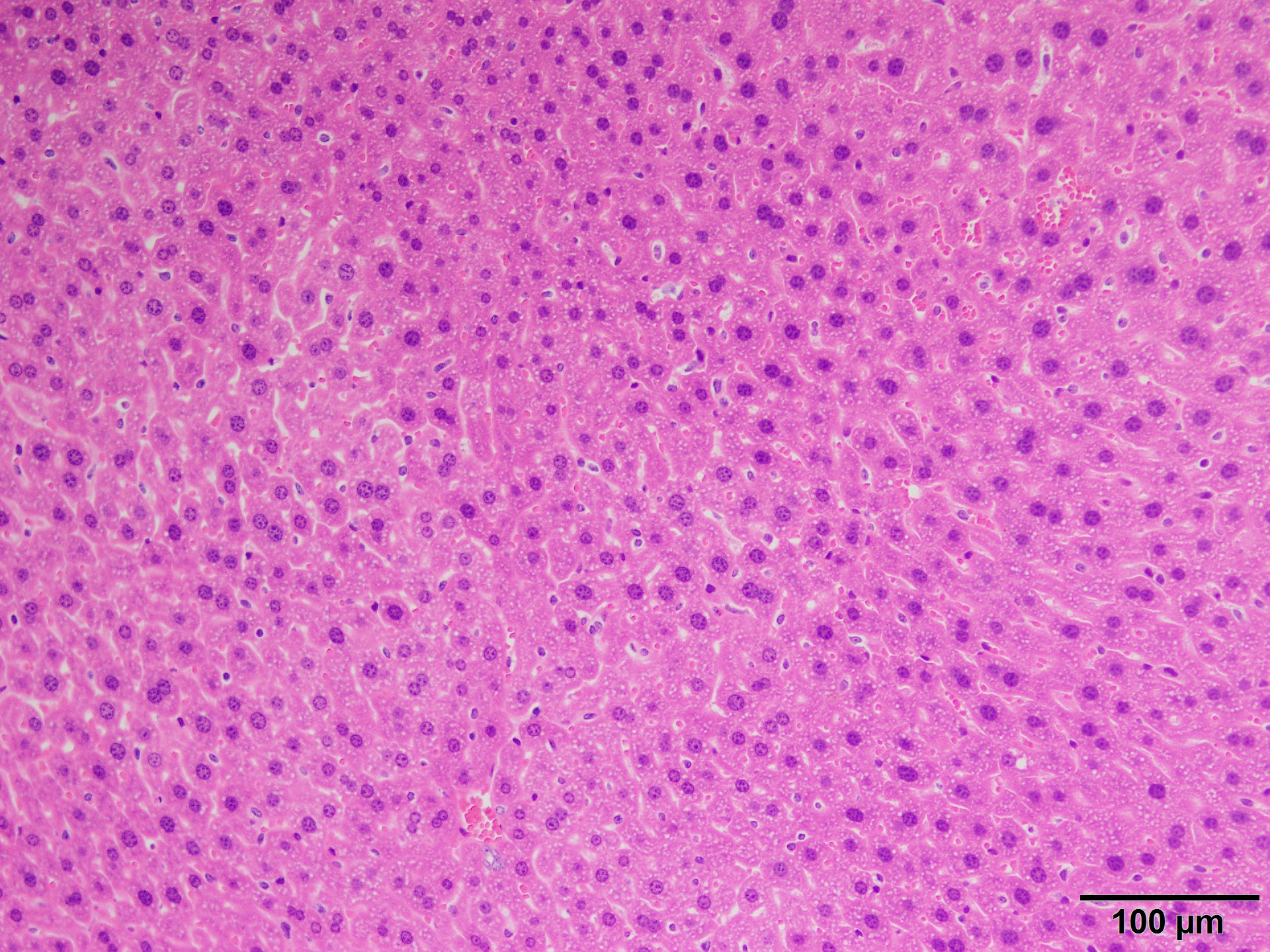

Supplement: Supplementary file 1 [file Data_Sheet_1.ZIP › Raw Data/H&E of Liver/YF01/6-3-4.jpg]

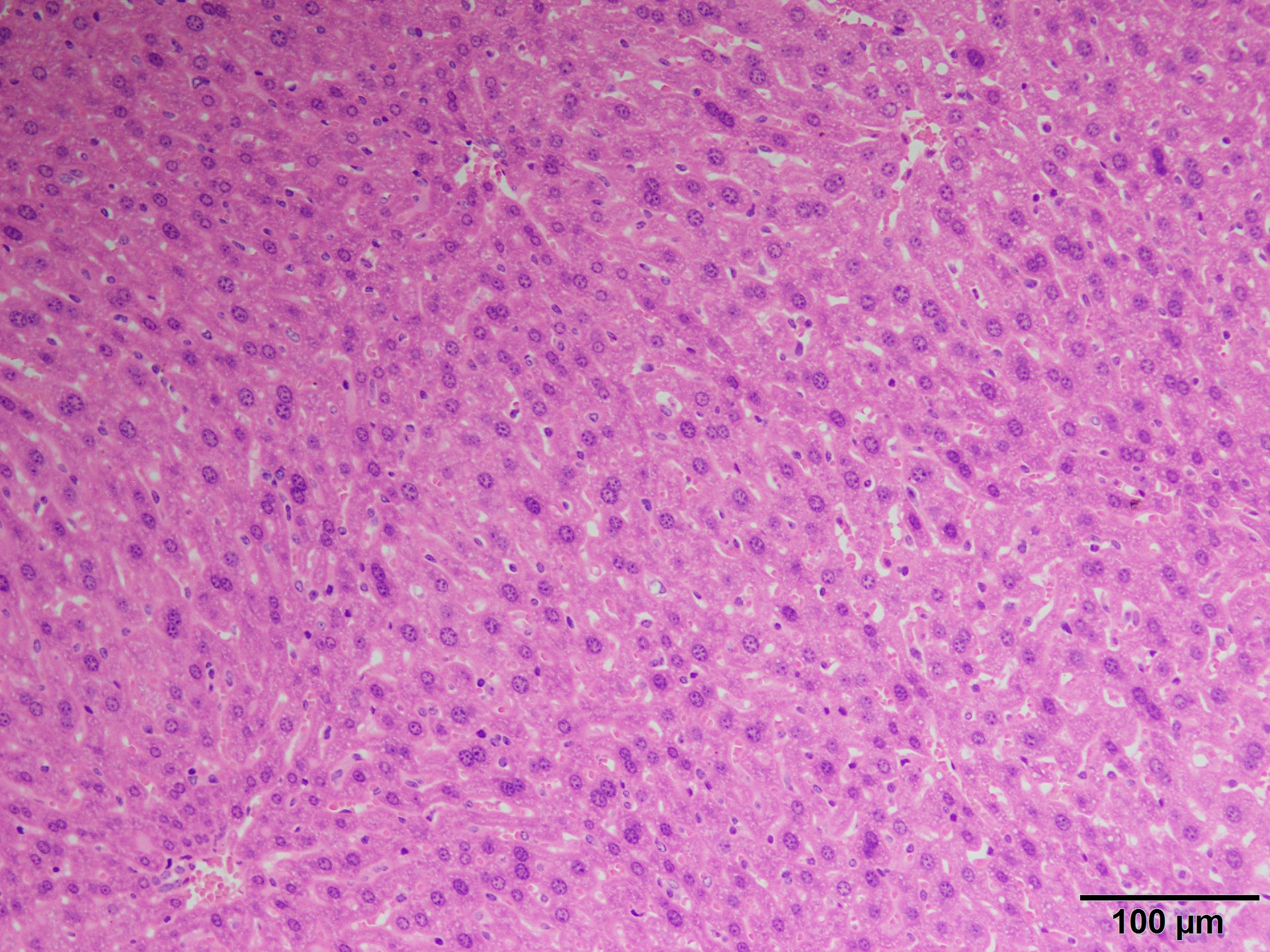

Supplement: Supplementary file 1 [file Data_Sheet_1.ZIP › Raw Data/H&E of Liver/YF01/6-9-3.jpg]

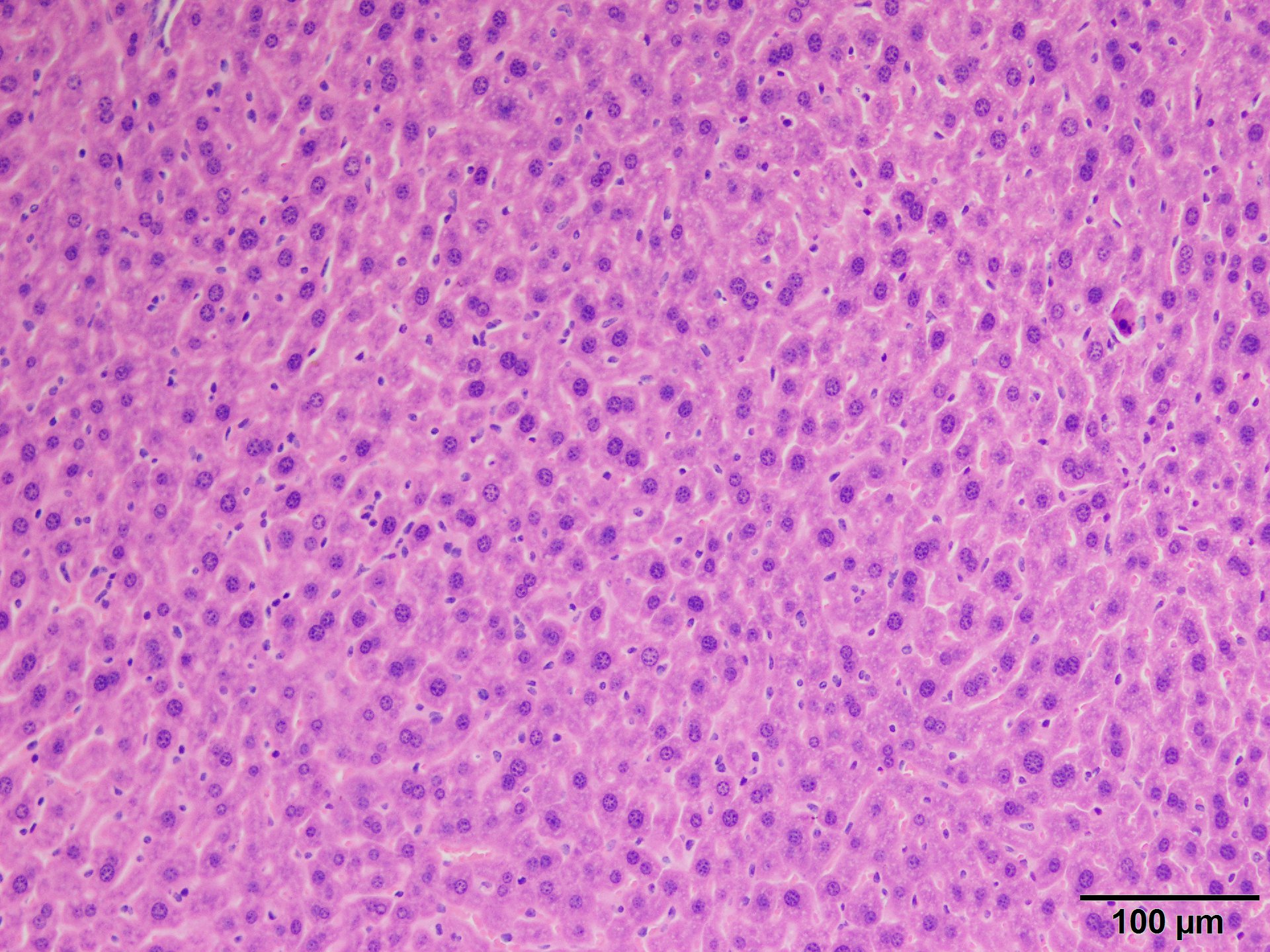

Supplement: Supplementary file 1 [file Data_Sheet_1.ZIP › Raw Data/H&E of Liver/YF01/select-6-10-1.jpg]

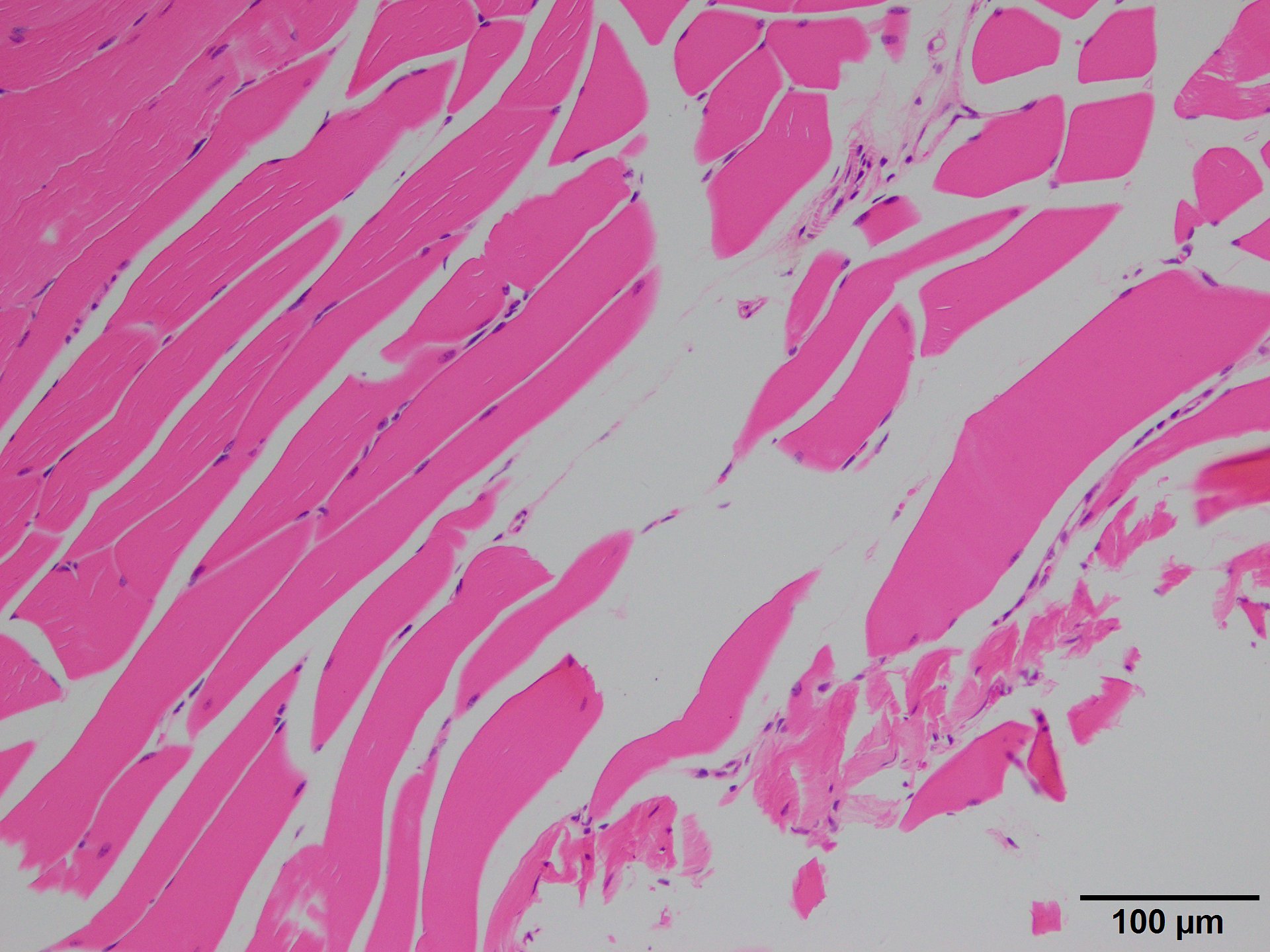

Supplement: Supplementary file 1 [file Data_Sheet_1.ZIP › Raw Data/H&E of Muscle/control/2-22-2.jpg]

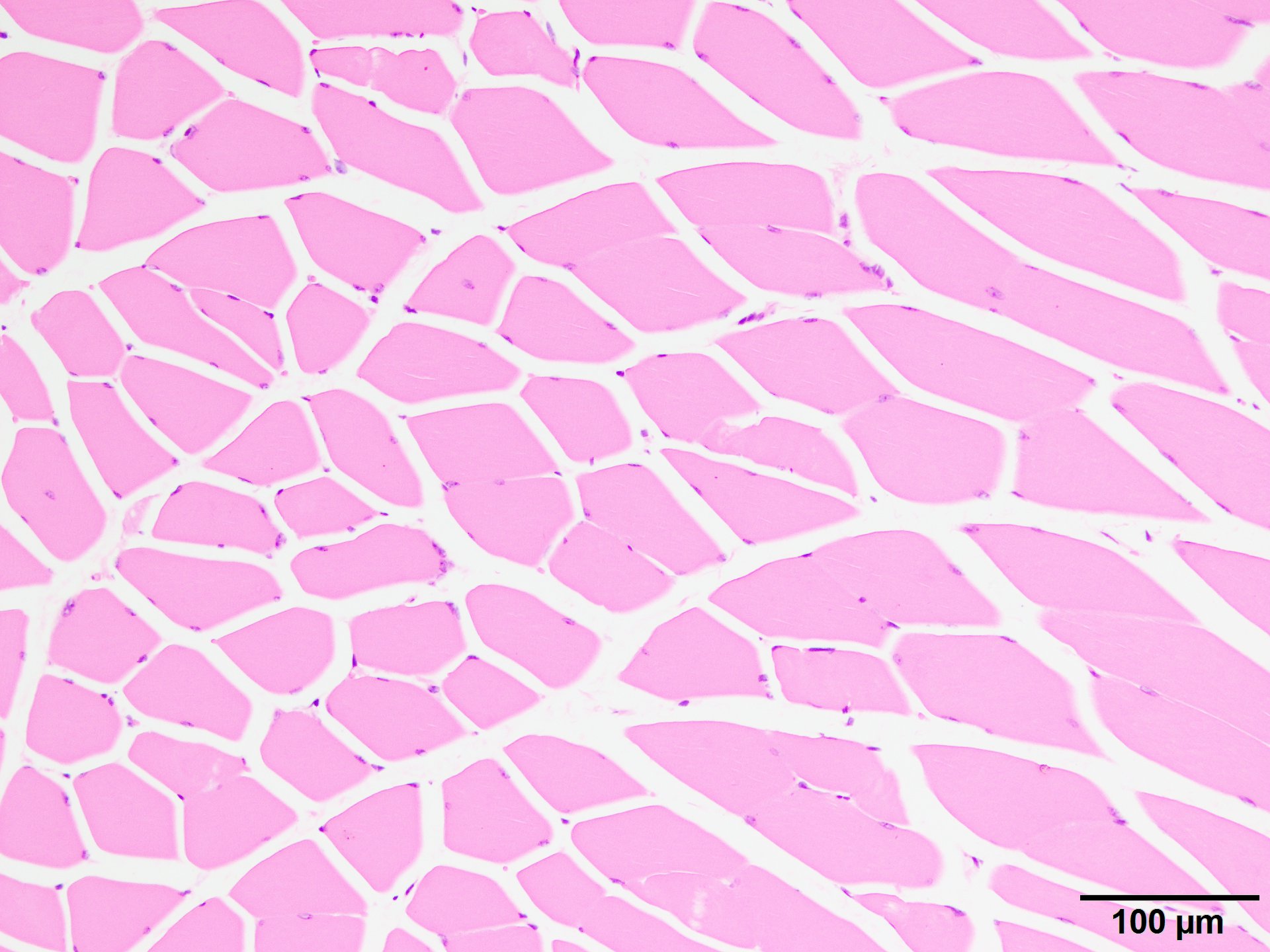

Supplement: Supplementary file 1 [file Data_Sheet_1.ZIP › Raw Data/H&E of Muscle/control/2-5-5.jpg]

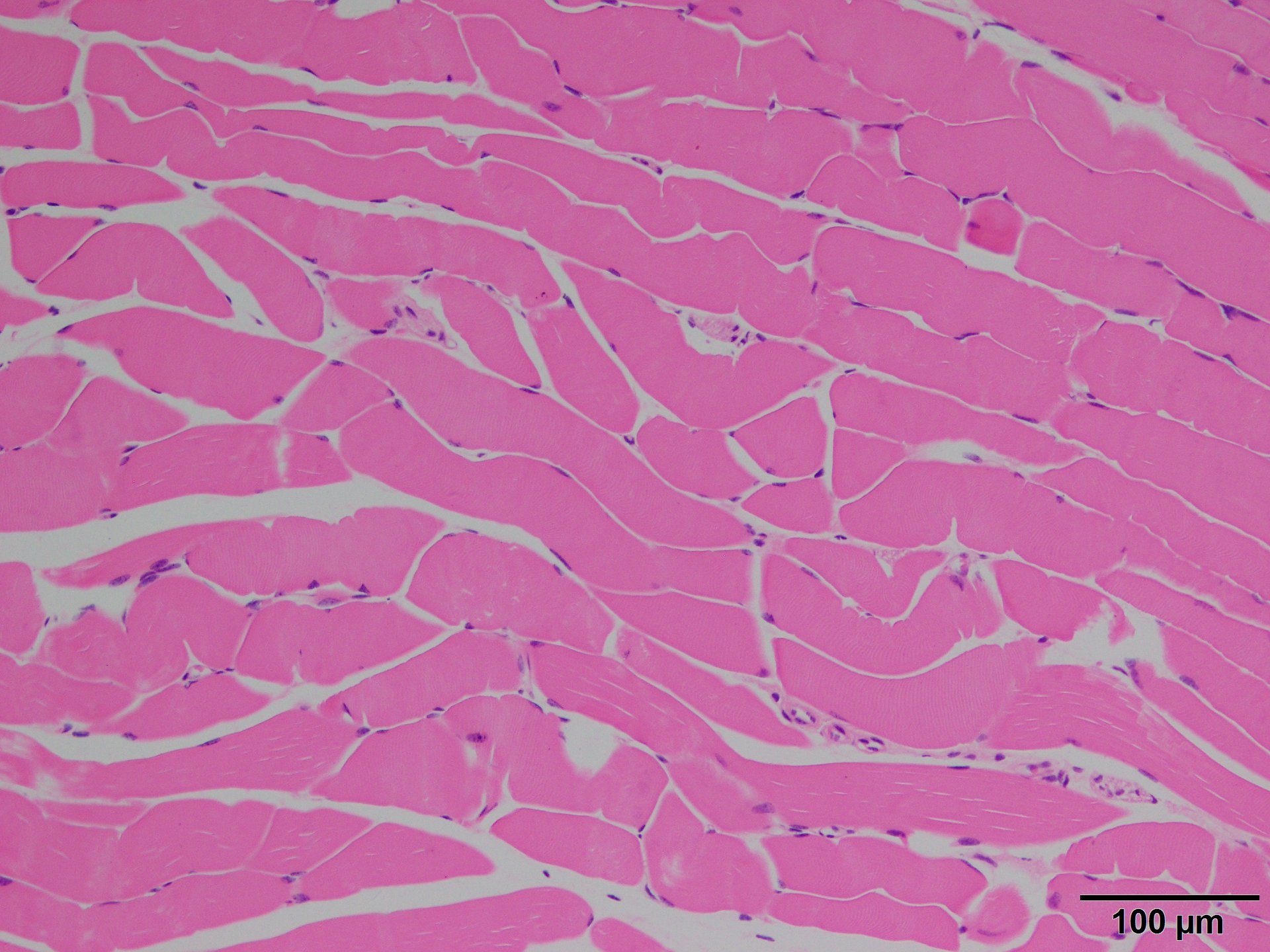

Supplement: Supplementary file 1 [file Data_Sheet_1.ZIP › Raw Data/H&E of Muscle/control/select-2-3-3.jpg]

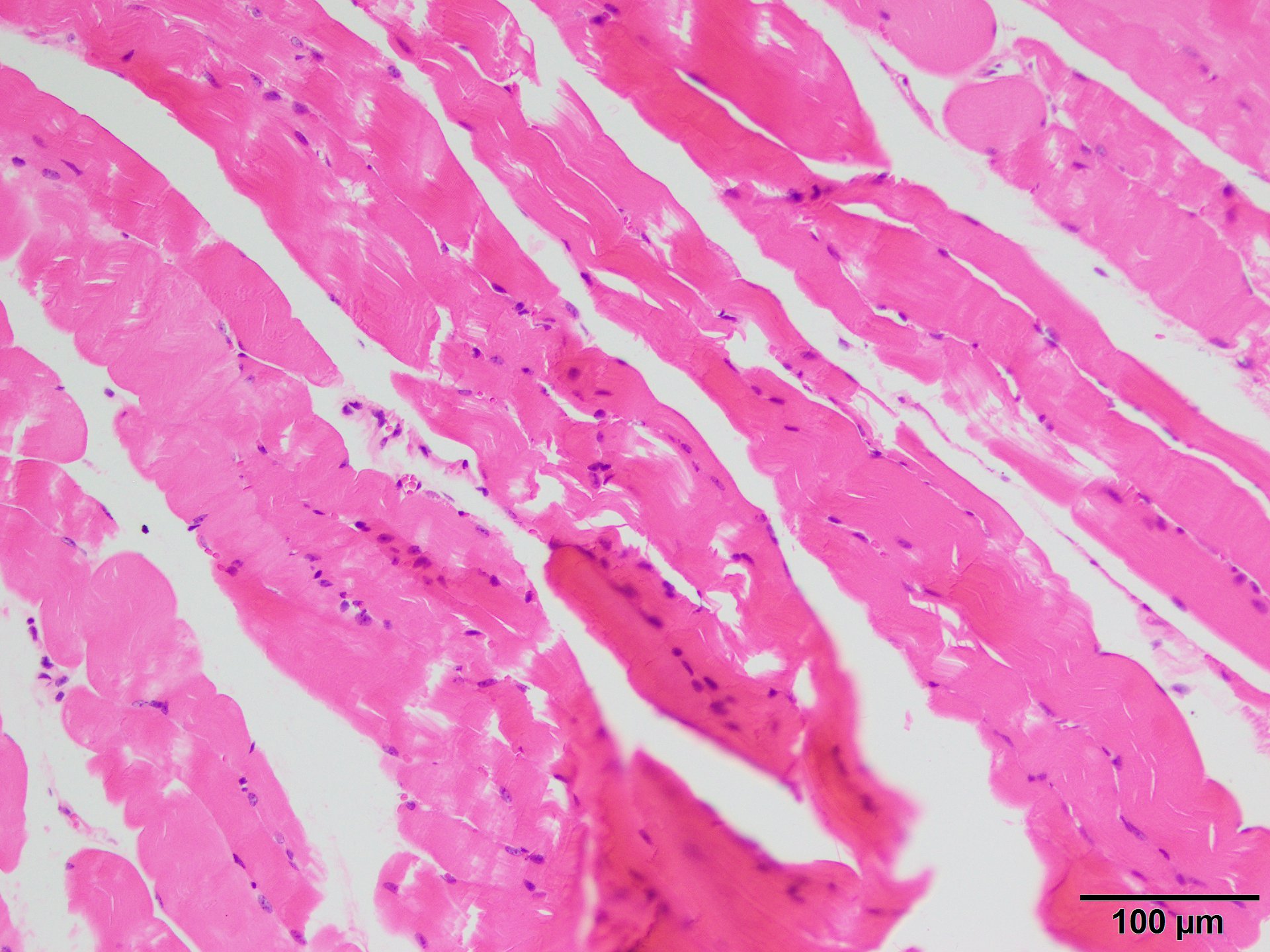

Supplement: Supplementary file 1 [file Data_Sheet_1.ZIP › Raw Data/H&E of Muscle/normal/1-26-5.jpg]

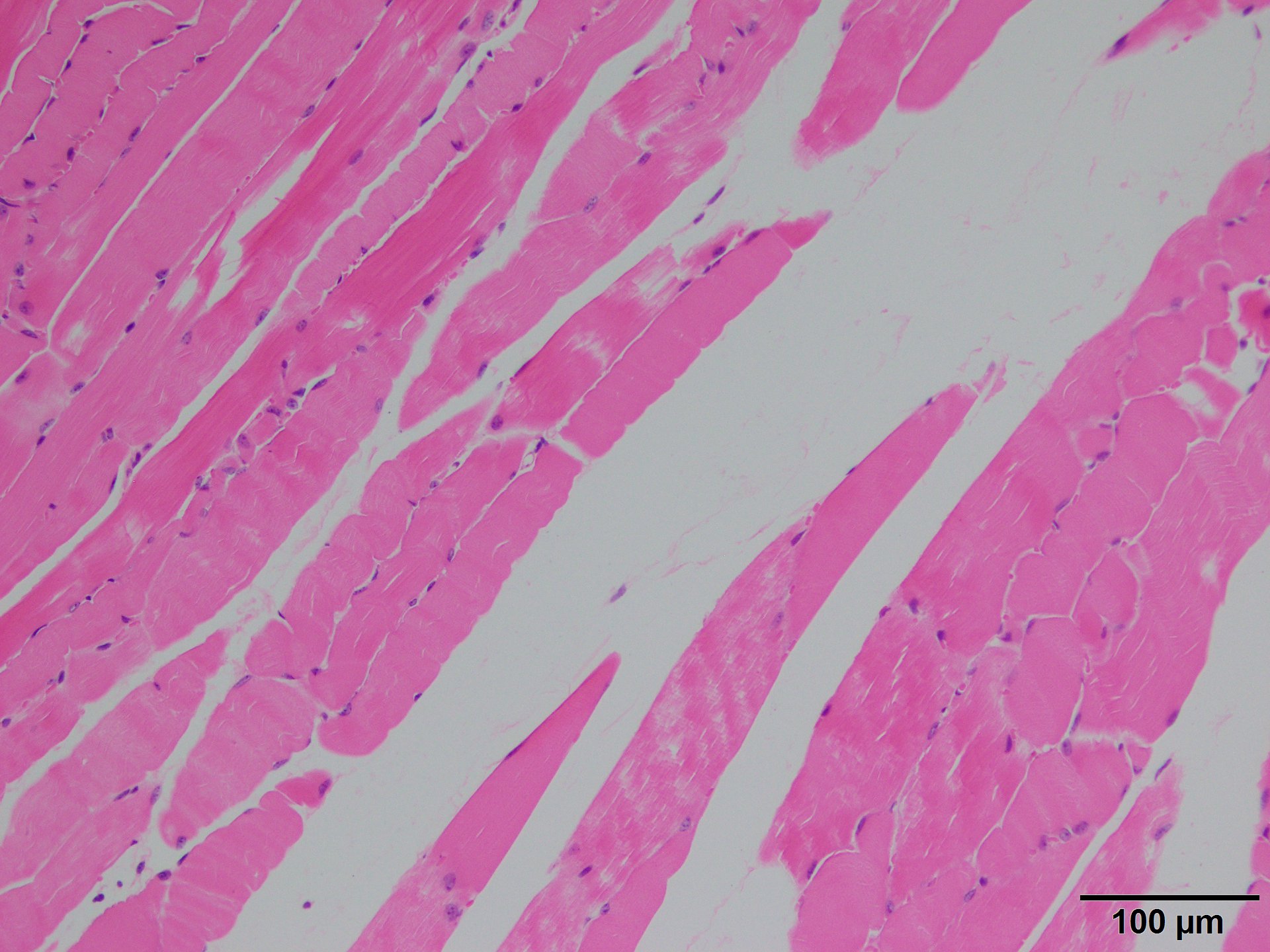

Supplement: Supplementary file 1 [file Data_Sheet_1.ZIP › Raw Data/H&E of Muscle/normal/1-27-5.jpg]

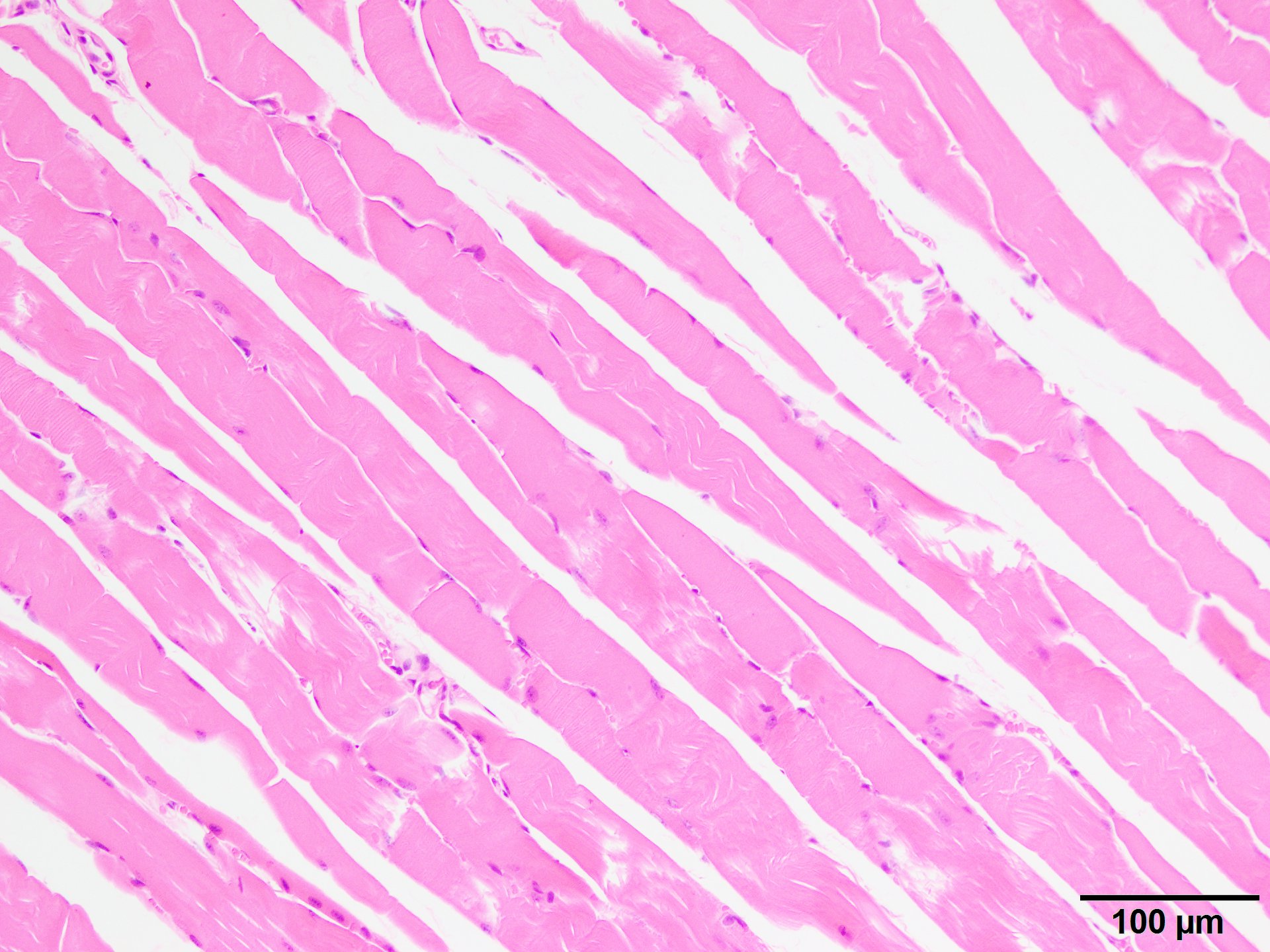

Supplement: Supplementary file 1 [file Data_Sheet_1.ZIP › Raw Data/H&E of Muscle/normal/select-1-10-3.jpg]

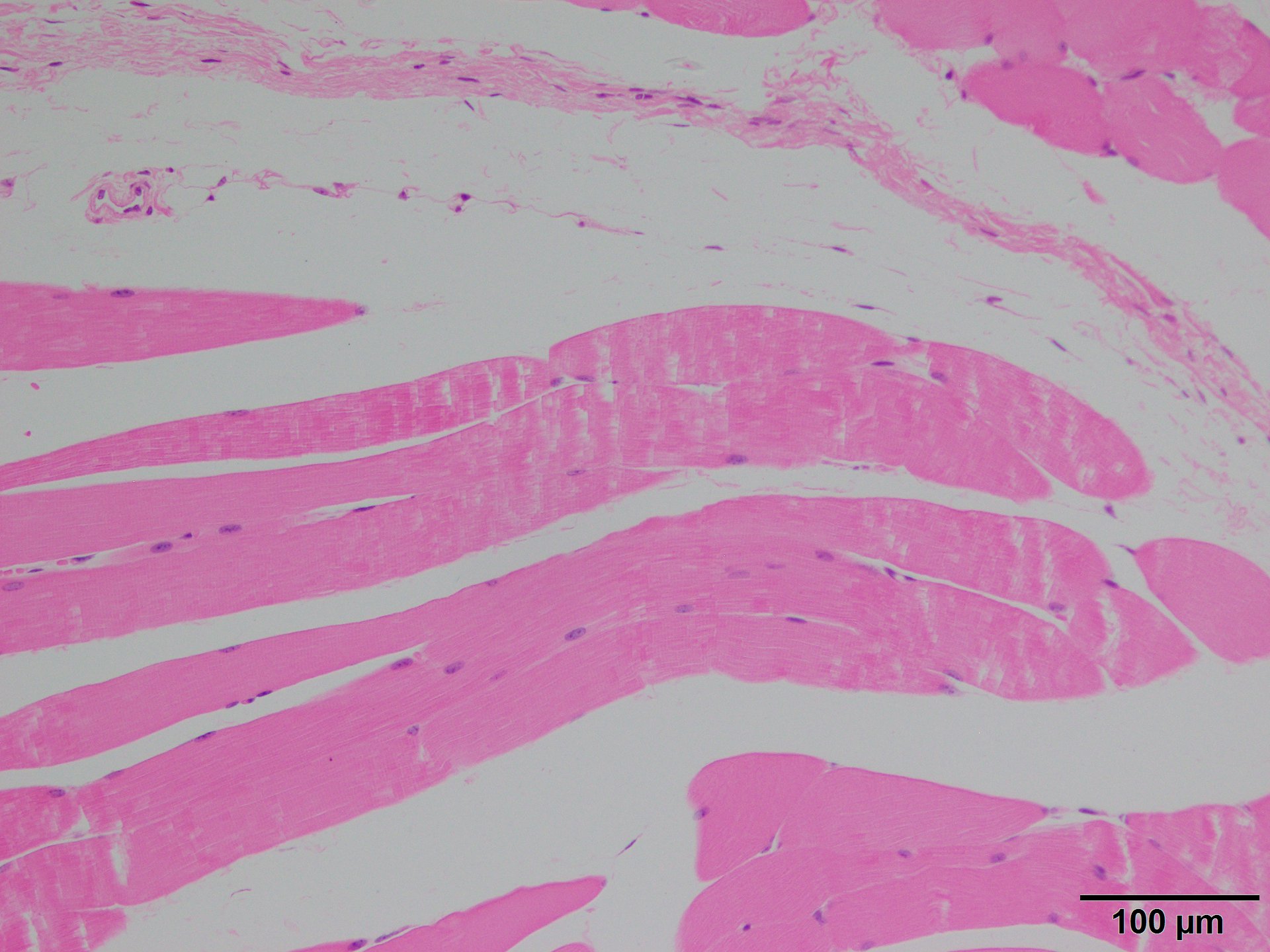

Supplement: Supplementary file 1 [file Data_Sheet_1.ZIP › Raw Data/H&E of Muscle/Vc/3-7-3.jpg]

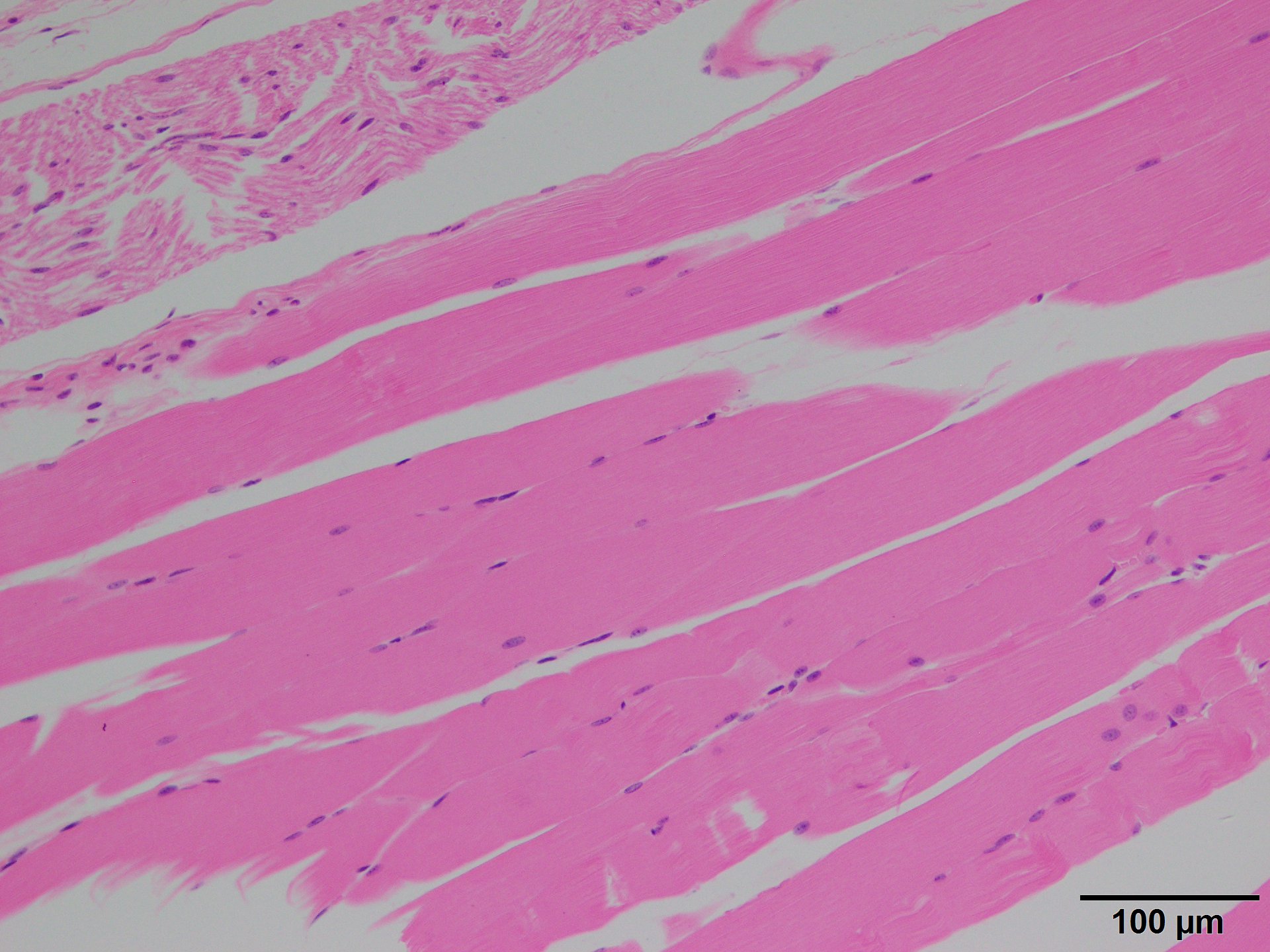

Supplement: Supplementary file 1 [file Data_Sheet_1.ZIP › Raw Data/H&E of Muscle/Vc/3-7-4.jpg]

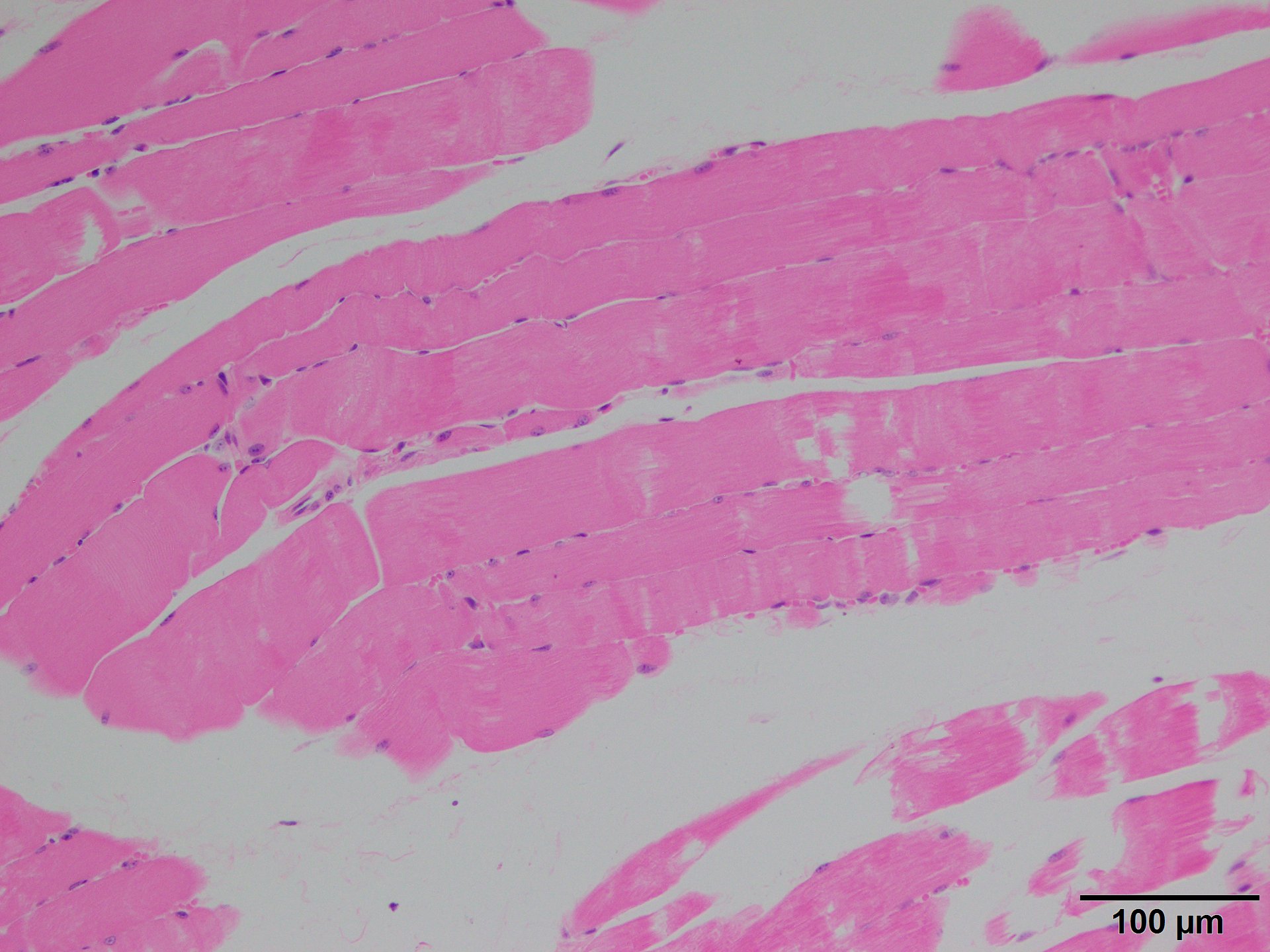

Supplement: Supplementary file 1 [file Data_Sheet_1.ZIP › Raw Data/H&E of Muscle/Vc/3-7-6.jpg]

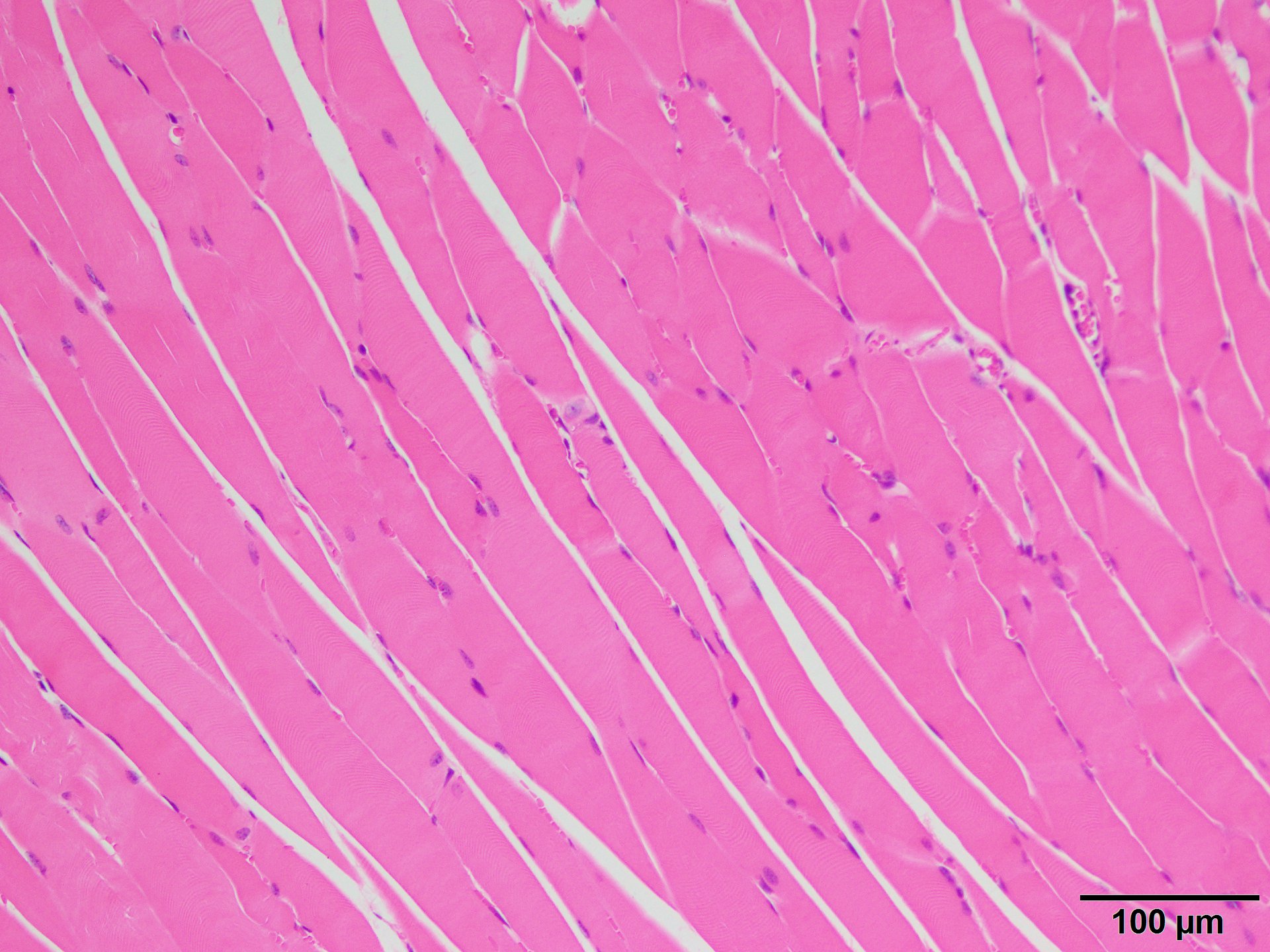

Supplement: Supplementary file 1 [file Data_Sheet_1.ZIP › Raw Data/H&E of Muscle/Vc/select-3-27-4.jpg]

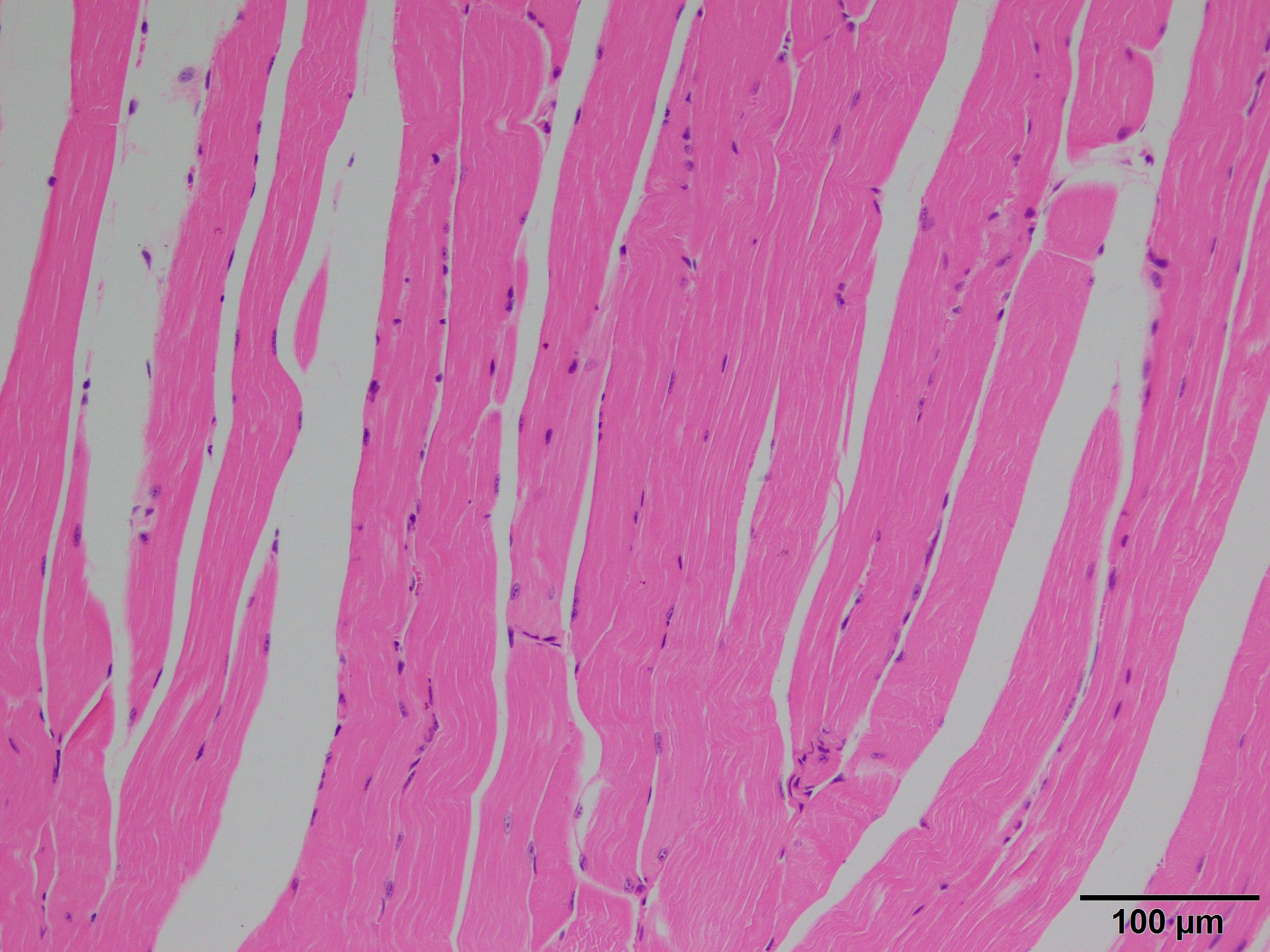

Supplement: Supplementary file 1 [file Data_Sheet_1.ZIP › Raw Data/H&E of Muscle/YF01/6-4-1.jpg]

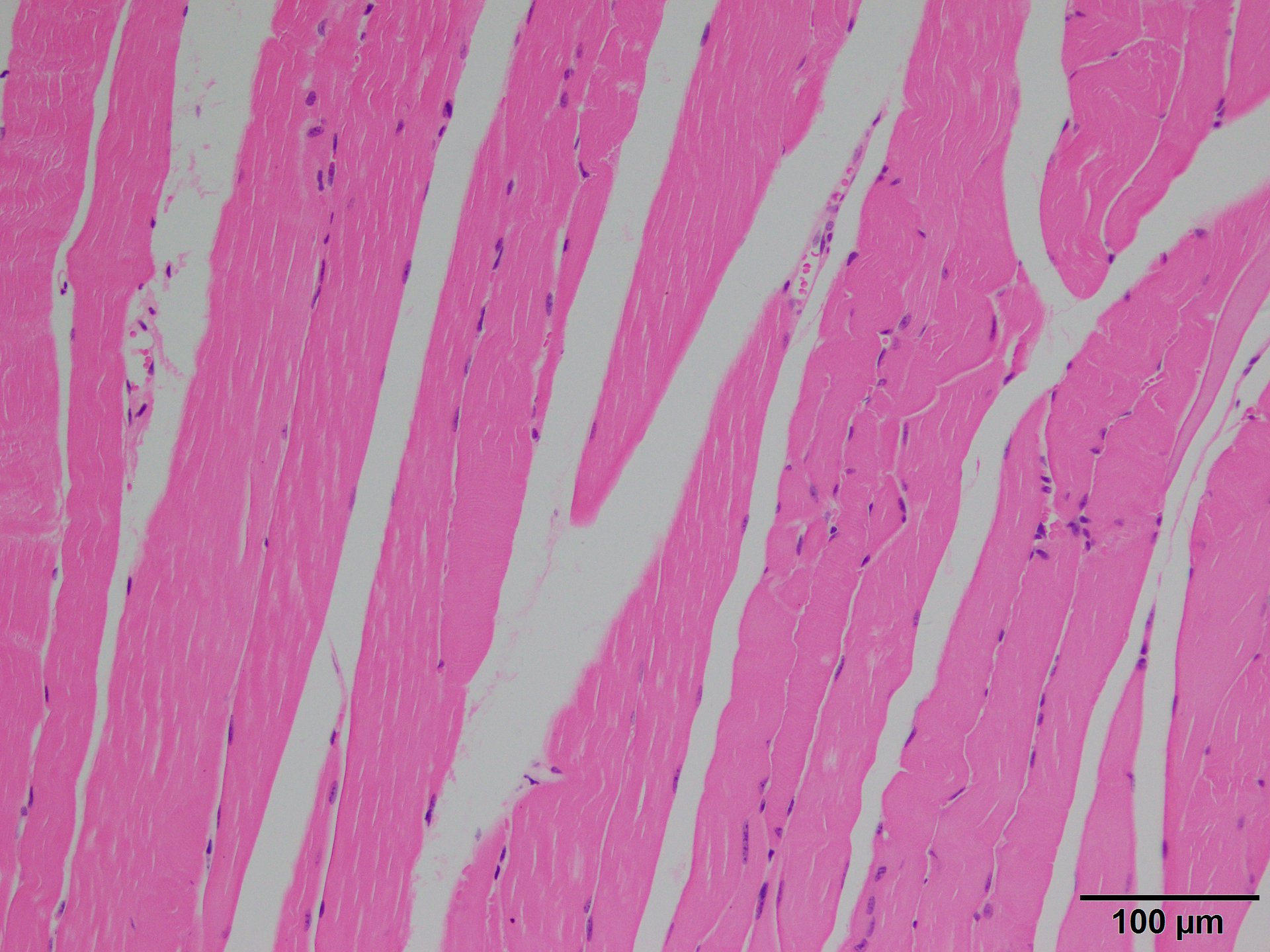

Supplement: Supplementary file 1 [file Data_Sheet_1.ZIP › Raw Data/H&E of Muscle/YF01/6-4-5.jpg]

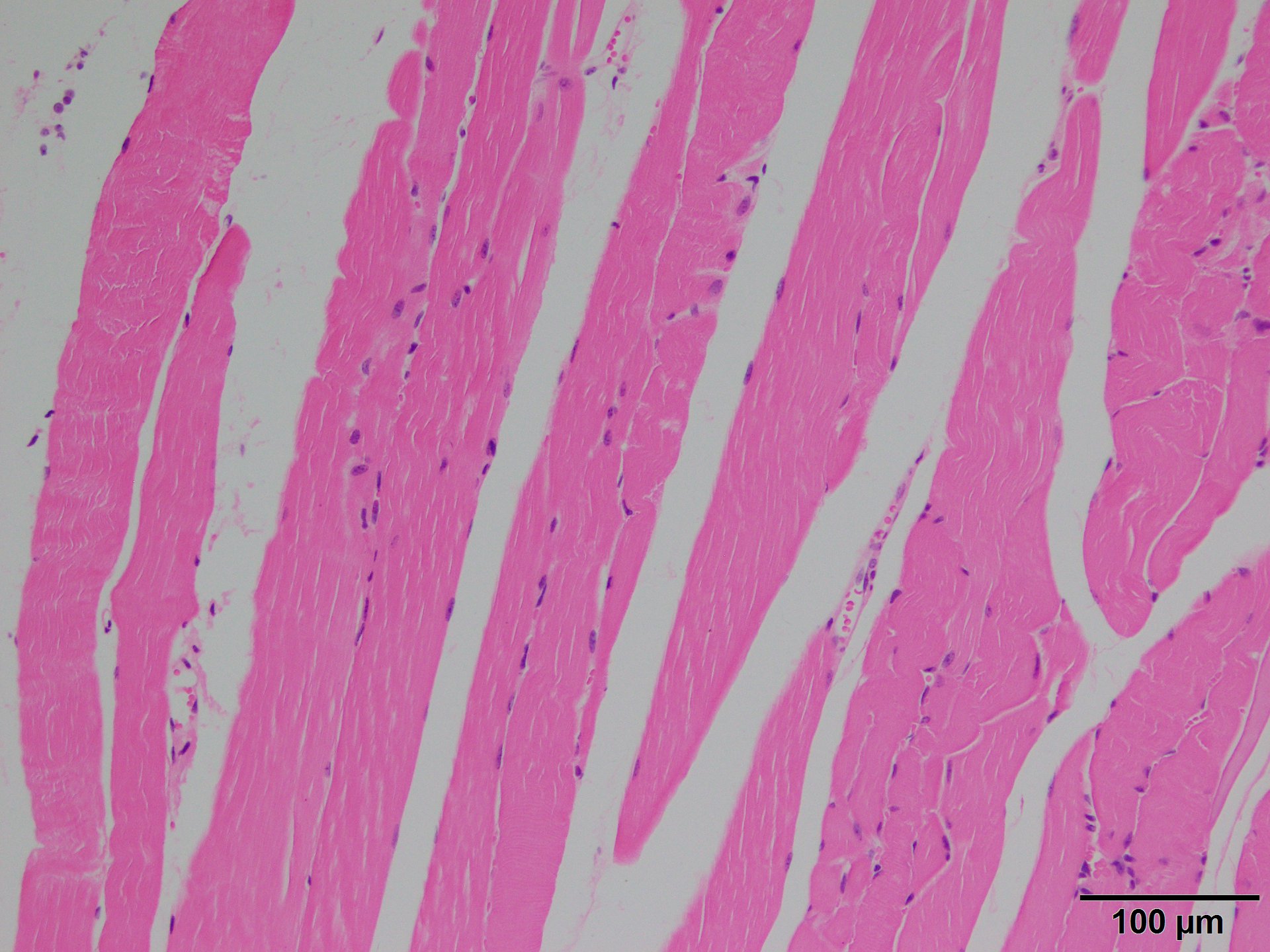

Supplement: Supplementary file 1 [file Data_Sheet_1.ZIP › Raw Data/H&E of Muscle/YF01/6-4-6.jpg]

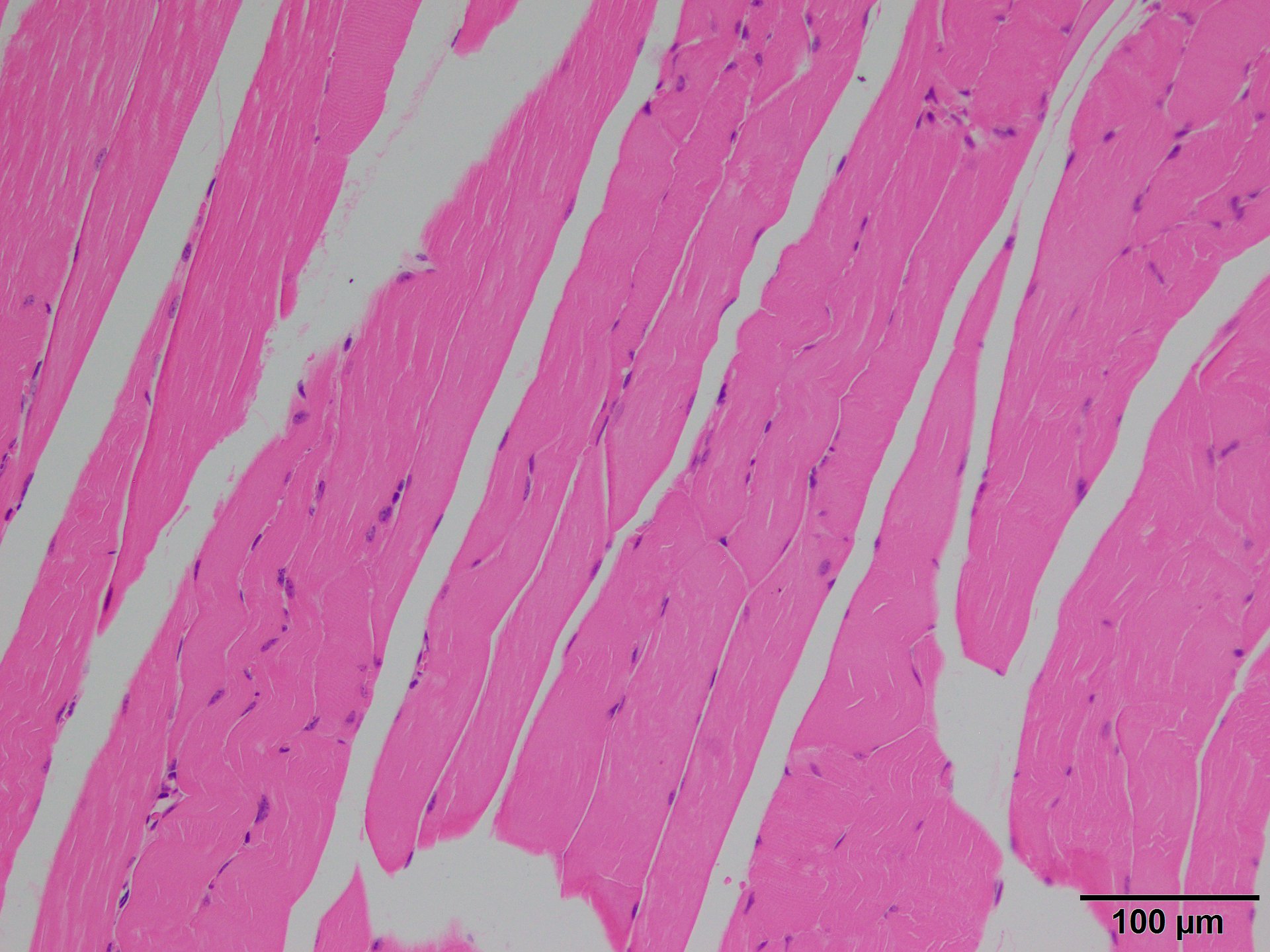

Supplement: Supplementary file 1 [file Data_Sheet_1.ZIP › Raw Data/H&E of Muscle/YF01/select-6-4-3.jpg]

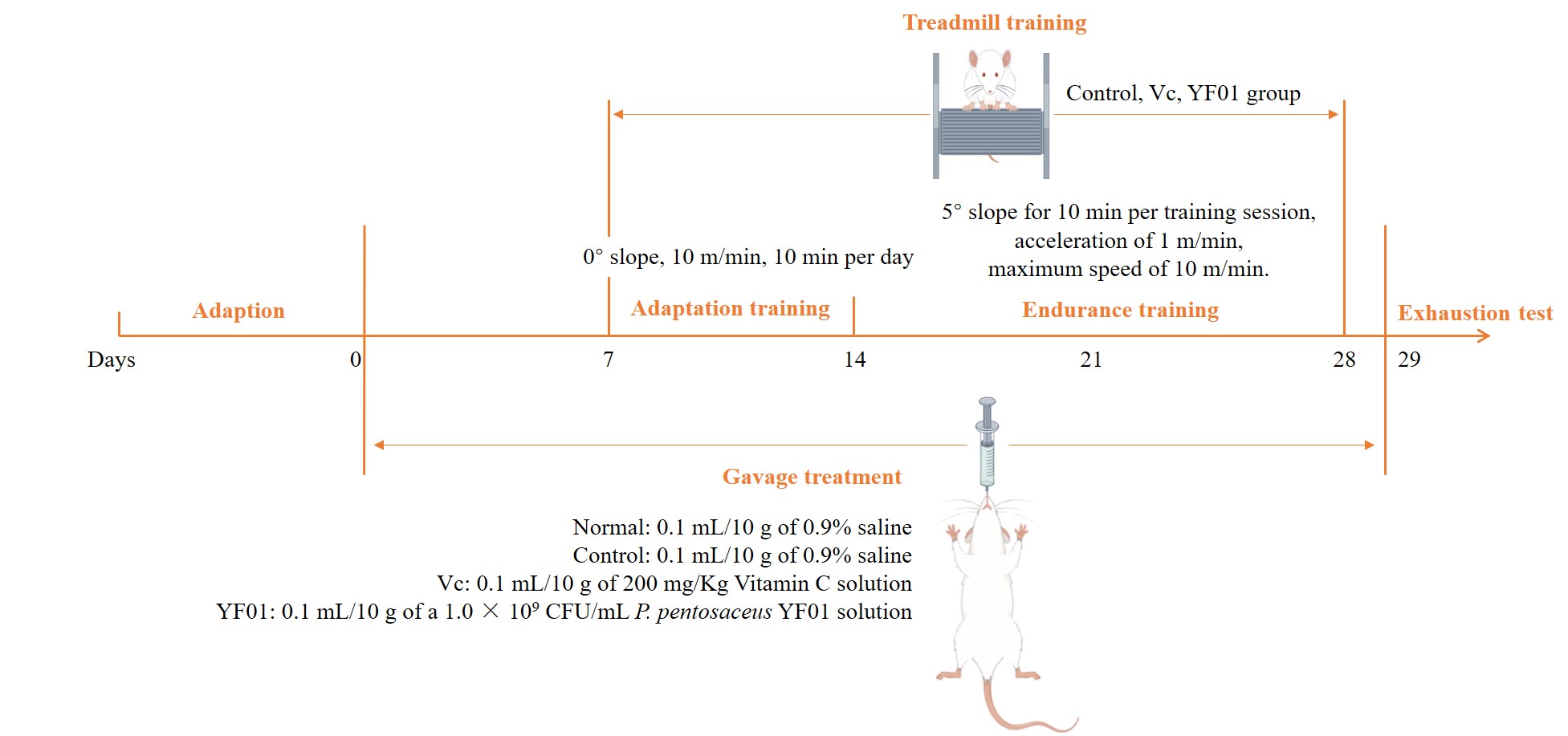

Supplement: Supplementary file 1 [file Data_Sheet_1.ZIP › All figure/FIGURE 1.jpg]

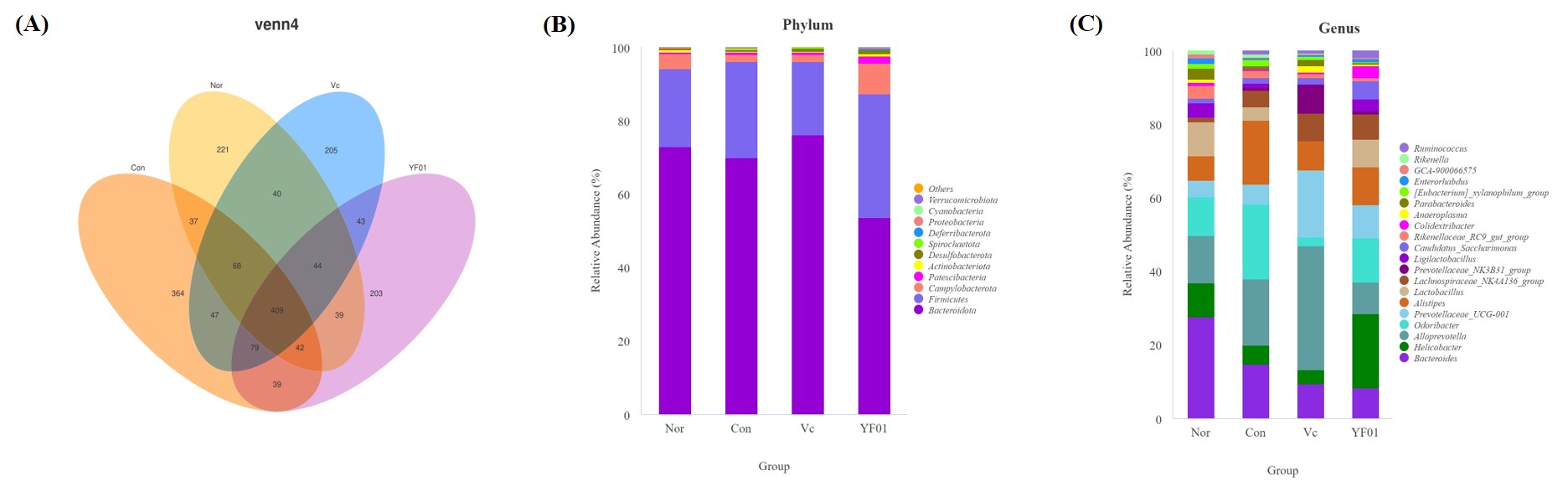

Supplement: Supplementary file 1 [file Data_Sheet_1.ZIP › All figure/FIGURE 10.jpg]

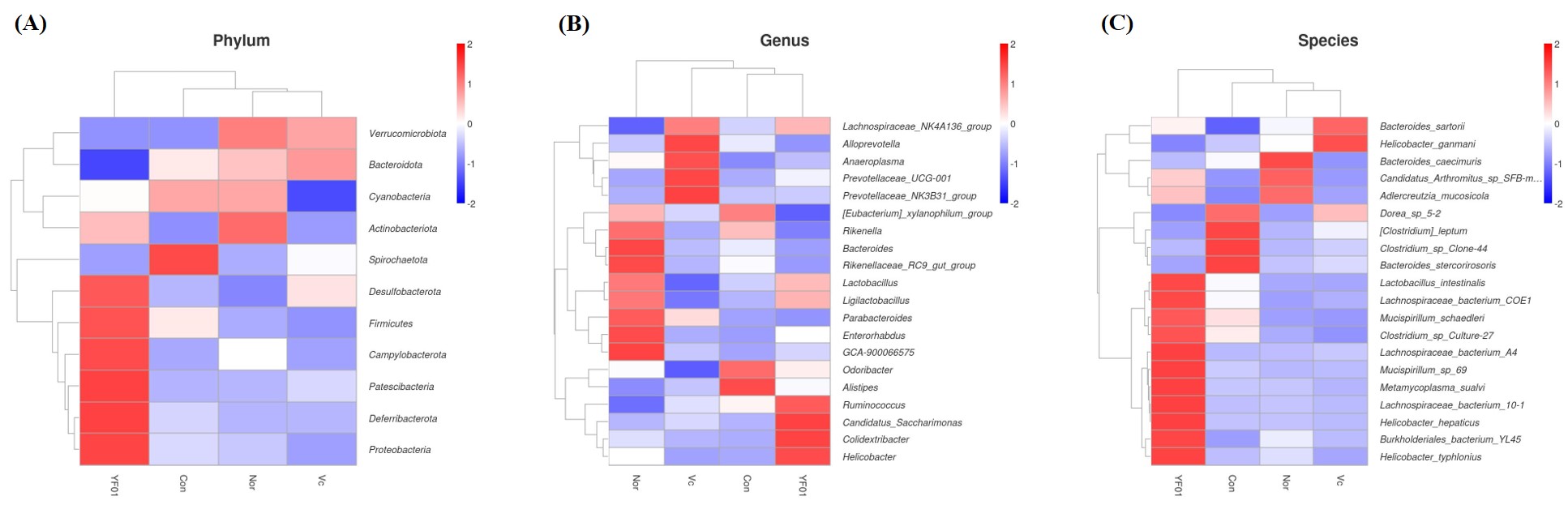

Supplement: Supplementary file 1 [file Data_Sheet_1.ZIP › All figure/FIGURE 11.jpg]

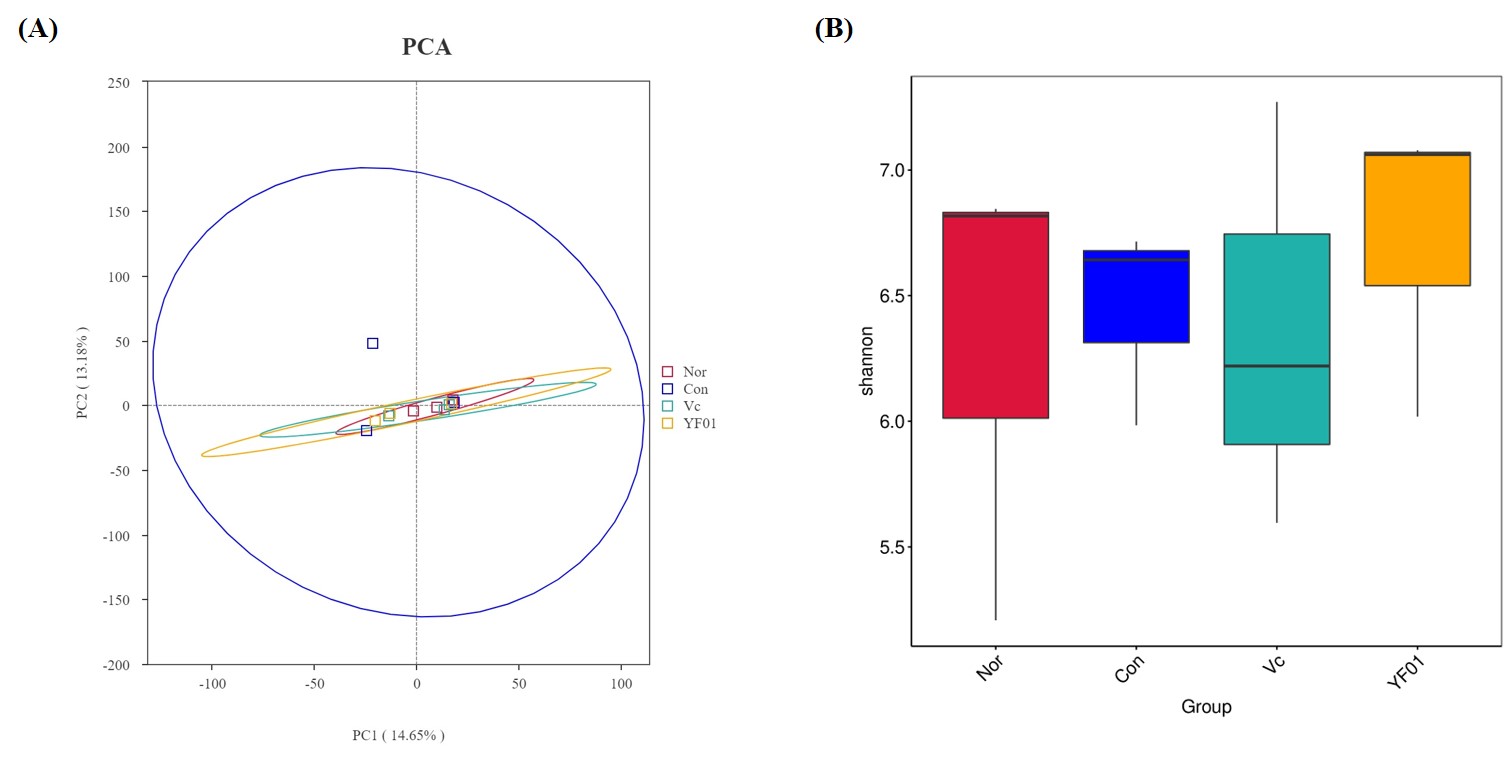

Supplement: Supplementary file 1 [file Data_Sheet_1.ZIP › All figure/FIGURE 12.jpg]

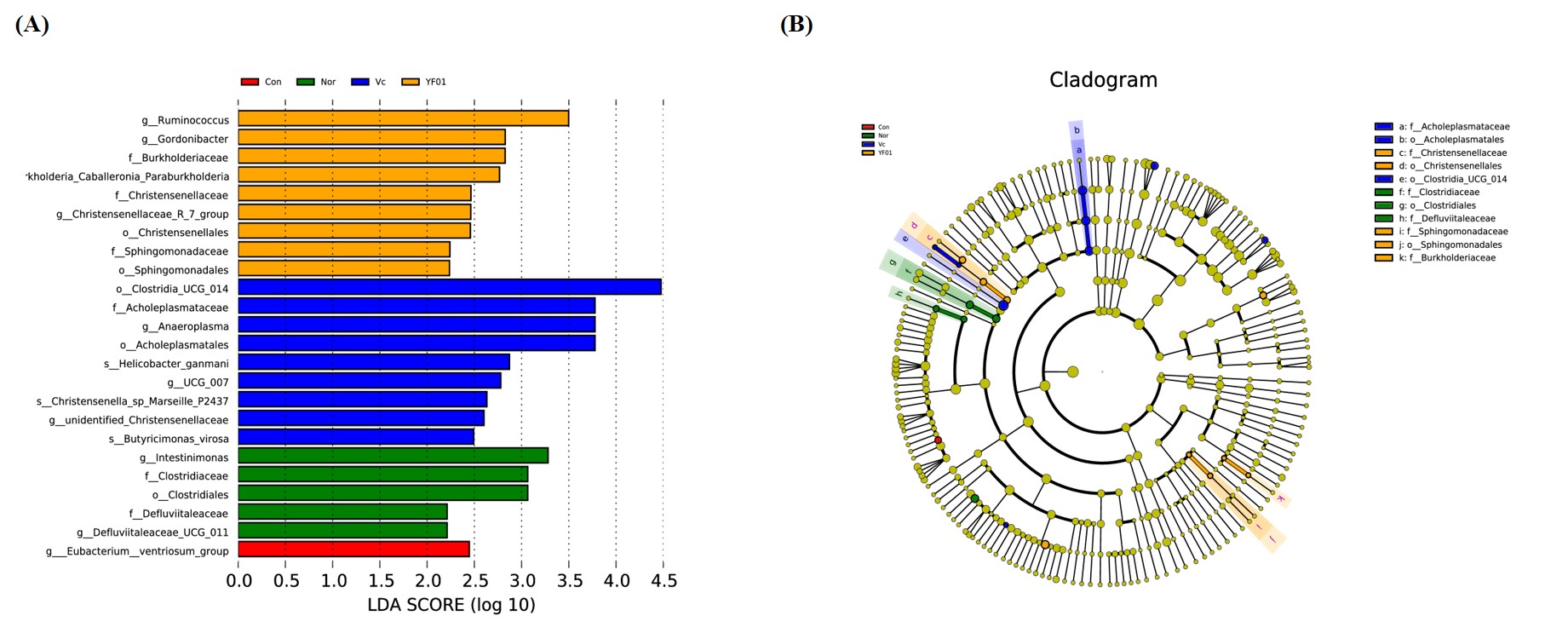

Supplement: Supplementary file 1 [file Data_Sheet_1.ZIP › All figure/FIGURE 13.jpg]

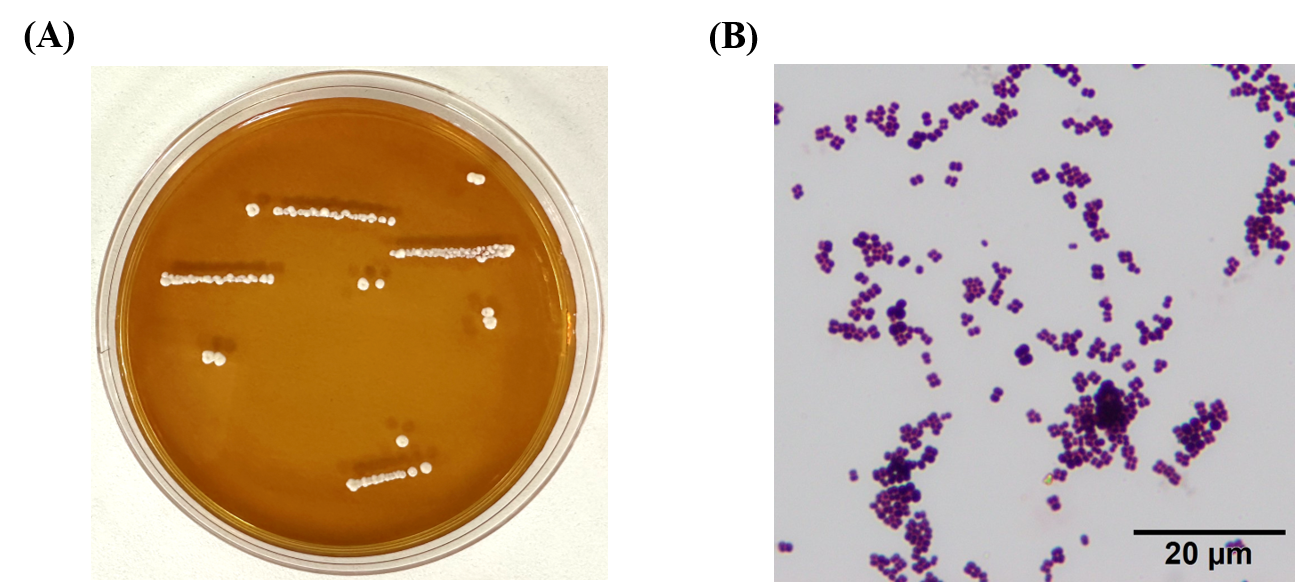

Supplement: Supplementary file 1 [file Data_Sheet_1.ZIP › All figure/FIGURE 2.jpg]

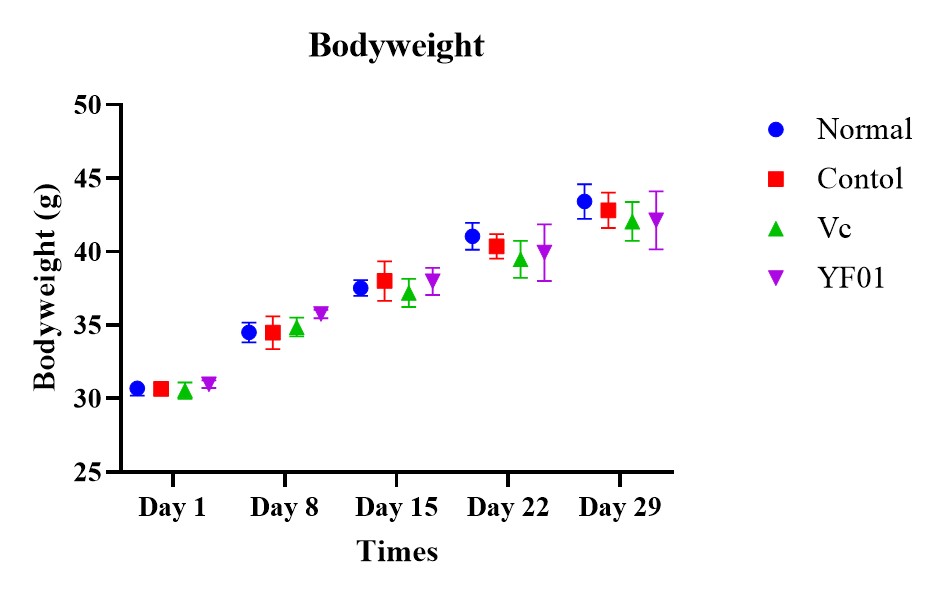

Supplement: Supplementary file 1 [file Data_Sheet_1.ZIP › All figure/FIGURE 3.jpg]

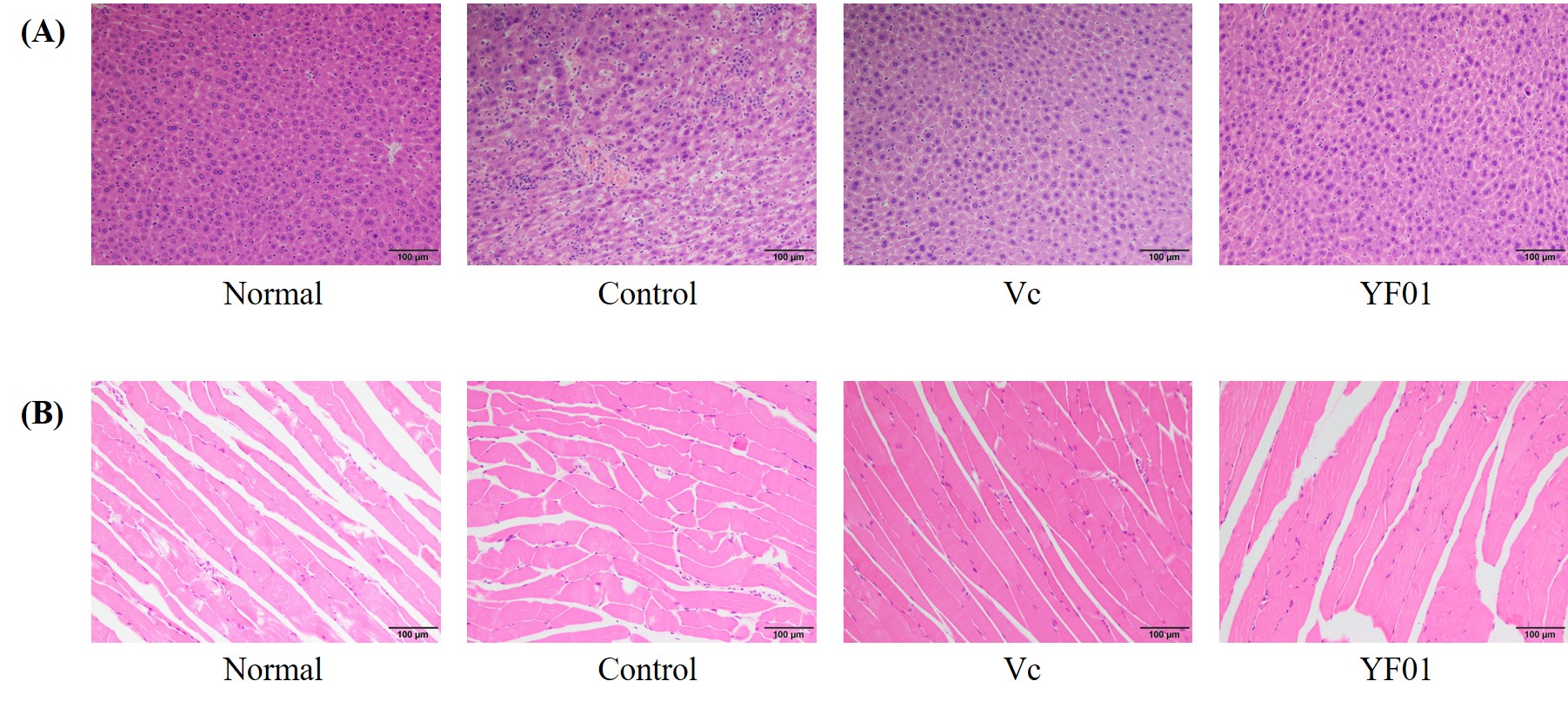

Supplement: Supplementary file 1 [file Data_Sheet_1.ZIP › All figure/FIGURE 4.jpg]

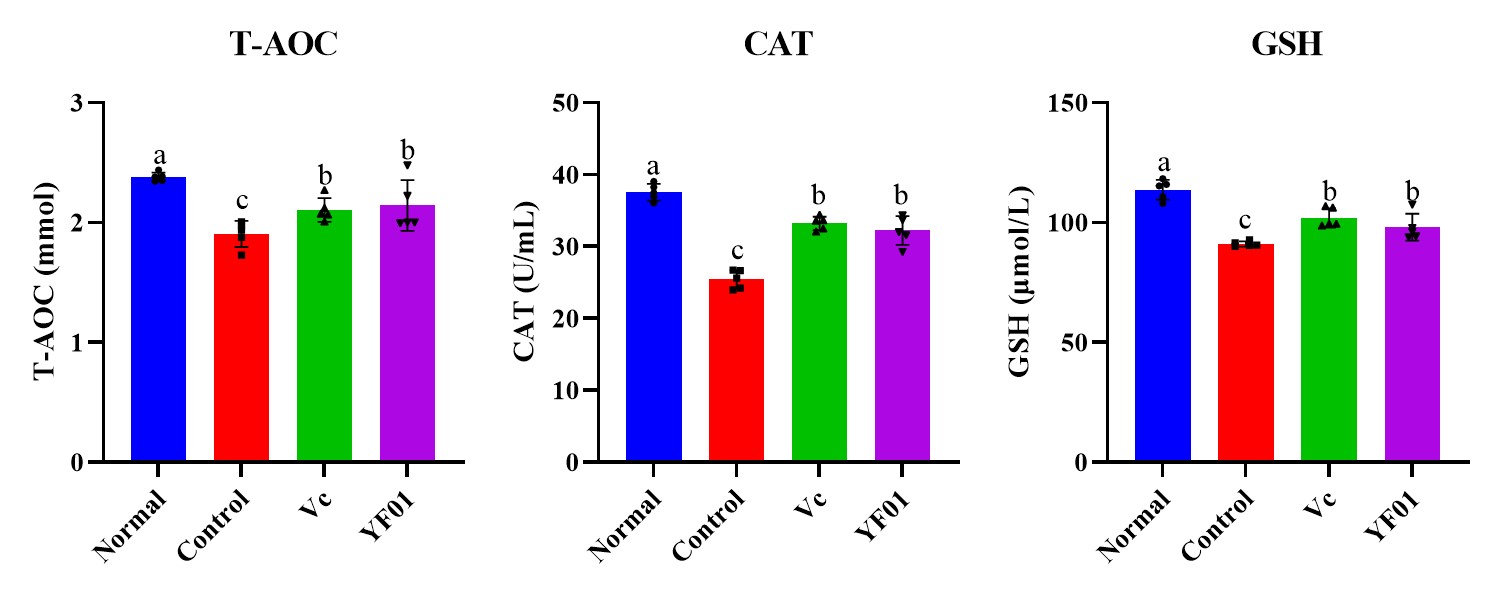

Supplement: Supplementary file 1 [file Data_Sheet_1.ZIP › All figure/FIGURE 5.jpg]

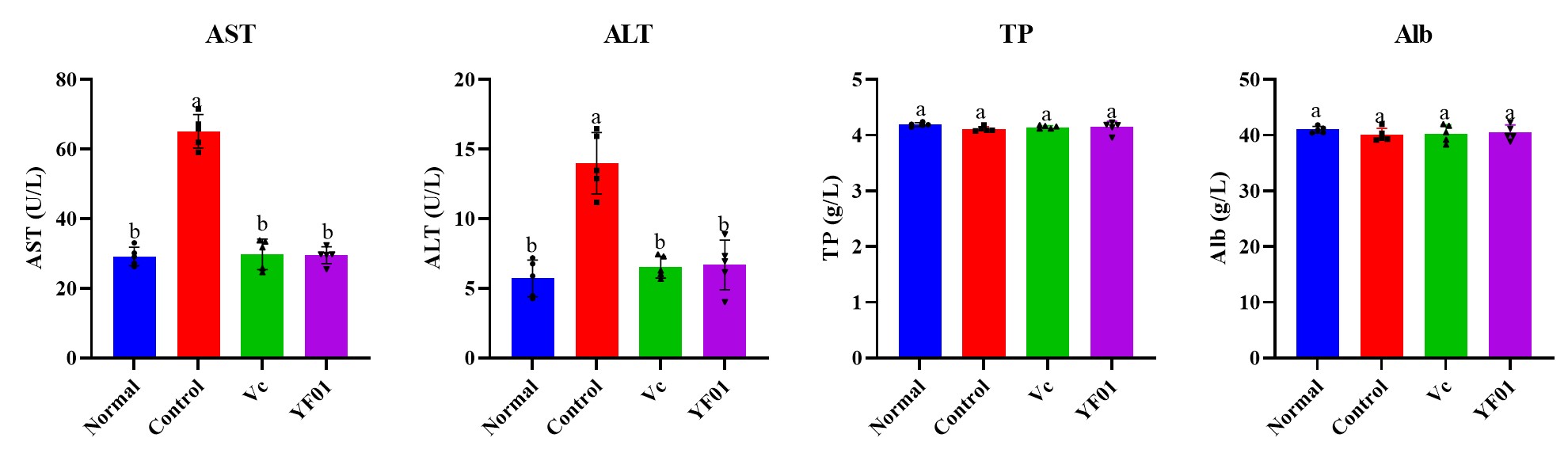

Supplement: Supplementary file 1 [file Data_Sheet_1.ZIP › All figure/FIGURE 6.jpg]

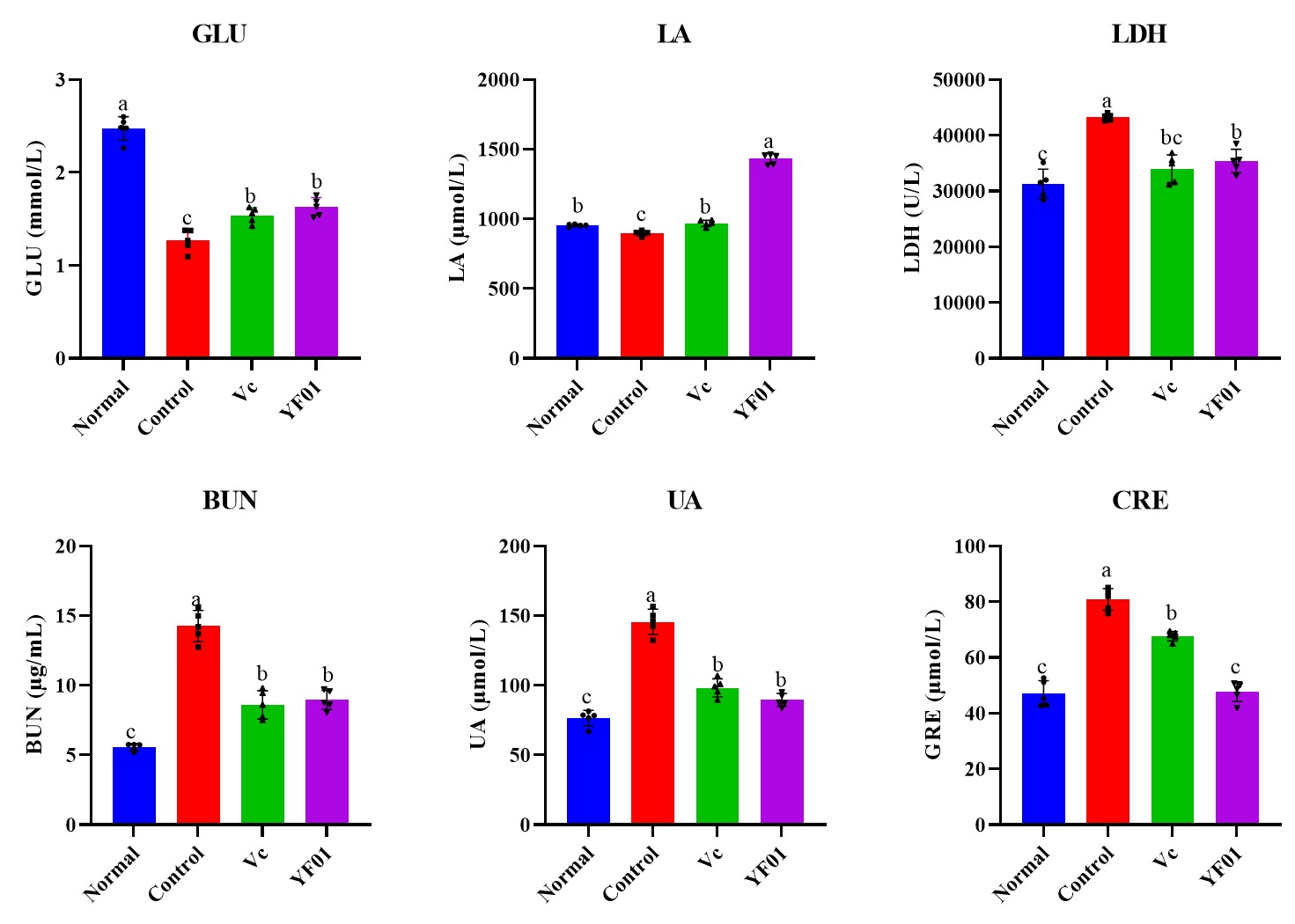

Supplement: Supplementary file 1 [file Data_Sheet_1.ZIP › All figure/FIGURE 7.jpg]

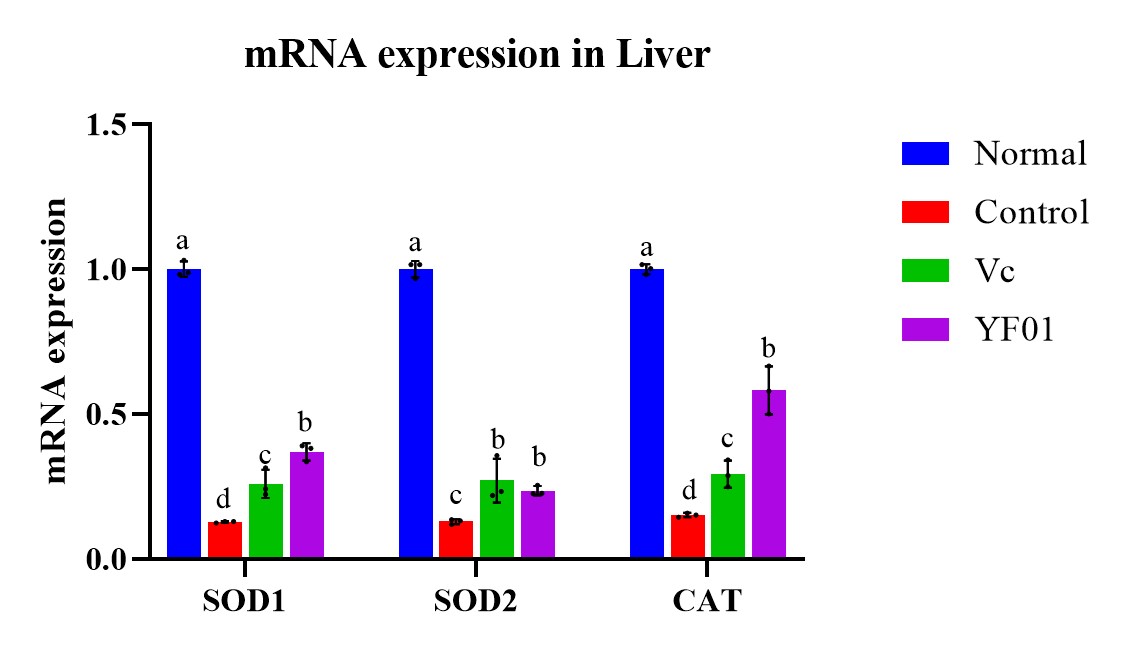

Supplement: Supplementary file 1 [file Data_Sheet_1.ZIP › All figure/FIGURE 8.jpg]

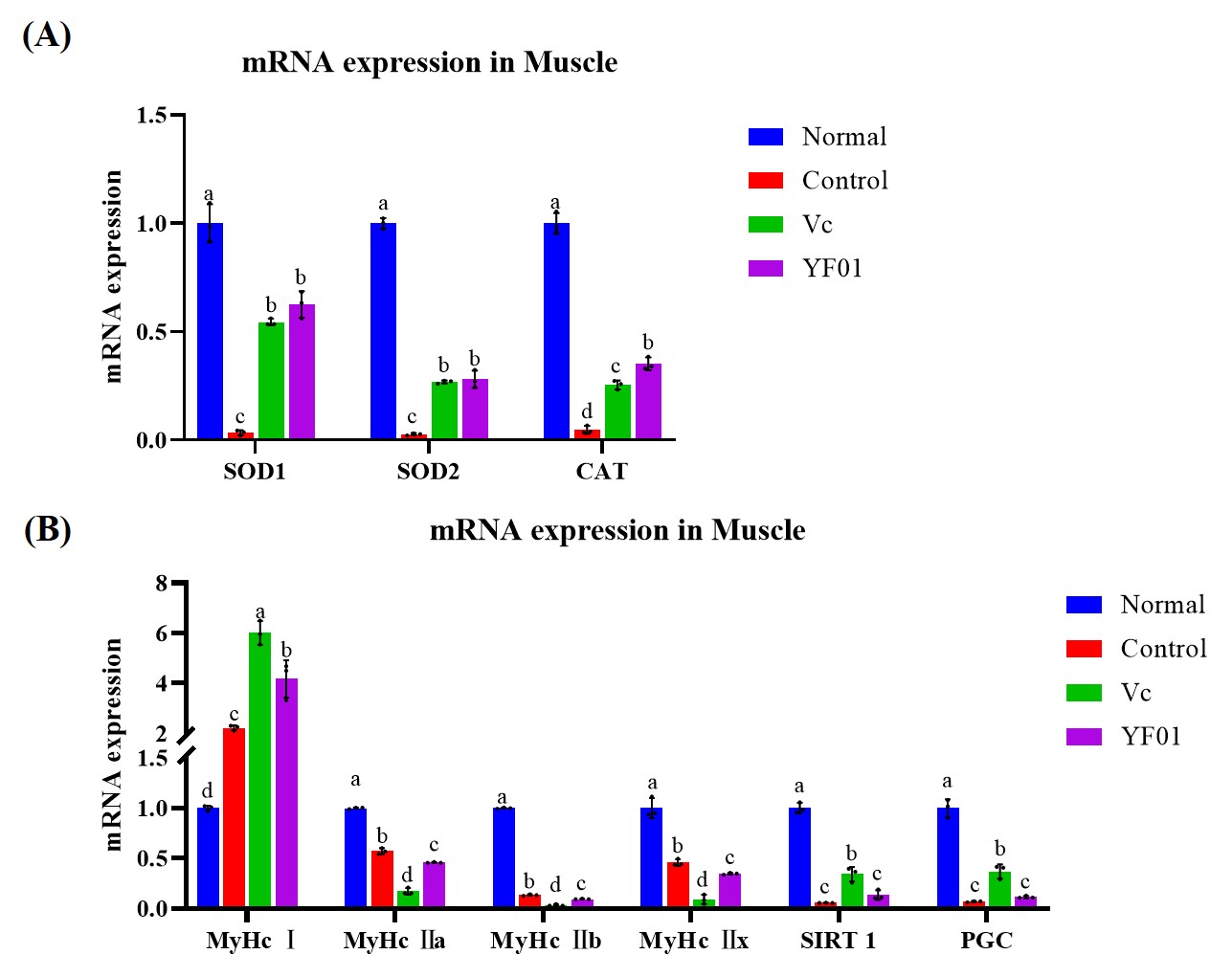

Supplement: Supplementary file 1 [file Data_Sheet_1.ZIP › All figure/FIGURE 9.jpg]
