# Supplementary material for: Impact of Pediococcus pentosaceus YF01 on the exercise capacity of mice through the regulation of oxidative stress and alteration of gut microbiota
Source: Front Microbiol. 2024 Jun 26;15:1421209. doi: 10.3389/fmicb.2024.1421209 (PMC11233450; doi:10.3389/fmicb.2024.1421209)
Supplement: Supplementary file 1 [file Data_Sheet_1.ZIP › Raw Data/gut microbiota/alpha__shannon.tar.gz!/alpha_asv_table_shannon_YF01L_tukey.result/shannon.pdf]

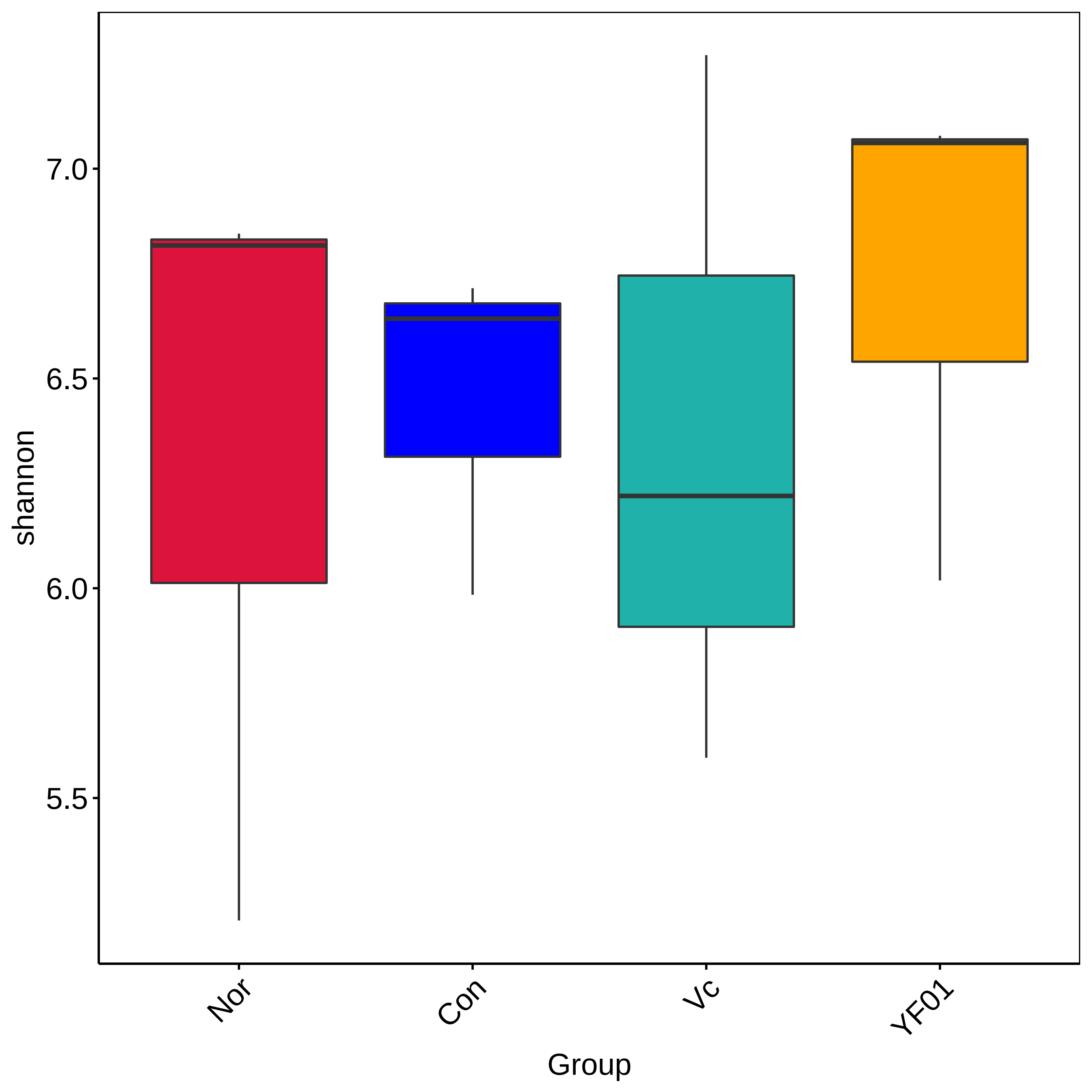
7.0
6.5
6.0
5.5
shannon
Nor
Con
Vc
YF01
Group
