# Supplementary material for: Impact of Pediococcus pentosaceus YF01 on the exercise capacity of mice through the regulation of oxidative stress and alteration of gut microbiota
Source: Front Microbiol. 2024 Jun 26;15:1421209. doi: 10.3389/fmicb.2024.1421209 (PMC11233450; doi:10.3389/fmicb.2024.1421209)
Supplement: Supplementary file 1 [file Data_Sheet_1.ZIP › Raw Data/gut microbiota/heatm_genus.tar.gz!/heatm_asv_table_YF01L_20_phylum,genus,species_group.genus.result/heatm_asv_table_YF01L_20_phylum,genus,species_group.genus.pdf]

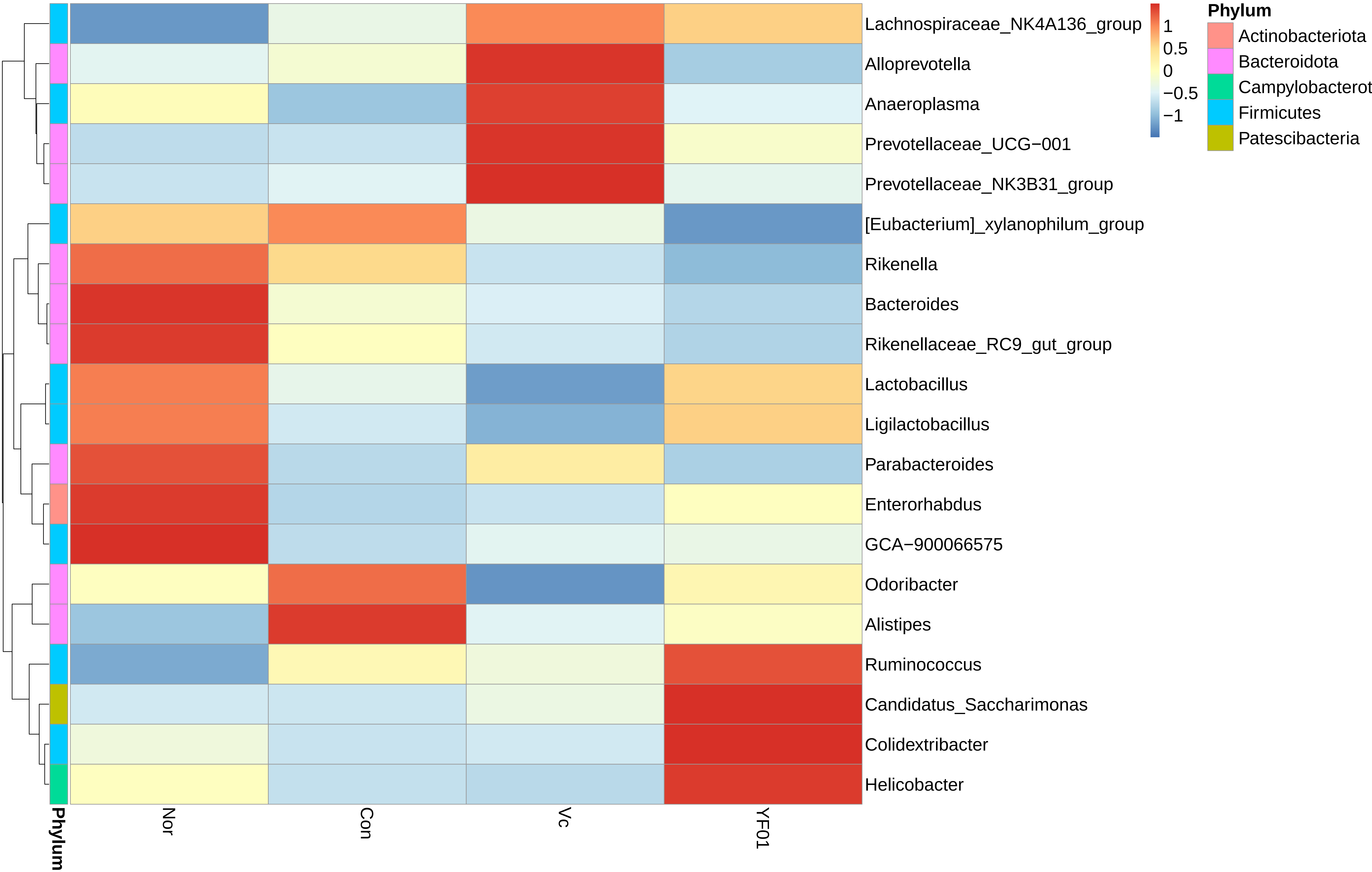

Lachnospiraceae_NK4A136_group
Alloprevotella
Anaeroplasma
Prevotellaceae_UCG−001
Prevotellaceae_NK3B31_group
[Eubacterium]_xylanophilum_group
Rikenella
Bacteroides
Rikenellaceae_RC9_gut_group
Lactobacillus
Ligilactobacillus
Parabacteroides
Enterorhabdus
GCA−900066575
Odoribacter
Alistipes
Ruminococcus
Candidatus_Saccharimonas
Colidextribacter
Helicobacter
Phylum
Nor
Con
Vc
YF01
1
0.5
0
−0.5
−1
Phylum
Actinobacteriota
Bacteroidota
Campylobacterot
Firmicutes
Patescibacteria
