# Supplementary material for: Impact of Pediococcus pentosaceus YF01 on the exercise capacity of mice through the regulation of oxidative stress and alteration of gut microbiota
Source: Front Microbiol. 2024 Jun 26;15:1421209. doi: 10.3389/fmicb.2024.1421209 (PMC11233450; doi:10.3389/fmicb.2024.1421209)
Supplement: Supplementary file 1 [file Data_Sheet_1.ZIP › Raw Data/gut microbiota/heatm_phylum.tar.gz!/heatm_asv_table_YF01L_20_phylum,genus,species_group.phylum.result/heatm_asv_table_YF01L_20_phylum,genus,species_group.phylum.pdf]

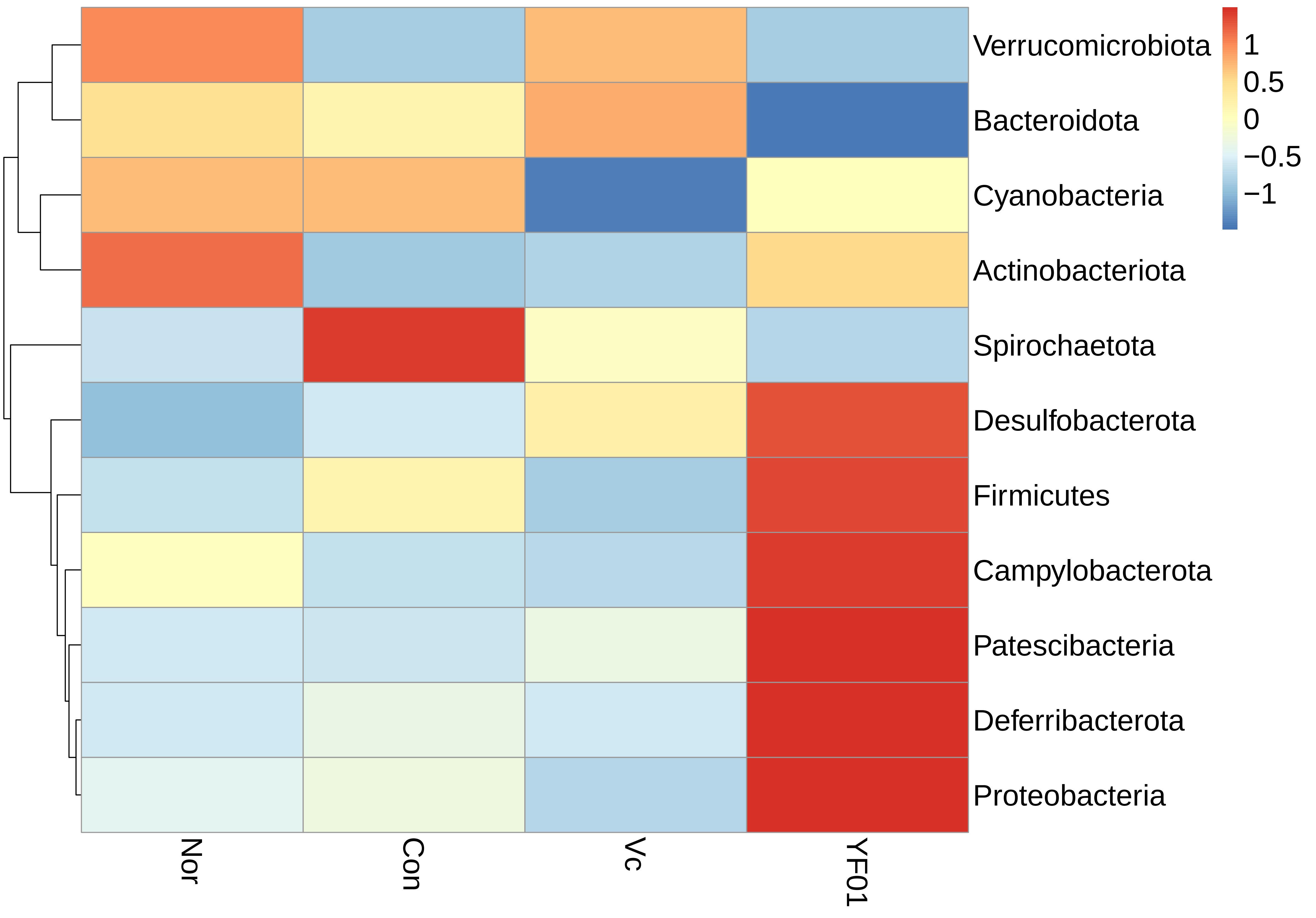

Verrucomicrobiota
Bacteroidota
Cyanobacteria
Actinobacteriota
Spirochaetota
Desulfobacterota
Firmicutes
Campylobacterota
Patescibacteria
Deferribacterota
Proteobacteria
Nor
Con
Vc
YF01
1
0.5
0
−0.5
−1
