# Supplementary material for: Impact of Pediococcus pentosaceus YF01 on the exercise capacity of mice through the regulation of oxidative stress and alteration of gut microbiota
Source: Front Microbiol. 2024 Jun 26;15:1421209. doi: 10.3389/fmicb.2024.1421209 (PMC11233450; doi:10.3389/fmicb.2024.1421209)
Supplement: Supplementary file 1 [file Data_Sheet_1.ZIP › Raw Data/gut microbiota/heatm_species.tar.gz!/heatm_asv_table_YF01L_20_phylum,genus,species_group.species.result/heatm_asv_table_YF01L_20_phylum,genus,species_group.species.pdf]

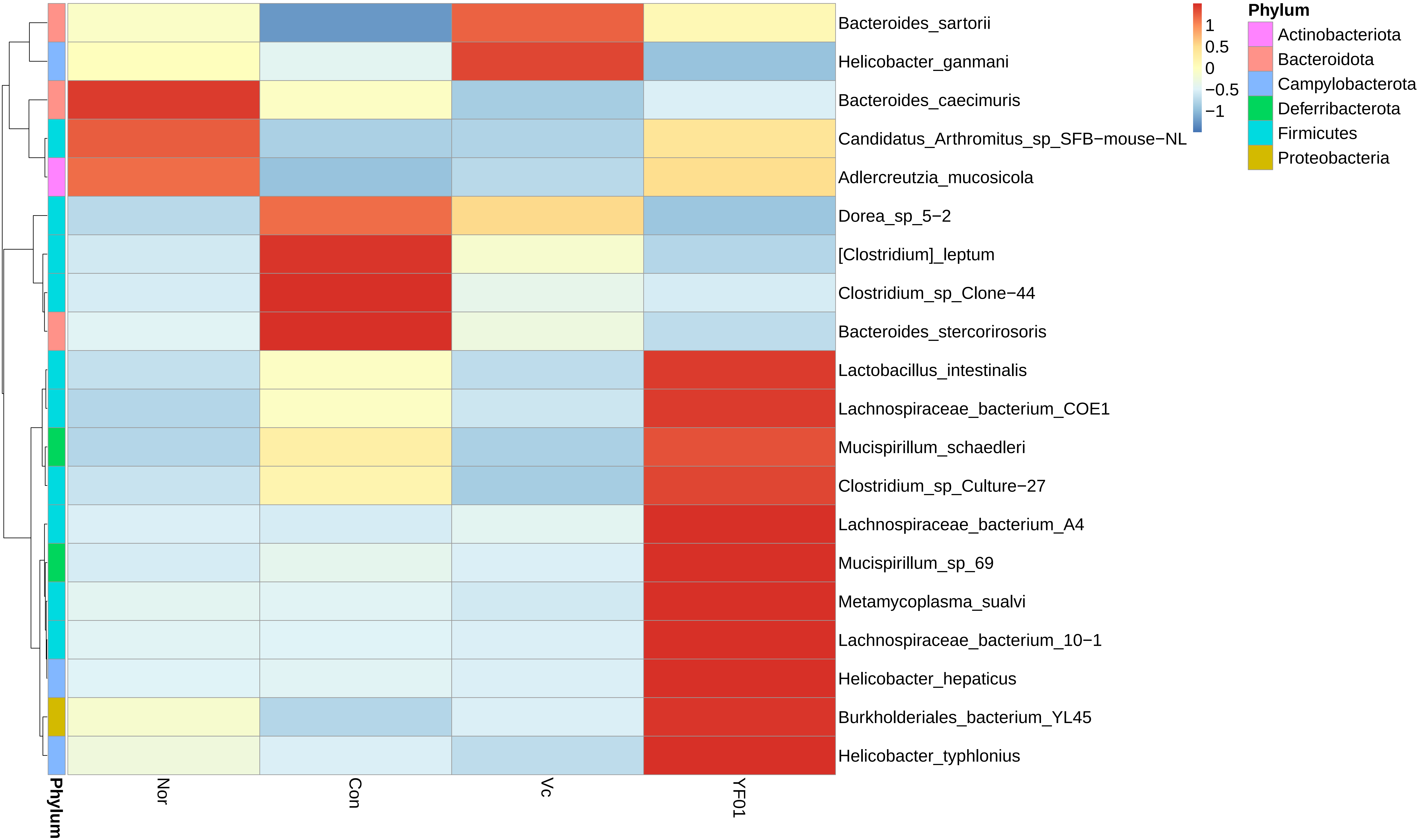
Bacteroides_sartorii
Helicobacter_ganmani
Bacteroides_caecimuris
Candidatus_Arthromitus_sp_SFB−mouse−NL
Adlercreutzia_mucosicola
Dorea_sp_5−2
[Clostridium]_leptum
Clostridium_sp_Clone−44
Bacteroides_stercorirosoris
Lactobacillus_intestinalis
Lachnospiraceae_bacterium_COE1
Mucispirillum_schaedleri
Clostridium_sp_Culture−27
Lachnospiraceae_bacterium_A4
Mucispirillum_sp_69
Metamycoplasma_sualvi
Lachnospiraceae_bacterium_10−1
Helicobacter_hepaticus
Burkholderiales_bacterium_YL45
Helicobacter_typhlonius
Phylum
Nor
Con
Vc
YF01
1
0.5
0
−0.5
−1
Phylum
Actinobacteriota
Bacteroidota
Campylobacterota
Deferribacterota
Firmicutes
Proteobacteria
