# Supplementary material for: Impact of Pediococcus pentosaceus YF01 on the exercise capacity of mice through the regulation of oxidative stress and alteration of gut microbiota
Source: Front Microbiol. 2024 Jun 26;15:1421209. doi: 10.3389/fmicb.2024.1421209 (PMC11233450; doi:10.3389/fmicb.2024.1421209)
Supplement: Supplementary file 1 [file Data_Sheet_1.ZIP › Raw Data/gut microbiota/LEfSe.tar.gz!/lefse_YF01_YF01_NorvsConvsVcvsYF01_2/Nor-vs-Con-vs-Vc-vs-YF01/biomarkers_raw_images/1_Bacteria-Firmicutes-Bacilli-Acholeplasmatales.pdf]

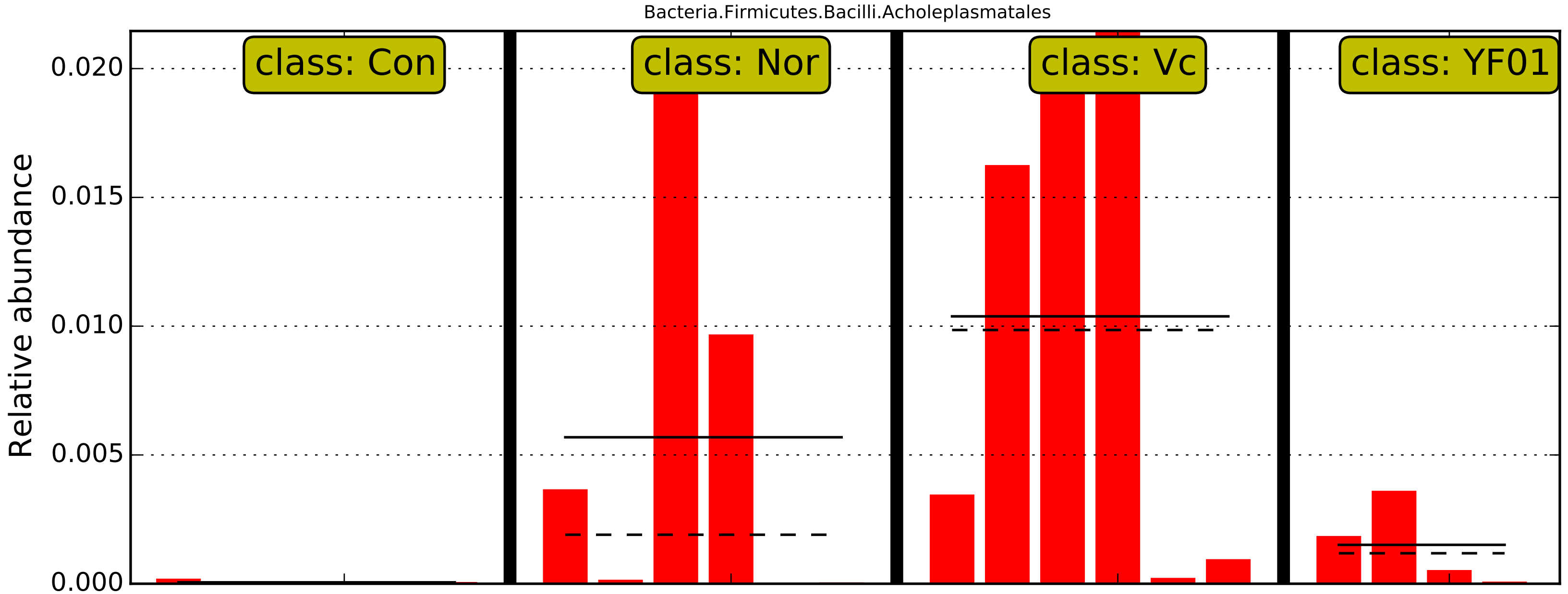
Bacteria.Firmicutes.Bacilli.Acholeplasmatales
Relative abundance
0.000
0.005
0.010
0.015
0.020
class: Con
class: Nor
class: Vc
class: YF01
