# Supplementary material for: Impact of Pediococcus pentosaceus YF01 on the exercise capacity of mice through the regulation of oxidative stress and alteration of gut microbiota
Source: Front Microbiol. 2024 Jun 26;15:1421209. doi: 10.3389/fmicb.2024.1421209 (PMC11233450; doi:10.3389/fmicb.2024.1421209)
Supplement: Supplementary file 1 [file Data_Sheet_1.ZIP › Raw Data/gut microbiota/LEfSe.tar.gz!/lefse_YF01_YF01_NorvsConvsVcvsYF01_2/Nor-vs-Con-vs-Vc-vs-YF01/biomarkers_raw_images/1_Bacteria-Firmicutes-Clostridia-Oscillospirales-Oscillospiraceae-UCG_007.pdf]

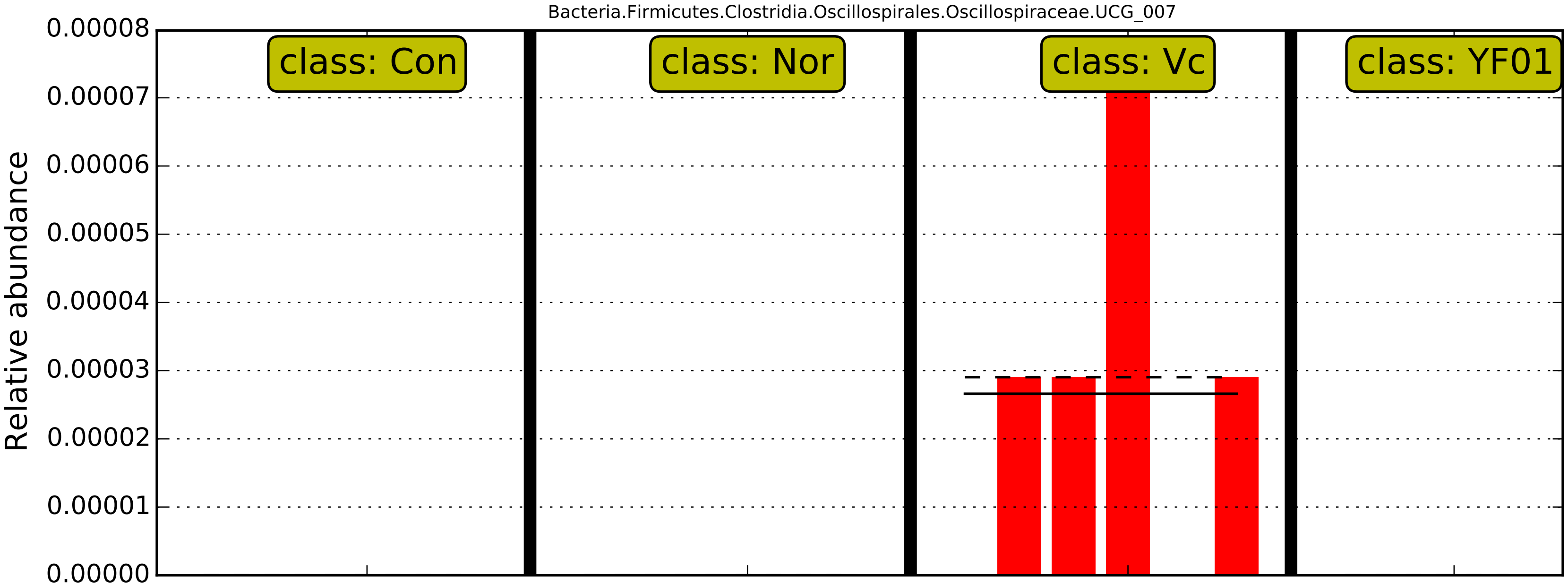
Bacteria.Firmicutes.Clostridia.Oscillospirales.Oscillospiraceae.UCG_007
Relative abundance
0.00008
0.00007
0.00006
0.00005
0.00004
0.00003
0.00002
0.00001
0.00000
class: Con
class: Nor
class: Vc
class: YF01
