# Supplementary material for: Impact of Pediococcus pentosaceus YF01 on the exercise capacity of mice through the regulation of oxidative stress and alteration of gut microbiota
Source: Front Microbiol. 2024 Jun 26;15:1421209. doi: 10.3389/fmicb.2024.1421209 (PMC11233450; doi:10.3389/fmicb.2024.1421209)
Supplement: Supplementary file 1 [file Data_Sheet_1.ZIP › Raw Data/gut microbiota/LEfSe.tar.gz!/lefse_YF01_YF01_NorvsConvsVcvsYF01_2/single_level/genus/LDA.pdf]

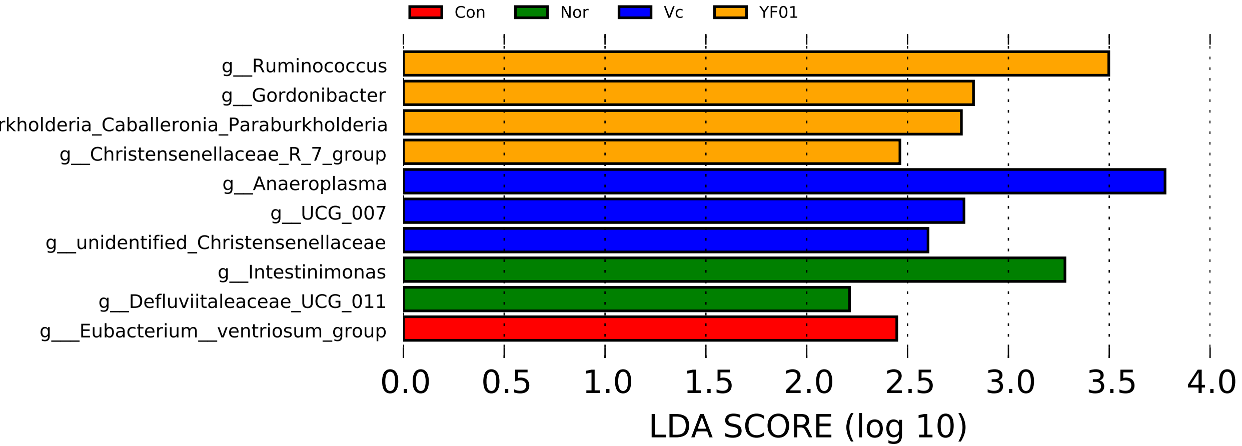

Con
Nor
Vc
YF01
g__Ruminococcus
g__Gordonibacter
rkholderia_Caballeronia_Paraburkholderia
g__Christensenellaceae_R_7_group
g__Anaeroplasma
g__UCG_007
g__unidentified_Christensenellaceae
g__Intestinimonas
g__Defluviitaleaceae_UCG_011
g___Eubacterium__ventriosum_group
0.0
0.5
1.0
1.5
2.0
2.5
3.0
3.5
4.0
LDA SCORE (log 10)
