# Supplementary material for: Impact of Pediococcus pentosaceus YF01 on the exercise capacity of mice through the regulation of oxidative stress and alteration of gut microbiota
Source: Front Microbiol. 2024 Jun 26;15:1421209. doi: 10.3389/fmicb.2024.1421209 (PMC11233450; doi:10.3389/fmicb.2024.1421209)
Supplement: Supplementary file 1 [file Data_Sheet_1.ZIP › Raw Data/gut microbiota/LEfSe.tar.gz!/lefse_YF01_YF01_NorvsConvsVcvsYF01_2/single_level/genus/LDA.tree.pdf]

Cladogram

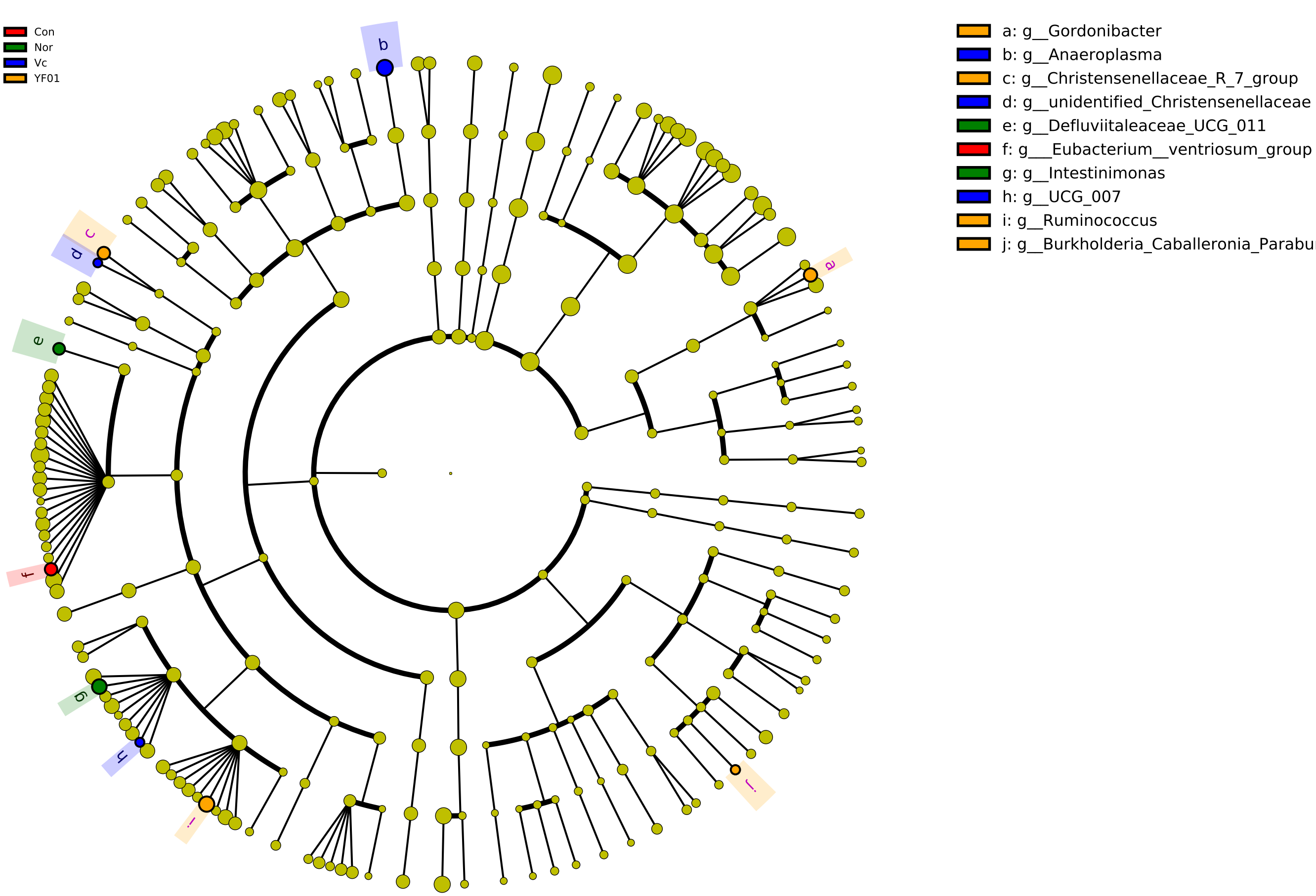
Con
Nor
Vc
YF01
a: g__Gordonibacter
b: g__Anaeroplasma
c: g__Christensenellaceae_R_7_group
d: g__unidentified_Christensenellaceae
e: g__Defluviitaleaceae_UCG_011
f: g___Eubacterium__ventriosum_group
g: g__Intestinimonas
h: g__UCG_007
i: g__Ruminococcus
j: g__Burkholderia_Caballeronia_Parabu
a
b
c
d
e
f
g
h
i
j
