# Supplementary material for: Impact of Pediococcus pentosaceus YF01 on the exercise capacity of mice through the regulation of oxidative stress and alteration of gut microbiota
Source: Front Microbiol. 2024 Jun 26;15:1421209. doi: 10.3389/fmicb.2024.1421209 (PMC11233450; doi:10.3389/fmicb.2024.1421209)
Supplement: Supplementary file 1 [file Data_Sheet_1.ZIP › Raw Data/gut microbiota/LEfSe.tar.gz!/lefse_YF01_YF01_NorvsConvsVcvsYF01_2/single_level/order/LDA.pdf]

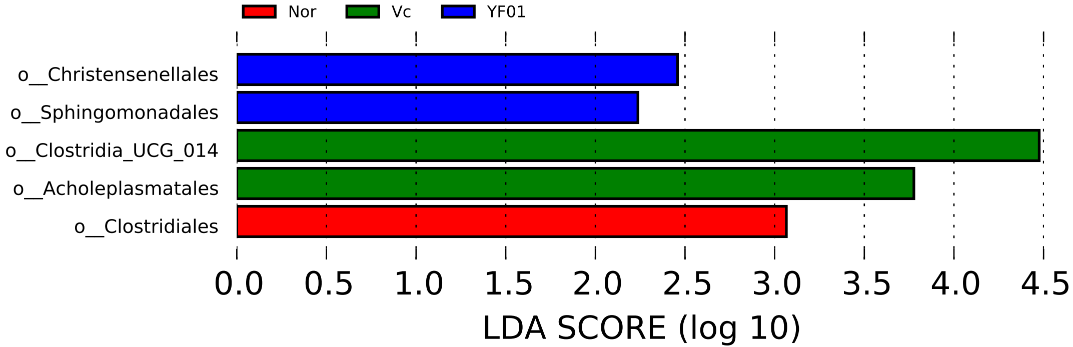

Nor
Vc
YF01
o__Christensenellales
o__Sphingomonadales
o__Clostridia_UCG_014
o__Acholeplasmatales
o__Clostridiales
0.0
0.5
1.0
1.5
2.0
2.5
3.0
3.5
4.0
4.5
LDA SCORE (log 10)
