# Supplementary material for: Impact of Pediococcus pentosaceus YF01 on the exercise capacity of mice through the regulation of oxidative stress and alteration of gut microbiota
Source: Front Microbiol. 2024 Jun 26;15:1421209. doi: 10.3389/fmicb.2024.1421209 (PMC11233450; doi:10.3389/fmicb.2024.1421209)
Supplement: Supplementary file 1 [file Data_Sheet_1.ZIP › Raw Data/gut microbiota/LEfSe.tar.gz!/lefse_YF01_YF01_NorvsConvsVcvsYF01_2/single_level/order/LDA.tree.pdf]

Cladogram

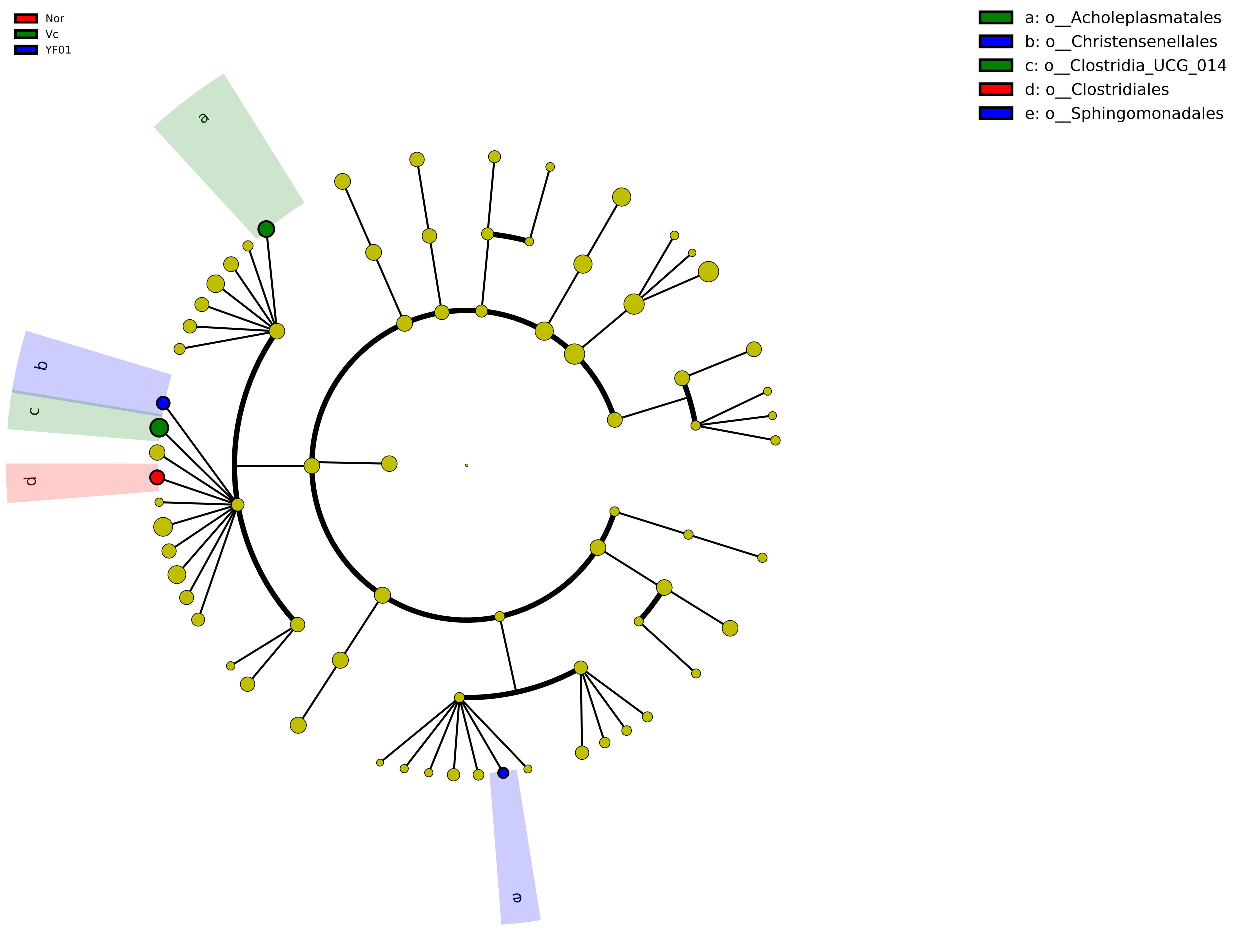

Nor
Vc
YF01
a: o__Acholeplasmatales
b: o__Christensenellales
c: o__Clostridia_UCG_014
d: o__Clostridiales
e: o__Sphingomonadales
a
b
c
d
e
