# Supplementary material for: Impact of Pediococcus pentosaceus YF01 on the exercise capacity of mice through the regulation of oxidative stress and alteration of gut microbiota
Source: Front Microbiol. 2024 Jun 26;15:1421209. doi: 10.3389/fmicb.2024.1421209 (PMC11233450; doi:10.3389/fmicb.2024.1421209)
Supplement: Supplementary file 1 [file Data_Sheet_1.ZIP › Raw Data/gut microbiota/LEfSe.tar.gz!/lefse_YF01_YF01_NorvsConvsVcvsYF01_2/single_level/species/LDA.pdf]

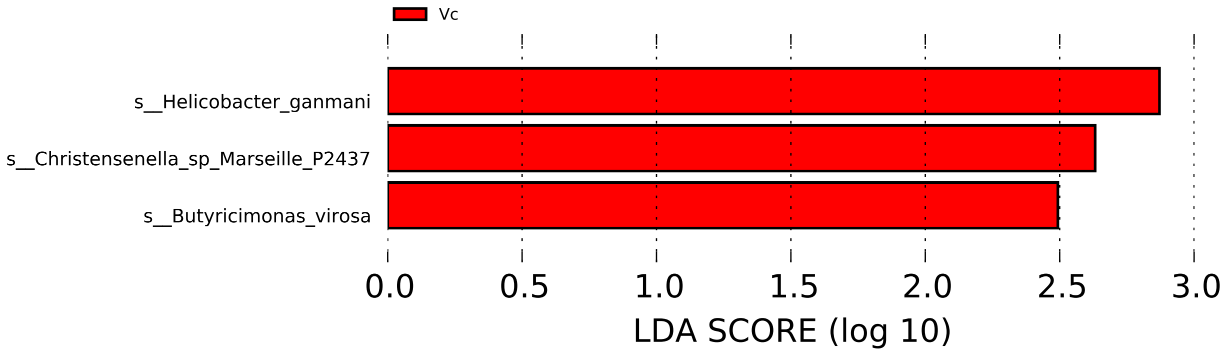
Vc
s__Helicobacter_ganmani
s__Christensenella_sp_Marseille_P2437
s__Butyricimonas_virosa
0.0
0.5
1.0
1.5
2.0
2.5
3.0
LDA SCORE (log 10)
