# Supplementary material for: Impact of Pediococcus pentosaceus YF01 on the exercise capacity of mice through the regulation of oxidative stress and alteration of gut microbiota
Source: Front Microbiol. 2024 Jun 26;15:1421209. doi: 10.3389/fmicb.2024.1421209 (PMC11233450; doi:10.3389/fmicb.2024.1421209)
Supplement: Supplementary file 1 [file Data_Sheet_1.ZIP › Raw Data/gut microbiota/pca.tar.gz!/pca_asv_table_YF01L.result/pca_asv_table_YF01L_cluster_2.pdf]

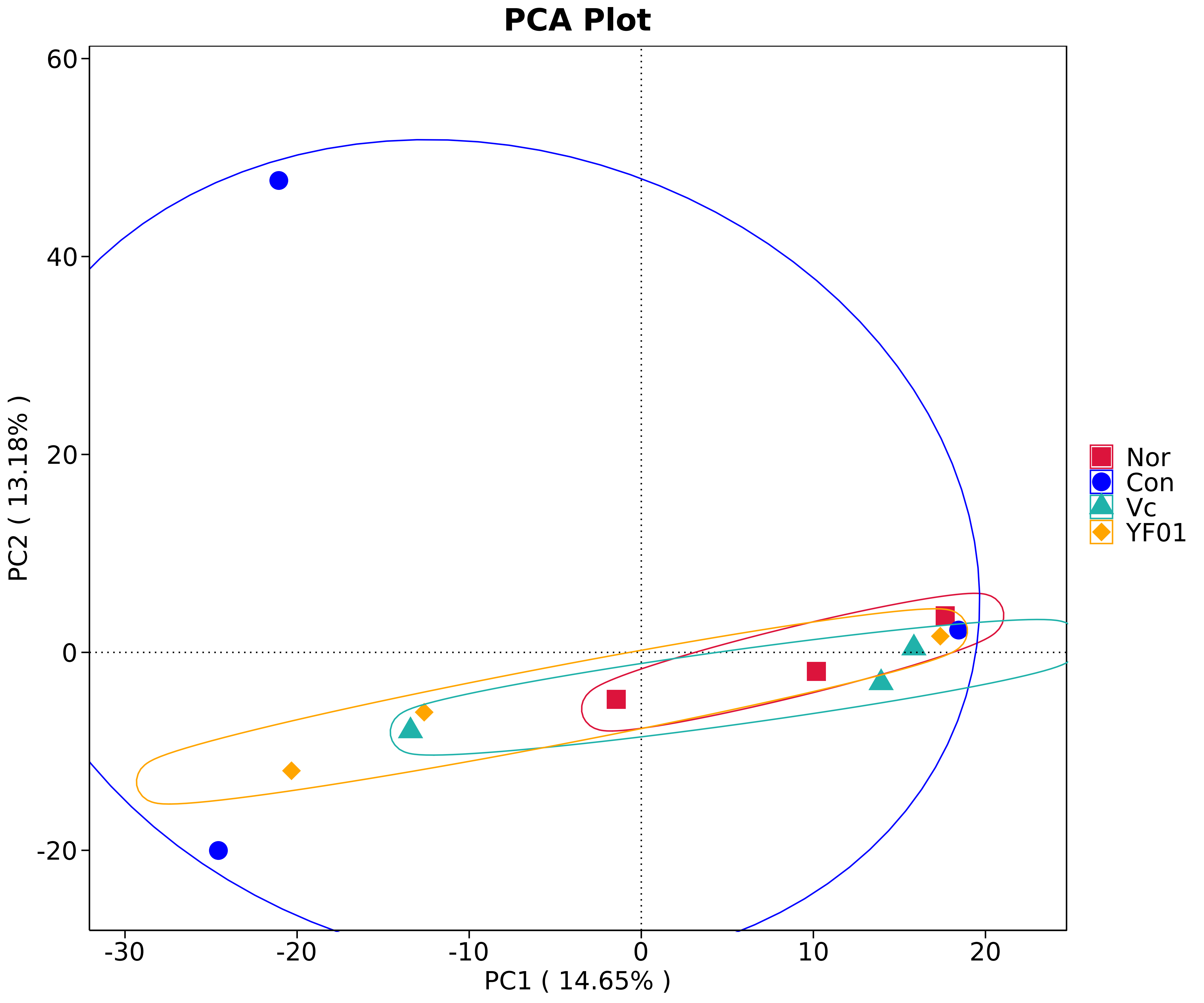

PCA Plot
60
40
20
0
-20
-30
-20
-10
0
10
20
PC1 (14.65%)
PC2 (13.18%)
Nor
Con
Vc
YF01
