# Supplementary material for: Impact of Pediococcus pentosaceus YF01 on the exercise capacity of mice through the regulation of oxidative stress and alteration of gut microbiota
Source: Front Microbiol. 2024 Jun 26;15:1421209. doi: 10.3389/fmicb.2024.1421209 (PMC11233450; doi:10.3389/fmicb.2024.1421209)
Supplement: Supplementary file 1 [file Data_Sheet_1.ZIP › Raw Data/gut microbiota/top_genus.tar.gz!/top_asv_table_YF01L_20_phylum,genus,species_group.genus.result/top_asv_table_YF01L_20_phylum,genus,species_group.genus_no_others.pdf]

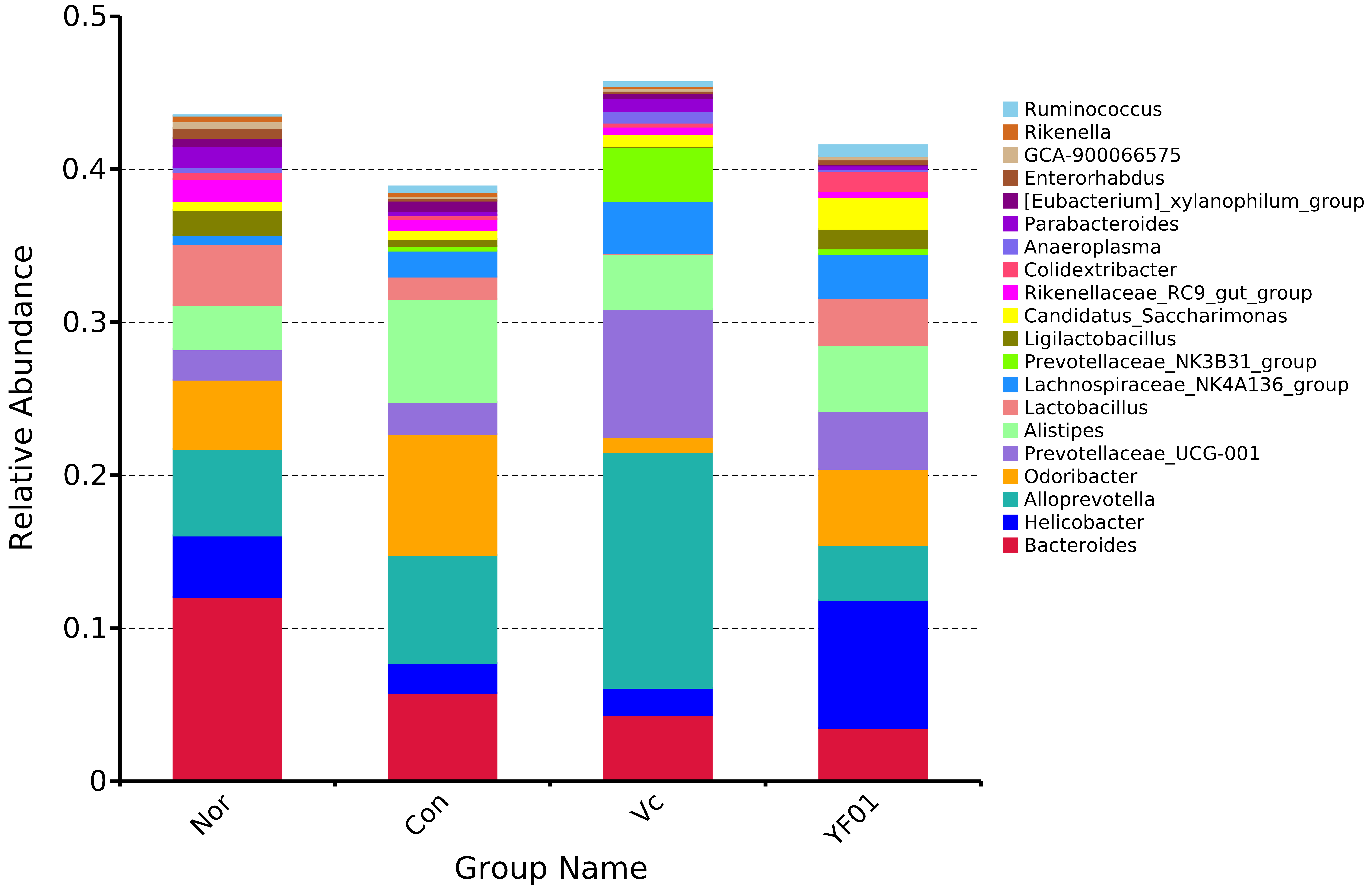

0.5
0.4
0.3
0.2
0.1
0
Relative Abundance
Nor
Con
Vc
YF01
Group Name
Ruminococcus
Rikenella
GCA-900066575
Enterorhabdus
[Eubacterium]_xylanophilum_group
Parabacteroides
Anaeroplasma
Colidextribacter
Rikenellaceae_RC9_gut_group
Candidatus_Saccharimonas
Ligilactobacillus
Prevotellaceae_NK3B31_group
Lachnospiraceae_NK4A136_group
Lactobacillus
Alistipes
Prevotellaceae_UCG-001
Odoribacter
Alloprevotella
Helicobacter
Bacteroides
