# Supplementary material for: Impact of Pediococcus pentosaceus YF01 on the exercise capacity of mice through the regulation of oxidative stress and alteration of gut microbiota
Source: Front Microbiol. 2024 Jun 26;15:1421209. doi: 10.3389/fmicb.2024.1421209 (PMC11233450; doi:10.3389/fmicb.2024.1421209)
Supplement: Supplementary file 1 [file Data_Sheet_1.ZIP › Raw Data/gut microbiota/top_phylum.tar.gz!/top_asv_table_YF01L_20_phylum,genus,species_group.phylum.result/top_asv_table_YF01L_20_phylum,genus,species_group.phylum.pdf]

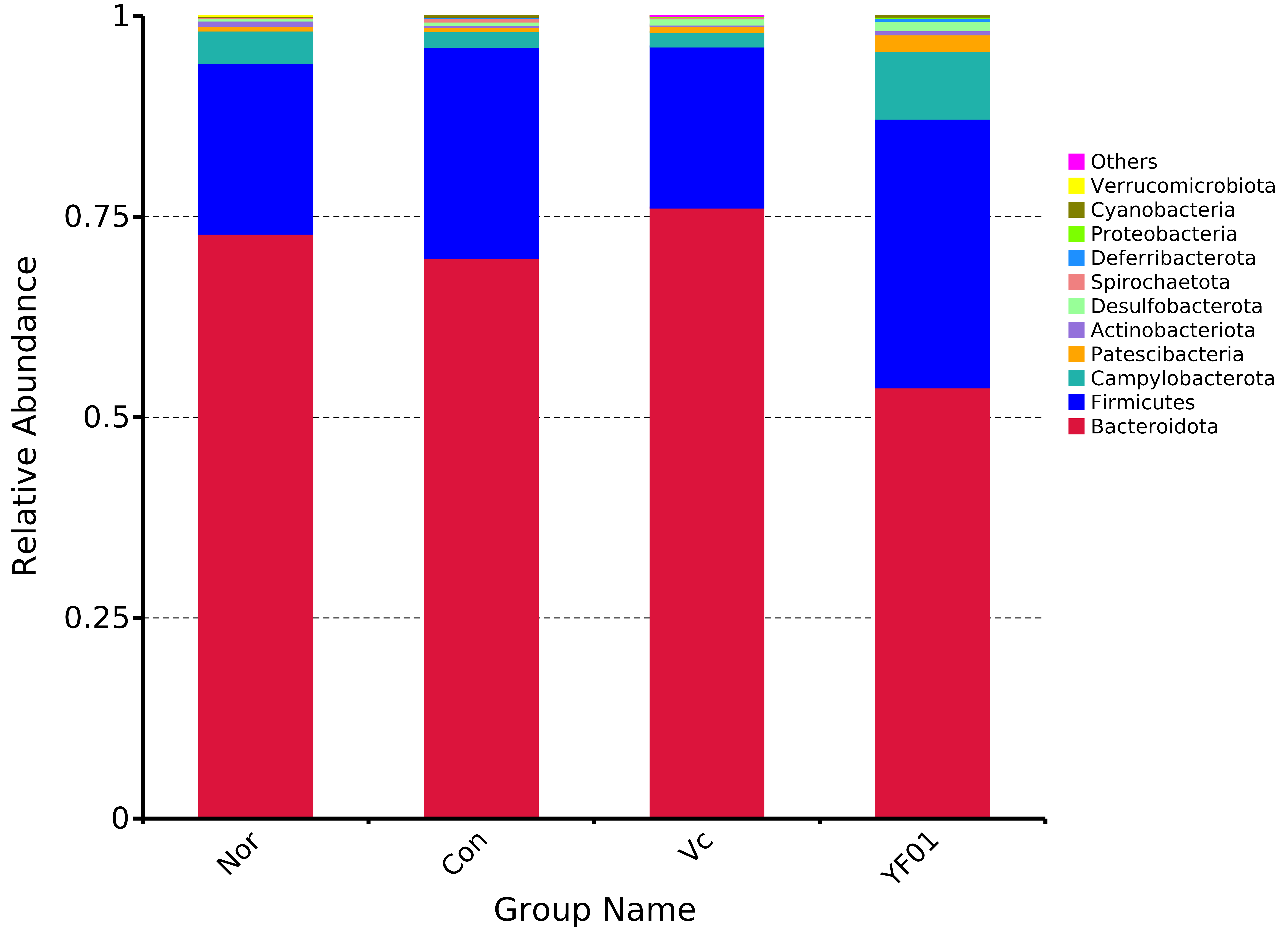

Relative Abundance
1
0.75
0.5
0.25
0
Nor
Con
Vc
YF01
Group Name
Others
Verrucomicrobiota
Cyanobacteria
Proteobacteria
Deferribacterota
Spirochaetota
Desulfobacterota
Actinobacteriota
Patescibacteria
Campylobacterota
Firmicutes
Bacteroidota
