# Supplementary material for: Impact of Pediococcus pentosaceus YF01 on the exercise capacity of mice through the regulation of oxidative stress and alteration of gut microbiota
Source: Front Microbiol. 2024 Jun 26;15:1421209. doi: 10.3389/fmicb.2024.1421209 (PMC11233450; doi:10.3389/fmicb.2024.1421209)
Supplement: Supplementary file 1 [file Data_Sheet_1.ZIP › Raw Data/gut microbiota/venn.tar.gz!/flower_asv_table_YF01L_group.1.result/flower_asv_table_YF01L_group.1.pdf]

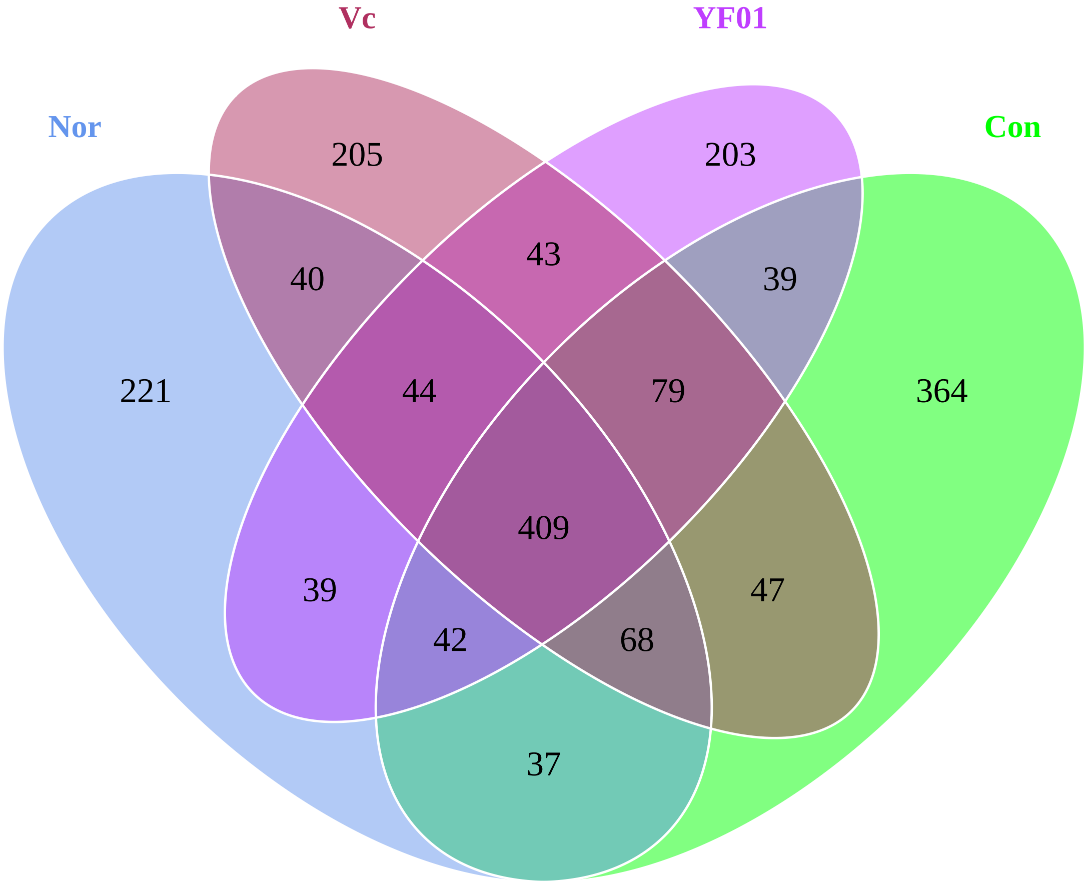

Vc
YF01
Nor
Con
205
203
43
40
39
221
44
79
364
409
39
47
42
68
37
